# Integrated proteogenomic characterization of ampullary adenocarcinoma

**DOI:** 10.1038/s41421-024-00742-4

**Published:** 2025-01-07

**Authors:** Qiao Zhang, Xiaomeng Xu, Dongxian Jiang, Yunzhi Wang, Haixing Wang, Jiajun Zhu, Shaoshuai Tang, Ronghua Wang, Shuang Zhao, Kai Li, Jinwen Feng, Hang Xiang, Zhenmei Yao, Ning Xu, Rundong Fang, Wenjia Guo, Yu Liu, Yingyong Hou, Chen Ding

**Affiliations:** 1 Center for Cell and Gene Therapy, Clinical Research Center for Cell-based Immunotherapy, Shanghai Pudong Hospital, State Key Laboratory of Genetic Engineering, School of Life Sciences, Human Phenome Institute, Fudan University, Shanghai, 200433 China; 2 Department of Pathology, Zhongshan Hospital Fudan University, Shanghai, China; 3 Pediatric Translational Medicine Institute, Shanghai Children’s Medical Center, School of Medicine, Key Laboratory of Pediatric Hematology & Oncology Ministry of Health, Department of Hematology & Oncology, Shanghai Jiao Tong University, Shanghai, China; 4 Departments of Cancer Research Institute, Affiliated Cancer Hospital of Xinjiang Medical University, Xinjiang Key Laboratory of Translational Biomedical Engineering, Urumqi, Xinjiang China

**Keywords:** Gastrointestinal cancer, Molecular biology

## Abstract

Ampullary adenocarcinoma (AMPAC) is a rare and heterogeneous malignancy. Here we performed a comprehensive proteogenomic analysis of 198 samples from Chinese AMPAC patients and duodenum patients. Genomic data illustrate that 4q loss causes fatty acid accumulation and cell proliferation. Proteomic analysis has revealed three distinct clusters (C-FAM, C-AD, C-CC), among which the most aggressive cluster, C-AD, is associated with the poorest prognosis and is characterized by focal adhesion. Immune clustering identifies three immune clusters and reveals that immune cluster M1 (macrophage infiltration cluster) and M3 (DC cell infiltration cluster), which exhibit a higher immune score compared to cluster M2 (CD4^+^ T-cell infiltration cluster), are associated with a poor prognosis due to the potential secretion of IL-6 by tumor cells and its consequential influence. This study provides a comprehensive proteogenomic analysis for seeking for better understanding and potential treatment of AMPAC.

## Introduction

Ampullary adenocarcinoma (AMPAC) is a rare malignant neoplasm that forms in an area called the ampulla of Vater. AMPAC with an incidence of ~4–7 cases per 1,000,000 people^1^, accounts for ~7% of all periampullary cancers and 0.2% of gastrointestinal cancers^2,3^. The 5-year survival rate ranges from 20%–75%, based on different stages of cancer progression^4,5^. Histologically, AMPAC is separated into intestinal-type, pancreatobiliary-type, or mixed-type, and the intestinal-type patients have a better prognosis than the pancreatobiliary-type patients^6^. Due to its characteristics of an unknown etiology, extremely low incidence rate, and complex anatomical structure, ampullary adenocarcinoma has long been a challenging subject for clinical and pathological research, and multi-omics cohort studies.

The incidence of AMPAC remains unknown, and targeted drugs and treatments are lacking^7^. The prevailing therapeutic regimen for AMPAC primarily entails the utilization of pancreaticoduodenectomy^8^, with some chemotherapy and radiation therapy after surgery^9,10^. According to the differences in cellular origins and immunohistochemistry (IHC) between intestinal-type and pancreatobiliary-type patients, the intestinal-type patients were starting to be treated with the fluorouracil-based regimen, and the pancreatobiliary-type patients were treated with the gemcitabine-based regimen. Nevertheless, the chemotherapy seems ineffective and only relies on the subtype that was estimated by cellular morphology and immunohistochemistry.

Two previous genomic cohort studies revealed the gene mutation pattern of AMPAC and assessed the driver mutated genes involved in tumorigenesis, including *TP53*, *KRAS*, *APC*, *SMAD4*, *ARID2*, *CTNNB1*, and *ELF3*^11,12^. In addition, Yachida et al.^9^ and Ginsgras et al.^10^ also revealed changes in alterations in WNT signaling, RTK/RAS, and TGF-β signaling pathway in AMPAC, which have a similar alteration frequency with our data (Supplementary Fig. S1i). However, current genomic studies have not yet elucidated the intratumoral biological mechanisms of AMPAC, such as pathway alterations influenced by copy number variations (CNVs), which still require further investigation. Thus, the integrated study contained data from both proteome and genomic alterations that would be necessary to uncover the molecular characteristics of AMPAC.

In this study, we included tumor samples from 198 patients along with paired adjacent non-tumor samples. Due to the location of AMPAC, these 198 samples also included 12 cases of duodenal cancer (DAC), which were very close to the location of AMPAC. Proteogenomic analysis unveiled the downstream pathways impacted by CNV events. At the level of chromosomal alterations in the AMPAC, the loss of 4q occurred frequently, and the low expression level of HADH (the *cis* effect in 4q) leads to the accumulation of fatty acids, consequently inducing cell proliferation. For the focal event, 9p21.3 deletion and 5p22.1 deletion were also found to be risk factors for AMPAC. A genetic study has disclosed several frequent mutations including *KRAS*, *TP53*, *APC*, *ARID2*, *SMAD4*, *CTNNB1*, and *ELF3*, et al. The comparative analysis illustrated the distinctive pathway differences between pancreatobiliary-type and intestinal-type and identified potential therapeutic targets, PCNA for intestinal-type and ANO1 for pancreatobiliary-type AMPAC. Based on the proteomic classification, three clusters with distinctive features were established and the cluster with the poorest prognosis was characterized by enrichment of focal adhesion. Immune infiltration analysis indicated that a higher immune score was accompanied by a poorer prognosis in AMPAC patients. Based on the multi-omics analysis of AMPAC, our study can serve as a valuable dataset supporting biological discoveries and provide insights for therapeutic development in the future.

## Results

### Comprehensive proteogenomic characterization of AMPAC samples

**Fig. 1:**
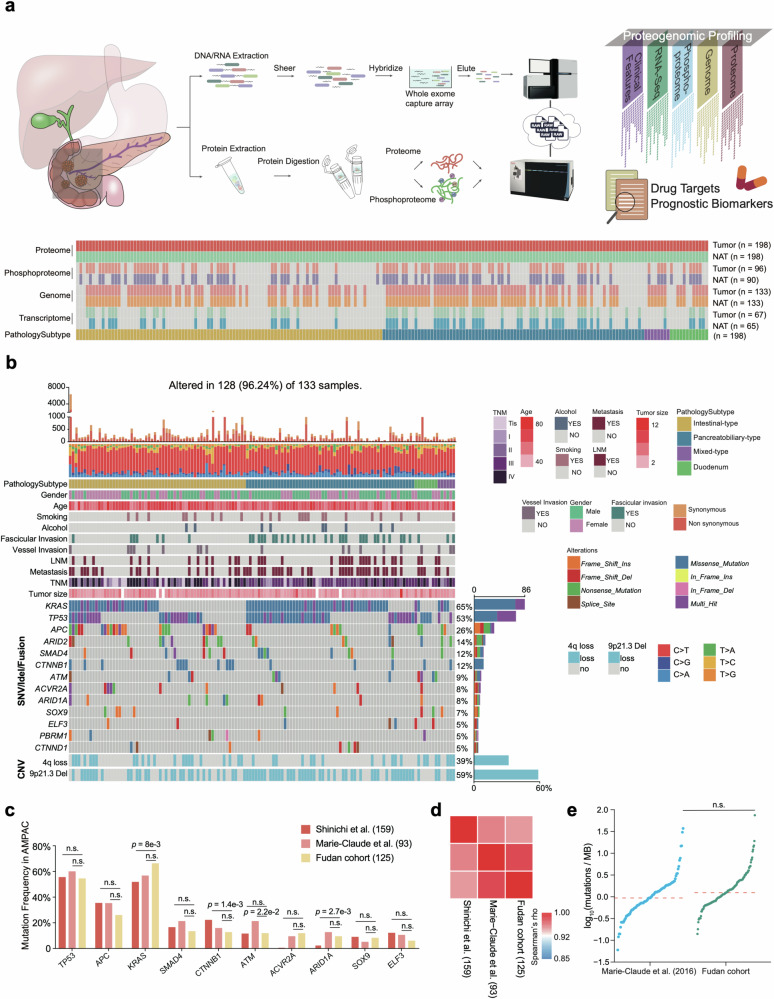
Proteogenomic landscape of AMPAC. **a** The workflow of the experiment. Top panel, overview of the experimental design and the number of samples for the genomic, transcriptomic, proteomic, and phosphoproteomic analyses. Bottom panel, sample numbers and multi-omics datasets of Fudan cohort. **b** Profile of significantly mutated genes (SMGs) and associated clinical features of patients with AMPAC. SMGs in this dataset identified by MutSigCV and OncodriveCLUST (*q* value < 0.1) are shown. Top panel, number of mutations per sample. Middle panel, the clinical characteristics of each sample and distribution of significant mutations across the sequenced samples, color-coded by mutation type. Bottom panel, the distribution of SCNVs across the sequenced samples. Frequent focal somatic copy-number variations. Right panel, percentage of samples affected. **c** Comparisons of mutation frequencies of top 10 mutated genes in the Fudan cohort and previously published cohorts. **d** Correlation plot of the mutation frequencies observed in the Fudan cohort compared to those in previously published cohorts (Spearman correlation). **e** Comparison of TMB in the tumors of our cohort and the Marie-Claude’s cohort.

We had established an extensive multi-omics research cohort (Fudan cohort) for AMPAC on a global scale. To systematically define the proteogenomic landscape of AMPAC, we collected formalin-fixed paraffin-embedded (FFPE) tumor samples and paired normal adjacent tissues (NATs) from 186 AMPAC patients and 12 DAC patients spanning from 2008 to 2017. A total of 186 AMPAC patients comprised 96 intestinal-type patients, 82 pancreatobiliary-type patients, and 8 mixed-type patients (Fig. 1a, Supplementary Fig. S1a and Table S1a). The examination and assessment of HE-stained slides were performed independently by two experienced pathologists, who provided details about the tumor’s histological subtype, degree of differentiation, and TNM stage (Supplementary Table S1a). Clinical attributes, including the age at diagnosis, gender, tumor grade, etc., are summarized based on clinicopathological features in Supplementary Table S1a. Demographically, all patients entitled in this study were from Asia. Other risk factors and information associated with patient prognoses were also collected via follow-up in this study (Supplementary Table S1a). The Whole-exome sequencing (WES) analysis was conducted on 133 tumor and 133 NAT samples. RNA sequencing (RNA‐seq) was performed for the transcriptome analysis on 67 tumor and 65 NAT samples. For all 198 paired tumor and NAT samples, a mass spectrometry (MS)-based proteomic analysis was carried out. In addition, phosphoproteomic analysis was performed on 96 tumor samples and 90 NAT samples using a Fe-NTA phosphopeptides enrichment strategy (Fig. 1a and Supplementary Fig. S1a).

WES led to 110‐fold mean target coverage and identified 9108 mutated genes, including 14,233 non-silent point mutations and 1599 small insertions or deletions. A total of 14,150 somatic mutations with a median rate of 15.85 coding mutations per megabase. Significantly mutated genes (SMGs) were identified using MutSigCV. Among the 133 patients, we observed several SMGs, including *KRAS* (65%), *TP53* (53%), *APC* (26%), *ARID2* (14%), *SMAD4* (12%), *CTTNB1* (12%), *ATM* (9%), *ACVR2A* (8%), *ARID1A* (8%), *SOX9* (7%), *ELF3* (5%), *PBRM1* (5%), and *CTTND1* (5%) (Fig. 1b and Supplementary Table S1b). Among these SMGs, the mutational frequency of *APC* was higher in intestinal-type patients, and *KRAS* mutation was significantly higher in pancreatobiliary-type patients. Correlation analysis was performed using mutational frequencies from Yachida’s work and Gingras’s work (Fisher’s exact test *p* < 0.05; Fig. 1c)^11,12^, and Spearman correlation reflected similar mutational profiles across the three cohorts (Fig. 1d). The previous studies also revealed changes in alterations in *WNT* signaling, *RTK/RAS* signaling, and *TGF-β* signaling pathways in AMPAC, which have a similar alteration frequency in our data (Supplementary Fig. S1i). Additionally, there was a similar TMB between Fudan cohort and Gingras’s cohort (Fig. 1e).

Non-negative matrix factorization (NMF) was employed to assess the frequencies of mutated trinucleotide sequence motifs^13,14^. We identified five mutational signatures by Sigminer. These 5 signatures corresponded to the known COSMIC (Catalog of Somatic Mutations in Cancer) signatures: SBS30 (Defective DNA base excision repair due to NTHL1 mutations, *n* = 100), SBS20 (Concurrent POLD1 mutations and defective DNA mismatch repair, *n* = 2), SBS6 (Defective DNA mismatch repair, *n* = 15), SBS2 (Activity of APOBEC family of cytidine deaminases, *n* = 15), and SBS18 (Damage by reactive oxygen species, *n* = 1) (Supplementary Fig. S1j). The overall proportions of single nucleotide variants (SNVs) were similar to those observed in other cohorts^11,12^, with cytosine to thymine (C > T) transition being the most frequent SNV. Transcriptome sequencing identified 15,328 genes with fragments per kilobase of transcript per million fragments mapped (FPKM) values > 1. A total of 15,196 and 14,847 transcripts were identified in the tumor and NAT samples, respectively (Supplementary Fig. S1d, e).

For the proteomics analysis, whole-cell extracts of human embryonic kidney-derived HEK293T cells were utilized as controls to ensure data quality. This showed the robustness and consistency of the mass spectrometer, which is evidenced by a high Spearman correlation coefficient (*r* > 0.9) between the proteomes of QC samples (Supplementary Fig. S1h). Additionally, proteomic analysis identified 15,363 proteins in total and 14,280 and 13,447 proteins in the tumor and NAT samples, respectively. We then applied quality control for whole proteomic data and filtered the proteins with less than 1% FDR. As a result, 13,092 proteins were utilized for further analysis (Supplementary Fig. S1b–e).

Phosphoproteomics analysis identified 28,714 phosphosites including 21,088 (73.4%) on serine, 6836 (23.8%) on threonine, 790 (2.8%) on tyrosine; from 5964 phosphoproteins in 96 tumor samples, 22,776 phosphosites including 16,721 (73.4%) on serine, 5421 (23.8%) on threonine, 634 (2.8%) and on tyrosine, from 5147 phosphoproteins in 90 NAT samples (Supplementary Fig. S1e). The ratio of S/T/Y in this research is similar to the phosphorylation site S/Y/T distribution among CRC cohort (serine: 76.2%, threonine: 19.9%, tyrosine: 3.9%)^15^, GC cohort (serine: 74.0%, threonine: 20.9%, tyrosine: 5.1%)^16^, HCC cohort (serine: 77.8%, threonine: 16.9%, tyrosine: 5.3%)^17^, PDAC cohort (serine: 73.2%, threonine: 23.7%, tyrosine: 3.1%)^18^, indicating that the ratio of S/T/Y in AMPAC is comparable (Supplementary Fig. S1f, g). In total, our study has presented systematic molecular characteristics of AMPAC at the multi-omics level.

Thus, our study has so far established a comprehensive landscape of AMPAC at the multi-omics levels (Fig. 1a and Supplementary Fig. S1a).

### Impact of somatic copy number variations in the AMPAC Fudan cohort

**Fig. 2:**
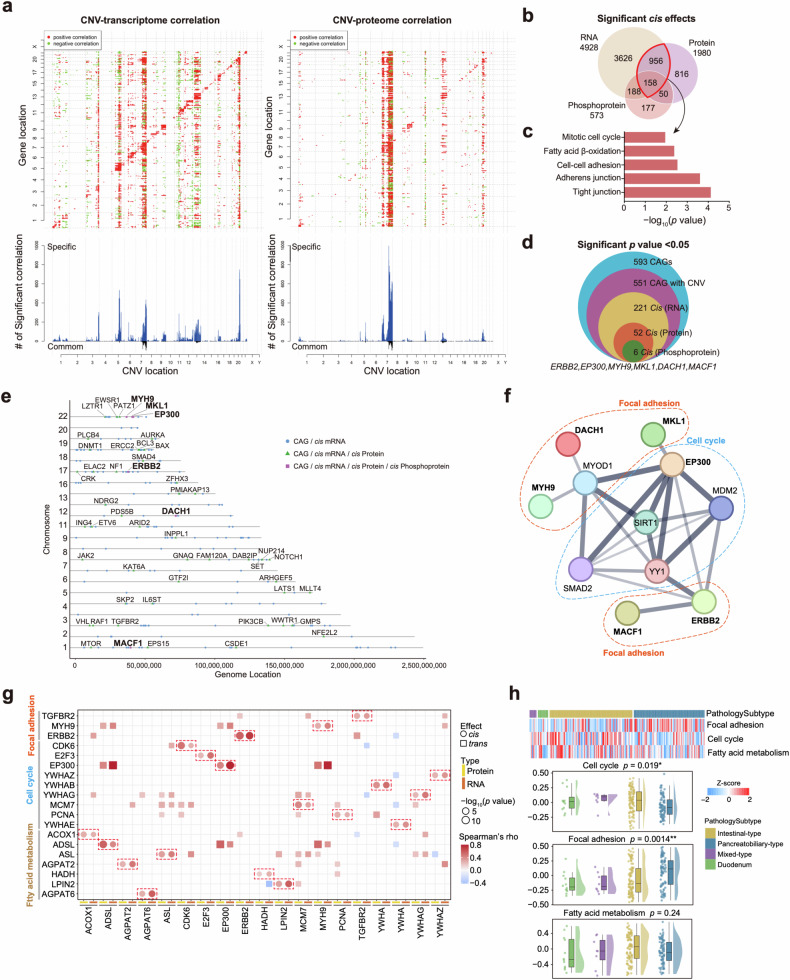
Impacts of somatic copy number variations in AMPAC Fudan cohort. **a** Functional effect of CNVs on mRNA and proteins. Top panels, the correlations of CNV to mRNA and protein abundance. Positive and negative correlations are indicated in red and green, respectively. Genes were ordered by chromosomal location on the *x* and *y* axes. Diagonal lines indicate *cis* effects of CNV on mRNA or proteins. Bottom panels, the numbers of mRNAs or proteins that were significantly associated with a specific CNV. Gray bars indicate correlations specific to mRNA or proteins, and black bars indicate correlations with both mRNA and proteins. **b** Venn diagrams depicting the cascading effects of CNVs and the overlap between *cis* events via the transcriptome, proteome, and phosphoproteome analyses (Spearman’s correlation, *p* < 0.05). **c** The pathways enriched by 1114 significant *cis* effect genes overlapped with the transcriptome and proteome. **d** Venn diagram showing the CAGs with significant CNV *cis* effects via multi-omics data analyses (Spearman’s correlation, *p* < 0.05). **e** Locations of the CAGs with cascading copy number *cis* regulation of their cognate mRNA, protein, and phosphoprotein levels. **f** The protein–protein interaction network constructed by the 6 CAGs. **g** *Cis* and *trans* effects of significant *cis* effect genes from focal adhesion, cell cycle, and fatty acid metabolism pathways. **h** Top panel, heatmap of the GSVA score of focal adhesion, cell cycle, and fatty acid metabolism pathways in the different pathology subtypes. Bottom panel, boxplot of the GSVA score of focal adhesion, cell cycle, and fatty acid metabolism pathways in the different pathology subtypes (one-way ANOVA test).

The impacts of copy-number variations (CNVs) on mRNA, protein, and phosphoprotein abundances in both *cis* and *trans* modes were characterized (Fig. 2a). A total of 4928, 1980, and 573 significant correlations (*cis* effects) were observed for mRNA, proteins, and phosphoproteins, respectively (Supplementary Table S2e). GO pathway analysis indicated consistency among pathways subjected to enrichment by CNV-affected 1114 mRNAs and proteins, which were enriched in pathways related to tight junction, adherens junction, cell–cell adhesion fatty acid β-oxidation, and cell cycle (Fig. 2b, c and Supplementary Table S2e). These reflected CNV impact on the signaling pathway. We investigated the impact of CNVs on mRNA, protein and phosphoprotein abundances of 593 cancer-associated genes (CAGs)^19^ via either *cis* or *trans* effects. Our analysis revealed that CNVs have *cis* effects on both the mRNA and protein abundances of 52 CAGs, as well as on three omics level abundances of 6 CAGs (*ERBB2*, *EP300*, *MYH9*, *MKL1*, *DACH1*, and *MACF1*) (Fig. 2d), and the annotations for these 6 CAGs are shown in Fig. 2e. Additionally, according to the STRING database, these 6 CAGs that had previously demonstrated direct or indirect interactions with each other, and primarily impacted the focal adhesion and cell cycle pathways (Fig. 2f). The *cis* effects and *trans* effects correlation of these three pathways are shown in Fig. 2g. Gene Set Variation Analysis (GSVA) was utilized to analyze these pathways enrichment. Cell cycle was significantly upregulated in the intestinal-type (One-way ANOVA test, *p* = 0.019), and focal adhesion was highly enriched in the pancreatobiliary-type (one-way ANOVA test, *p* = 0.0014) (Fig. 2h). The results suggest that pathway differences are impacted by different CNVs in two histological subtypes.

Gistic2.0 was employed to analyze the somatic copy-number alteration profiles of 133 AMPAC samples. The most frequent gains were identified in chromosomes 20q (1.8e−5), 13q (3.1e−3) and 7q (9.7e−4), and the most losses were found in chromosomes 9p (4.88e−14), 17p (4.54e−11), and 4q (3.6e−3) (Supplementary Fig. S2a and Table S2a). The 4q loss was the only event at the chromosome arm level that was negatively correlated with overall survival (OS) (HR = 2, *p* = 0.019) (Supplementary Table S2b). Furthermore, we detected focal alterations and identified 51 significant (*q* < 0.1) amplified peaks and 125 significant (*q* < 0.1) deleted peaks (Supplementary Fig. S2b). In addition, we identified focal events in driver oncogenes, including *MTAP* (9p21.3 deletion, 78 cases), *KRAS* (16q23.1 deletion, 28 cases) and *TYK2* (19p13.2 deletion, 49 cases), which were negatively associated with OS (Hazard ratio (HR) > 1, *p* < 0.05) (Supplementary Fig. S2b, c and Table S2c, d).

Among them, 9p21.3 deletion was particularly prevalent in Fudan cohort. *MTAP*, located on 9p21.3, showed a significant *cis* effect on its protein and mRNA expression levels, which encoded S-methyl-5’-thioadenosine phosphorylase (Supplementary Fig. S2d and Table S2g). Both IHC staining and qualification of IHC results presented the downregulation of MTAP in samples with 9p21.3 deletion compared to the WT samples (Supplementary Fig. S2e, f). MTAP serves as the pivotal enzyme responsible for cleaving MTA in the methionine salvage pathway^20^ (Supplementary Fig. S2g). In MTAP-deficient tumor cells, the accumulation of MTA within cells binds to PRMT5, forming the PRMT5/MTA complex. This complex inhibits PRMT5 activity and enhances the sensitivity of PRMT5 gene inhibition^21,22^. Thus, inhibition of PRMT5 was predicted to recapitulate the selective synthetic lethality observed in MTAP knockdown cells^23^. We utilized siRNA to knock down the expression of MTAP in the AMPAC cell line (SNU-478) (Supplementary Fig. S2h), and treated them with MRTX-1719, a potent and selective binder to the PRMT5/MTA complex that could selectively inhibit PRMT5 activity in MTAP-deleted cells^24,25^. The effects of MRTX-1719 on cell viability were measured. Consistently, MTAP-knockdown cells were more sensitive to MRTX-1719 with lower IC_50_ values (median IC_50_: 3.7 μM in MTAP-knockdown cells vs 7.0 μM in SNU-478 cells) (Supplementary Fig. S2i). This result indicated the applicability of the synthetic lethal inhibitor MRTX-1719 in AMPAC, and proposed a potential therapeutic approach for AMPAC MTAP-deleted patients (Supplementary Fig. S2j).

Although tyrosine phosphorylations were a small fraction of the total phosphoprotein, they played important roles in cell proliferation and suppression of the immune environment. Therefore, we examined the RTK phosphorylation data in this study. The phosphoproteomic analysis identified 914 tyrosine phosphosites in total, with 602 tyrosine phosphosites from 477 phosphoproteins in 96 tumor samples, and 495 phosphosites from 407 phosphoproteins in 90 NAT samples, respectively (Supplementary Fig. S2k). To explore the influence of tyrosine phosphorylation on the downstream signaling pathway, we performed differential tyrosine phosphosites analysis, and 22 differential expressed tyrosine phosphosites were identified. Among them, 15 tyrosine phosphosites had a significantly higher expression abundance in tumor samples and 7 tyrosine phosphosites had a significantly higher expression abundance in NAT samples (Supplementary Fig. S2l). Additionally, we employed pathway enrichment analysis on the 602 phosphoproteins in 96 tumor samples and 407 phosphoproteins in 90 NAT samples. As a result, we found these phosphoproteins were enriched in pathways such as Focal adhesion, ErbB signaling pathway, PD-L1 expression and PD-1check points pathway in tumor samples, and regulation of actin cytoskeleton, platelet activation, and motor proteins were enriched in NAT samples (Supplementary Fig. S2m).

*ERBB2* and *EGFR* were recurrently amplified in AMPAC^26^. To investigate these 2 amplifications impact on AMPAC tumorigenesis, we calculated the Spearman correlation to assess the relationship between the copy number of *EGFR/ERBB2* and their corresponding protein, mRNA, and phosphoprotein expression abundances. Herein, we found that *EGFR* and *ERBB2* were both amplified frequently in our study, of which both had the *cis*-effects at the protein level, and *ERBB2* had the *cis* effect at the mRNA level (Supplementary Fig. S2n). However, we did not observe that *EGFR* amplification could increase the kinase activity of EGFR, nor did we observe that ERBB2 amplification could increase the kinase activity of ERBB2 (Supplementary Fig. S2n)^27,28^. Therefore, *ERBB2* and *EGFR* amplification mainly affected the downstream signaling pathway by their protein expression abundance. Besides, we checked the correlation between *EGFR/ERBB2* amplification and the prognosis of ampullary adenocarcinoma. Although the result showed the copy number of *EGFR/ERBB2* showed a worse prognosis than the low copy number patients, the results showed no statistical significance between the copy number of *EGFR/ERBB2* and the prognosis of ampullary adenocarcinoma (Supplementary Fig. S2o, p).

### 4q loss-induced fatty acid metabolism disruption promotes cell proliferation

**Fig. 3:**
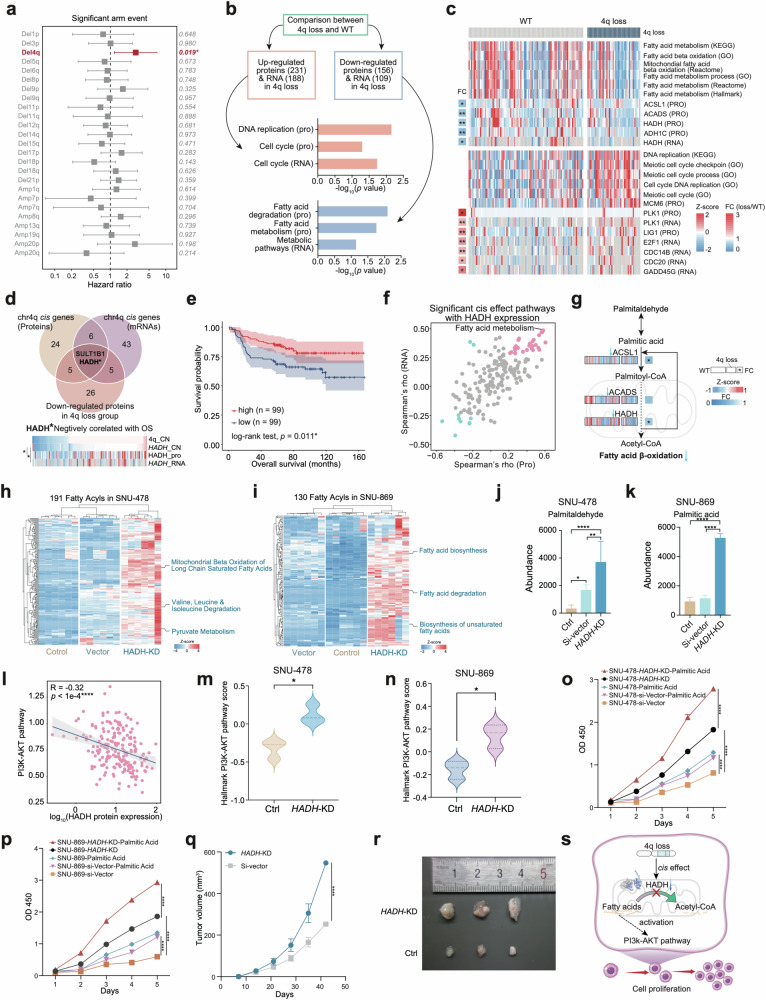
4q loss induced fatty acid metabolism disruption promotes cell proliferation. **a** Forest plot showed the hazard ratios of the significant arm events (*q* value < 0.1), *p* values were listed on the right. **b** Comparison between the 4q loss group and the WT group. Top panel, selection of significantly different expressed genes (FC > 1.5, *p* < 0.05) at both the mRNA and protein levels. Middle panel, pathways enriched by genes that upregulated proteins and mRNAs in 4q loss group. Bottom panel, pathways enriched by genes that downregulated proteins and mRNAs in 4q loss group. **c** Heatmap of the GSVA scores of pathways enriched by differentially expressed genes in **b**. **d** Screening of the *cis* gene on chromosome 4q. Top panel, selection of candidate *cis* genes on 4q according to expression level in the 4q loss group compared with WT group and correlation with the overall survival. Bottom panel, heatmap showing the HADH copy number, protein expression, and mRNA abundance. **e** Kaplan–Meier curves for overall survival based on the protein expression of HADH (log-rank test, *p* = 0.011). **f** The scatter plot showed the correlations between GSVA scores of the KEGG pathways and the expression of HADH at both proteins and mRNA levels. The *x*-axis represented the Spearman’s correlations at the protein level, and the *y*-axis represented the Spearman’s correlations at mRNA level. Positive correlates were colored pink, and negative correlates were colored blue (Spearman’s *p* value < 0.05). **g** Schematic diagram of the comparison between the 4q loss group and the WT group of fatty acid metabolism pathway in AMPAC. **h** Heatmap of MS-based 191 fatty acyls in SNU-478 cells and the pathway enrichment of the 191 metabolites were shown on the right. **i** Heatmap of MS-based 130 fatty acyls in SNU-869 cells and the pathway enrichment of the 130 metabolites were shown on the right. **j** Bar plot illustrated the abundance of palmitaldehyde in the control, empty vector, and HADH-KD groups in SNU-478 according to the MS-based untargeted metabolomics result (two-way ANOVA test). **k** Bar plot illustrated the abundance of palmitic acid in the control, empty vector, and HADH-KD groups in SNU-869 according to the MS-based untargeted metabolomics result (two-way ANOVA test). **l** The scatter plot described the correlation between HADH protein expression and the GSVA score of the PI3K-AKT pathway (Spearman’s *r* = 0.32, *p* < 1e−4). **m** The violin plot of the GSVA score of Hallmark PI3K-AKT pathway in the control group and HADH-KD group in SNU-478 (Student’s *t*-test, *p* < 0.05). **n** The violin plot of the GSVA score of Hallmark PI3K-AKT pathway in the control group and HADH-KD group in SNU-869 (Student’s *t*-test, *p* < 0.05). **o** Proliferation of SNU-478 cells associated with various treatments (*n* = 5, mean ± SEM, two-sided Student’s *t*-test). **p** Proliferation of SNU-869 cells associated with various treatments (*n* = 3, mean ± SEM, two-sided Student’s *t*-test). **q** Xenograft tumor volumes of HADH-KD groups and empty vector groups (SNU-478). **r** Xenograft tumor images of the SNU-478 cells subcutaneously injected into nude mice. **s** The systematic diagram summarized the impact of dysregulation of fatty acid metabolism due to the 4q loss promotes cell proliferation.

Notably, chromosome 4q loss was the only event at the chromosome arm level that was negatively correlated with overall survival (Fig. 3a and Supplementary Fig. S3a). To investigate the *cis* effect of 4q loss, KEGG pathway enrichment analysis was utilized on differentially expressed proteins (DEPs) in the 4q loss group and WT group at both the mRNA and protein levels. A total of 231 proteins and 188 mRNAs (FC (4q loss/WT) > 1.5, *p* < 0.05) were upregulated in the 4q loss group, which were enriched in cell cycle at both the protein and mRNA levels, while 156 proteins and 109 mRNAs (FC (4q loss/WT) > 1.5, *p* < 0.05) were downregulated in the 4q loss group, which were enriched in fatty acid metabolism and fatty acid degradation at the protein level and metabolic pathways at the mRNA level (Fig. 3b). We also discovered genes involved in fatty acid metabolism (such as *ACSL1*, *ACADS*, *HADH,* and *ADH1C*) were downregulated in the 4q loss group, which suggests a weakening of the ability of fatty acid β-oxidation in AMPAC (Fig. 3c and Supplementary Table S2f). To examine the influence of fatty acid metabolism on the prognosis of AMPAC patients, we performed survival analysis according to the GSVA scores of fatty acid metabolism. The results indicated that patients with lower GSVA scores had worse prognosis (Supplementary Fig. S3i).

To explore how 4q loss impacts fatty acid metabolism, we examined all genes located on 4q loss that had *cis* effect on both protein and mRNA levels, and we found the copy number of *HADH* was significantly positive correlated with its mRNA and protein expression abundance (mRNA: Spearman’s *r* = 0.29, *p* = 0.03, protein: Spearman’s *r* = 0.23, *p* = 0.0075, Fig. 3d and Supplementary Fig. S3f, g). This means that with the copy number alteration of 4q loss, the *HADH* also experienced a loss of copy number, and both the HADH protein and mRNA expression abundance also decreased with corresponding trends. Besides, we found that HADH was the only protein whose low expression abundance was associated with a poorer prognosis in AMPAC (log-rank test, *p* = 0.019, Fig. 3e) and displayed a downregulation in the 4q loss group compared to the WT group (Supplementary Fig. S3b, c). Both IHC staining and qualification of IHC results presented the downregulation of HADH in samples with 4q loss compared to the WT samples (Supplementary Fig. S3d, e). *HADH* encodes hydroxyacyl-coenzyme A dehydrogenase, which plays a role in fatty acid metabolism by participating in fatty acid degradation/β-oxidation pathway. It was found to be downregulated in hepatocellular carcinoma (HCC) and kidney renal clear cell carcinoma (KIRC) coinciding with the downregulation of the fatty acid β-oxidation pathway^29–31^. Significantly positive correlations between the HADH expression and the GSVA score of fatty acid metabolism at both the protein and mRNA levels were also observed in our cohort (Fig. 3f, g). These findings suggested that the downregulation of fatty acid metabolism was the consequence of HADH downregulation.

To further elucidate the impact of accumulated long-chain fatty acids resulting from downregulated HADH, we transfected the AMPAC cell lines SNU-478 and SNU-869 with *HADH* siRNA (SNU-478-*HADH*-KD, SNU-869-*HADH*-KD) and scrambled siRNA (SNU-478-Si-Vector, SNU-869-Si-Vector as a control, and then performed comparative proteomic analysis^32^. *HADH* mRNA levels were significantly downregulated in the SNU-478-*HADH*-KD group (Supplementary Fig. S3j). We also evaluated the concentration of metabolites in SNU-478-*HADH*-KD and SNU-869-*HADH*-KD cells, and conducted a pathway enrichment analysis of these metabolites. The lipid metabolites, accumulated in SNU-478-*HADH*-KD cells, were associated with mitochondrial β-oxidation of long-chain saturated fatty acids, valine, leucine, and isoleucine degradation and pyruvate metabolism pathways (Fig. 3h); the lipid metabolites detected in SNU-869-*HADH*-KD cells were involved with fatty acid biosynthesis, fatty acid degradation and biosynthesis of unsaturated fatty acids pathways (Fig. 3i). Notably, palmitaldehyde, the initial substrate for fatty acid β-oxidation, was significantly accumulated in SNU-478-*HADH*-KD cells (Fig. 3j); the palmitic acid was identified a high level in the SNU-869-*HADH*-KD cells (Fig. 3k). These results suggested that the downregulation of HADH repressed fatty acid β-oxidation resulting in the accumulation of long chain fatty acids in the AMPAC. Consequently, we proposed that the accumulation of long-chain fatty acids (palmitic acid/palmitaldehyde) could be a characteristic feature within the 4q loss AMPAC patients.

Previous study illustrated that the reduction of HADH-mediated gastric cancer showed a deceleration of β-oxidation that leads to the accumulation of fatty acids, activating the PI3K-Akt signaling pathway, which often promotes malignant tumor growth^33^. We sought to investigate whether HADH downregulation activates the PI3K-Akt signaling pathway in AMPAC tumor cells. To explore this, we performed a correlation analysis between the protein expression of HADH and the GSVA score of PI3K-Akt signaling pathway. The HADH expression was positively associated with the GSVA score of the PI3K-Akt signaling pathway (Fig. 3l). Additionally, we utilized MS-based proteomic analysis to nominate the proteins that participate in PI3K-Akt signaling pathway, and performed differential analysis in SNU-478-*HADH*-KD and SNU-869-*HADH*-KD cells and the control groups cells. The result revealed the upregulation of the PI3K-Akt signaling pathway in the SNU-478-*HADH*-KD and SNU-869-*HADH*-KD groups compared to the control (Fig. 3m, n). These illustrated that HADH downregulation followed with PI3K-Akt signaling pathway elevation in AMPAC. A previous study described that tumor proliferation was promoted by activating the PI3K-Akt signaling pathway^33^. Given the observed upregulation of the cell cycle in the 4q loss group as illustrated in Fig. 3b, c, it is plausible that HADH downregulation could induce the accumulation of long-chain fatty acids, and may activate the PI3K-Akt signaling pathway, thereby promoting cell proliferation in AMPAC.

To validate whether HADH downregulation could promote tumor cell growth, we conducted the cell growth ability assessment on SNU-478-*HADH*-KD and SNU-869-*HADH*-KD cells. The results displayed a significantly increased proliferation ability in SNU-478-*HADH*-KD and SNU-869-*HADH*-KD cells compared to the control (Fig. 3o, p). The palmitic acid supplementation increased proliferation ability in SNU-478-*HADH*-KD and SNU-869-*HADH*-KD groups, and this increase was significantly higher than that observed in the control groups (Fig. 3o, p). This suggests that palmitic acid could promote the proliferation ability in SNU-478-*HADH*-KD and SNU-869-*HADH*-KD groups. Furthermore, it demonstrated that HADH downregulation leads to the accumulation of fatty acids, which indeed facilitates proliferation ability in the *HADH*-KD cells. We further constructed xenograft mouse models using *HADH*-KD and control group cells, and conducted subcutaneous tumor experiments in mice. The downregulation of HADH was found to significantly promote xenograft growth compared to the control (Fig. 3q, r). These results provided additional confirmation that the accumulation of long-chain fatty acids resulting from the low expression of HADH promoted tumor cell proliferation.

In summary, we concluded that in AMPAC tumor cells, the downregulation of HADH expression, mediated by *cis* effect resulting from chromosome 4q loss, disrupted the metabolism of long-chain fatty acids. The accumulated long-chain fatty acids served as signaling stimuli that activated the PI3K-Akt signaling pathway, ultimately triggering cell proliferation (Fig. 3s).

### Integrated multi-omics features in tumor tissues compared with NATs of the AMPAC

Generating multi-omics profiles from both tumors and NATs provided a valuable opportunity to comprehensively investigate the interplay among AMPAC’s transcriptome, proteome, and phosphoproteome during tumorigenesis, offering insights into multi-omics remodeling. Principal component analysis (PCA) of RNA-seq data (14,131 genes) and proteome data (10,002 proteins) showed a clear distinction between tumors and NATs (Supplementary Fig. S4a, b and Table S3a). A total of 3128 genes and 2605 genes were upregulated (FC (T/N) > 1.5, *p* < 0.05) in tumors on both the proteome and transcriptome levels, respectively (Supplementary Fig. S4c, d). Further KEGG enrichment analysis indicated that in tumor tissues, both at the protein and mRNA levels, pathways such as ECM-receptor interaction, focal adhesion, PI3K-Akt signaling, and p53 signaling were significantly enriched (Supplementary Fig. S4e and Table S3b). These pathways were similar to those that we discovered previously (Fig. 2c). In contrast, pathways including fatty acid degradation, PPAR signaling, the citrate cycle, glycolysis/gluconeogenesis, and retinol metabolism were significantly enriched in NATs at both the protein and mRNA levels (Supplementary Fig. S4f and Table S3b).

Kinases play important roles in various cellular processes via signaling transduction, influencing the cellular proteome^34^. To unravel the dynamic alterations in both kinases and phosphoproteins, and the impact on the proteome in AMPAC, we conducted kinase-substrate enrichment analysis (KSEA). We detected ten kinases that were specifically activated in tumors, with five (CDK2, CDK1, CDK7, CDK4, and CDK6) of them belonging to the cyclin-dependent kinase (CDK) family (Supplementary Fig. S4g). Therefore, we screened for phosphorylation substrates of CDK1, CDK2, and CDK7 that exhibited high expression levels in the tumors (FC (T/N) > 1.5, *p* < 0.05) within Fudan cohort, identifying specific phosphorylation sites on some phosphoproteins (DNM2, HNRNPK, NPM1, PML, PPP1CA, RB1, and TCOF1) targeted by CDK1 as well as CDK2 (Supplementary Fig. S4h). Furthermore, it is noteworthy that all of these phosphorylation substrates exhibit interactions among themselves. Functionally, according to the STRING database, these interactions contribute to the impact on focal adhesion and the cell cycle in AMPAC tumors, providing partial insight into the enrichment of cell cycle at the protein level in tumors (Supplementary Fig. S4i). Moreover, survival analysis indicated a negative correlation between CDK2 kinase activity and patient overall survival (Supplementary Fig. S4j). The abundances of CDK2/CDK1 phosphorylation substrates, including DNM2/S764, HNRNPK/S216, NPM1/S70, PML/S518, and GIGYF2/S593, CDK7’s phosphorylation substrate CDK1/T161, exhibited significant negative correlations with overall survival (Supplementary Fig. S4k–p). In summary, we identified CDKs, especially CDK2, that play a crucial role in AMPAC tumorigenesis processes by promoting the cell cycle and focal adhesion pathway. Furthermore, it is hypothesized that CDK2 could be a potential druggable protein to improve AMPAC patients’ prognostic outcomes (Supplementary Fig. S4q).

### Intestinal-type features with PCNA amp and pancreatobiliary-type features with ANO1 amp

**Fig. 4:**
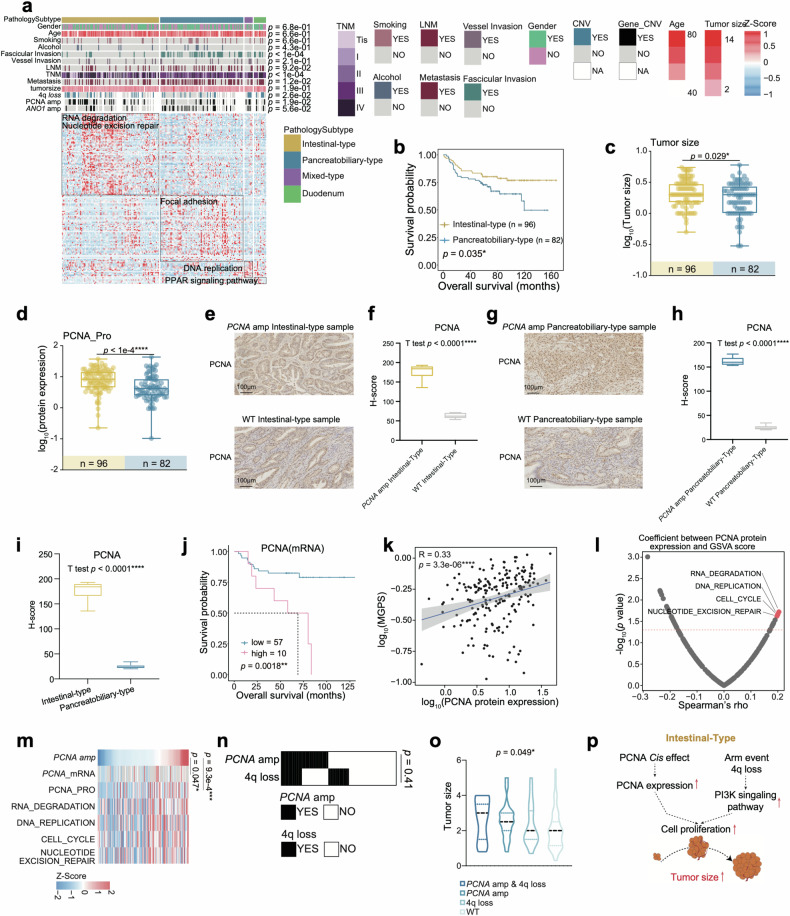
Intestinal-type features with PCNA amp. **a** Heatmap illustrated the characterization of four pathology subtypes. Each column represents a patient sample and rows indicate proteins. The color of each cell shows the *z*-score of the protein in that sample. AMPAC pathology classification, clinical features, and CNV status are shown above the heatmap. The *χ*^2^ test was used to evaluate the association of pathology subtypes with the variables on the heatmap, and *p* values were listed on the right. Single-sample Gene Set Enrichment Analysis (ssGSEA) based on proteomics data was also applied to identify the dominant pathway signatures in each pathology subtype. **b** The Kaplan–Meier curve for overall survival based on pathology subtype (log-rank test, *p* = 0.035). **c** *PCNA* amplification status in intestinal-type (left column, yellow) and pancreatobiliary-type (right column, blue) (Fisher’s exact test, *p* = 0.019). **d** Boxplot illustrated the tumor size among intestinal-type (yellow) and pancreatobiliary-type (blue) (Wilcoxon rank-sum test, *p* = 0.029). **e** IHC staining images exhibited the expression of PCNA between the intestinal-type samples with and without *PCNA* amp (Scale bars = 100 μm). **f** Boxplot exhibited the H-score of PCNA IHC images in the intestinal-type samples (Student’s *t* test, *p* < 0.0001). **g** IHC staining images exhibited the expression of PCNA between the pancreatobiliary-type samples with and without *PCNA* amp (Scale bars = 100 μm). **h** Boxplot exhibited the H-score of PCNA IHC images in the pancreatobiliary-type samples (Student’s *t* test, *p* < 0.0001). **i** Boxplot exhibited the H-score of PCNA IHC images in the intestinal-type and pancreatobiliary-type samples (Student’s *t* test, *p* < 0.0001). **j** The Kaplan–Meier curve for overall survival based on the PCNA mRNA expression (log-rank test *p* = 0.0018). **k** Spearman correlation of the abundance of PCNA and multi-gene proliferation scores (MGPS) (Spearman’s *r* = 0.33, *p* = 3.3e−6). **l** Spearman correlation of PCNA protein abundance and GSVA score (Spearman correlation). **m** Heatmap illustrated the *PCNA* amplification, the mRNA/protein abundance of PCNA, and GSVA scores. Spearman correlation tests were performed between *PCNA* amplification and the mRNA/protein abundance of PCNA, and *p* values were shown on the right. **n** The heatmap indicated the CNV status of *PCNA* amplification and 4q loss. Fisher’s exact test was used to evaluate the association between 4q loss and *PCNA* amplification, and the *p* value was on the right. **o** Violin plot indicated the comparisons of the four groups for the tumor sizes (Kruskal–Wallis test, *p* = 0.049). **p** Illustration of the regulatory role of *PCNA* amplification.

According to the AJCC eighth edition 2017 staging system^35^. AMPAC was classified into intestinal-type, pancreatobiliary-type, and mixed-type. Our cohort contained 12 DAC patients and 186 AMPAC patients, comprising 92 intestinal-type patients, 86 pancreatobiliary-type patients and 8 mixed-type patients. Regarding histologic grading, the pancreatobiliary-type, with a higher grade, low-grade differentiation, and high lymph node metastasis rate compared to the intestinal-type, had the poorest overall survival and progression-free survival (Fig. 4a, b and Supplementary Fig. S5a, b). Differential analysis was performed to reveal the molecular differences among the four subtypes. KEGG pathway enrichment analysis showed that the intestinal-type was involved in RNA degradation and nucleotide excision repair, while the pancreatobiliary-type was characterized by focal adhesion, the mixed-type mainly participated in DNA replication and DAC was enriched with the PPAR signaling pathway (Fig. 4a and Supplementary Table S3c).

In Fudan cohort, we observed larger tumor sizes in the intestinal-type than in the pancreatobiliary-type (Fig. 4c). The higher metastasis rate occurred in pancreatobiliary-type patients than in the intestinal-type patients (Supplementary Fig. S5e). To further investigate the instinct causes of these phenotypes in the two subtypes, we examined all mutations and *cis* effect genes accumulation across all subtypes. *PCNA* amplification (amp) displayed a more frequent occurrence in the intestinal-type than pancreatobiliary-type (Supplementary Fig. S5c), and *ANO1* amplification occurred more frequently in the pancreatobiliary-type compared to the intestinal-type (Supplementary Fig. S5g). The 4q loss group had a higher multi-gene proliferation scores (MGPS) score compared to WT group (Supplementary Fig. S3h). Here, we checked the correlation between 4q loss and histology. We found that 4q loss also occurred more frequently in the intestinal-type than in the pancreatobiliary-type (Supplementary Fig. S5d).

Based on the *cis* effect of *PCNA* amp, PCNA expression level was upregulated in the intestinal-type (Fig. 4d). As shown in Fig. 4e, g, it was evident that the expression level of PCNA was higher in intestinal-type patients compared to pancreatobiliary-type patients. The boxplot of H-Score derived from the IHC result also verified this finding (Fig. 4i). However, it was still unknown whether the high expression level of PCNA in the intestinal-type was due to the *PCNA* amp or the influence of the different morphology of the two histological subtypes. To address this question, we performed an IHC analysis to compare the PCNA expression abundance in the *PCNA* amp and WT samples from the pancreatobiliary-type and intestinal-type, respectively. As shown in Fig. 4e, g, we found that the intestinal-type samples with *PCNA* amp had a significantly higher expression abundance of PCNA than the intestinal-type samples without PCNA amp, and the boxplot of the H-score illustrated the same fact that PCNA expression was higher in the intestinal-type samples with *PCNA* amp than WT (Fig. 4e, f). Meanwhile, we found that the pancreatobiliary-type samples with PCNA amp also had a higher expression abundance of PCNA than the intestinal-type samples without PCNA amp, the comparison of H-score showed a higher PCNA expression in the pancreatobiliary-type samples with *PCNA* amp than WT (Fig. 4g, h). Therefore, the higher expression in the intestinal-type samples was indeed due to the *PCNA* amp.

Survival analysis indicated that higher PCNA expression led to a poorer prognosis (Fig. 4j). Spearman correlation analysis of PCNA and MGPS showed a significantly positive correlation (Spearman’s *r* = 0.36, *p* < 1e−4) (Fig. 4k). Next, the correlation of PCNA expression and GSVA score was employed to analyze the most significant pathway associated with PCNA (Fig. 4l, m); these pathways were characterized by RNA degradation, DNA replication, cell cycle and nucleotide excision repair. All the results above indicated that *PCNA* amplification promoted tumor cell proliferation in intestinal-type patients (Supplementary Table S3d).

Considering the *cis* effect of *HADH* in 4q loss would promote fatty acid accumulation and further induce cell proliferation described earlier. To explore the stacking effect of 4q loss and *PCNA* amplification, we performed the Fisher’s exact test between 4q loss and *PCNA* amp (Fisher’s exact test, *p* = 0.41) (Fig. 4n) and the results showed that there was no co-occurring or exclusive relationship between 4q loss and *PCNA* amp. We checked the tumor size differences in the *PCNA* amp & 4q loss group, 4q loss group, *PCNA* amp group, and WT group. The results showed that patients in the *PCNA* amp & 4q loss group had the largest tumor sizes. The patients in the 4q loss group had a larger average tumor size than the patients in the *PCNA* amp group, and the WT group had the smallest average tumor size (Kruskal–Wallis test, *p* = 0.029) (Fig. 4o). In conclusion, the *cis* effect of *PCNA* amp elevates PCNA protein expression, inducing cell proliferation and leading to an increase in tumor size in the intestinal-type. Meanwhile, this process is further aggravated due to frequent 4q loss in the intestinal-type. The *cis* effect of HADH decreases its protein abundance, causing an accumulation of fatty acids that promotes tumor growth via the PI3K-Akt signaling pathway (Fig. 4p).

**Fig. 5:**
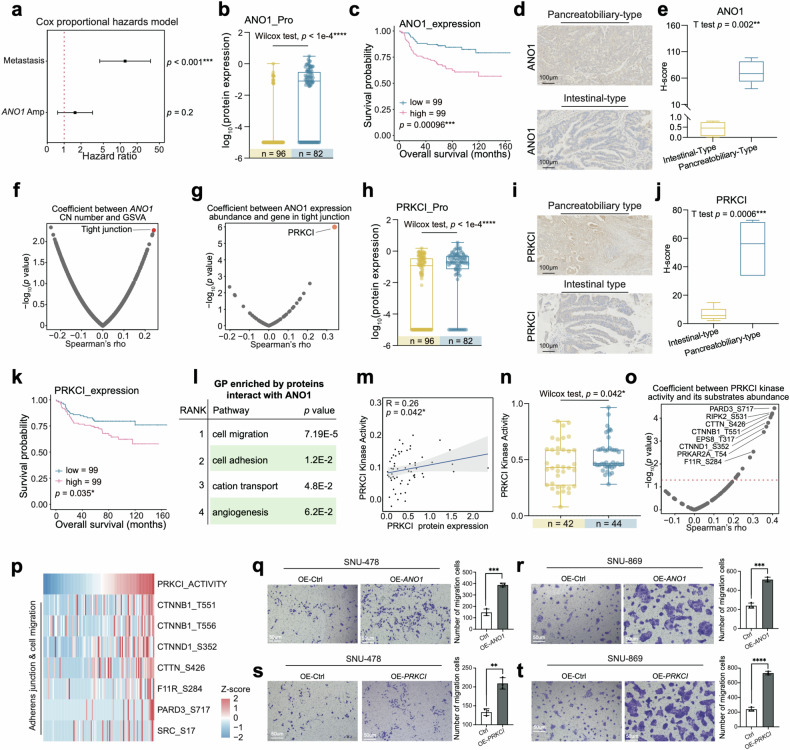
Pancreatobiliary-type features with ANO1 amp. **a** Metastasis status in intestinal-type (left column, yellow) and pancreatobiliary-type (right column, blue) (Fisher’s exact test, *p* = 0.0025). **b** The diagram illustrated the selection process of frequently occurred *cis* effect in the pancreatobiliary-type. **c** *ANO1* amplification status in intestinal-type (left column, yellow) and pancreatobiliary-type (right column, blue) (Fisher’s exact test, *p* = 0.018). **d** The forest plot indicated the hazard ratios of metastasis status and *ANO1* amplification. **e** Boxplot exhibited the H-score of ANO1 IHC images in the intestinal-type and pancreatobiliary-type samples (Student’s *t*-test, *p* < 0.0001). **f** Spearman correlation of ANO1 protein abundance and GSVA score (Spearman’s correlation). **g** Spearman correlation of ANO1 protein abundance and gene in tight junction pathway (Spearman’s correlation). **h** Boxplot illustrated PRKCI protein expression among intestinal-type (yellow) and pancreatobiliary-type (blue) (Wilcoxon rank-sum test, *p* < 1e−4). **i** IHC staining illustrated PRKCI was highly expressed in the pancreatobiliary-type sample compared to the intestinal-type sample (Scale bar = 100 μm). **j** Boxplot exhibited the H-score of PRKCI IHC images in the intestinal-type and pancreatobiliary-type samples (Student’s *t*-test, *p* < 0.0001). **k** The Kaplan–Meier curve for overall survival based on the ANO1 protein expression (log-rank test, *p* = 0.035). **l** GO pathways enriched by proteins interact with ANO1. **m** Spearman correlation of PRKCI protein abundance and PRKCI kinase activity (Spearman’s *r* = 0.26, *p* = 0.042). **n** Boxplot illustrated PRKCI kinase activity among intestinal-type (yellow) and pancreatobiliary-type (blue) (Wilcoxon rank-sum test, *p* = 0.042). **o** Spearman correlation of PRKCI kinase activity and the substrates of PRKCI (Spearman correlation). **p** Heatmap illustrated the PRKCI kinase activity and the abundance of substrates of PRKCI. **q** Transwell assay illustrated that OE-ANO1 groups in SUN-478 had enhanced migration ability compared with the control groups. The bar plots indicated the migrated cell counts of SNU-478 cells under different treatments (Student’s *t*-test). **r** Transwell assay illus*t*rated that OE-ANO1 groups in SUN-869 had enhanced migration ability compared with the control groups. The bar plots indicated the migrated cell counts of SNU-478 cells under different treatments (Student’s *t*-test). **s** Transwell assay illus*t*rated that OE-ANO1 groups in SUN-478 had enhanced migration ability compared with the control groups. The bar plots indicated the migrated cell counts of SNU-478 cells under different treatments (Student’s *t*-test). **t** Transwell assay illus*t*rated tha**t** OE-ANO1 groups in SUN-869 had enhanced migration ability compared with the control groups. The bar plots indicated the migrated cell counts of SNU-478 cells under different treatments (Student’s *t* test).

For pancreatobiliary-type patients, *ANO1* amplification was the only somatic copy number alteration (SCNA) that occurred more frequently in pancreatobiliary-type than intestinal-type patients (Supplementary Fig. S5f), and *ANO1* amp was a the risk factor for AMPAC (HR = 1.7, *p* = 0.2) (Fig. 5a). The *cis* effect of *ANO1* had an impact on its expression level, which was higher in the pancreatobiliary-type compared to intestinal-type (Fig. 5b). IHC staining illustrated ΑΝΟ1 high expression in the pancreatobiliary-type, and the H-Score quantified by IHC results also demonstrated the high expression of ANO1 in the pancreatobiliary-type samples (Fig. 5d, e). Survival analysis demonstrated that ANO1 expression level was negatively correlated with overall survival (Fig. 5c). As mentioned earlier, the pancreatobiliary-type was accompanied by a higher metastasis rate (Supplementary Fig. S5e). *ANO1* amp was also found to frequently occur in the metastasis group (Supplementary Fig. S5g), which also had a higher ANO1 protein expression (Supplementary Fig. S5h). The results above led us to investigate whether ANO1 amp was associated with the metastasis process in pancreatobiliary-type patients.

To further verify the hypothesis, the Spearman correlation was entitled to examine the correlation of *ANO1* copy number (CN) with GSVA score, and *ANO1* CN was found to be significantly associated with the GSVA score of tight junction (Fig. 5f and Supplementary Table S3e). The utilization of Spearman correlation analysis was entailed in *ANO1* copy number and proteins in the tight junction pathway, and the most significantly positive correlation with *ANO1* copy number among these genes was PRKCI (Fig. 5g), which encoded a member of the protein kinase C family of serine/threonine protein kinases. PRKCI was also found to be highly expressed in the pancreatobiliary-type and metastasis group, which was confirmed by IHC staining and the H-Score (Fig. 5h–j and Supplementary Fig. S5i). In addition, PRKCI protein abundance was negatively associated with overall prognosis (Fig. 5k). We investigated the relationship between the kinase activity of PRKCI and its expression abundance. As a result, the protein expression of PRKCI was positively correlated with its kinase activity (Spearman’s *r* = 0.26, *p* = 0.042) (Fig. 5m), and PRKCI kinase activity was also higher in pancreatobiliary-type (Fig. 5n). To further explore functional phosphor-substrates for PRKCI, we screened the referred kinase substrate pairs from database^36–38^ and executed correlation analysis. The result displayed that the most associated substrates were involved in adherens junction and cell migration, which are associated with metastasis processes (Fig. 5o, p), suggesting that the link between PRKCI protein expression upregulation and metastasis processes. Therefore, we hypothesized that the *cis* effect of *ANO1* elevated ANO1 protein abundance and interacted with PRKCI to regulate cell adhesion, thereby promoting metastasis in pancreatobiliary-type patients.

To verify the results mentioned above, we constructed ANO1 overexpressing and PRKCI overexpressing SNU-478 and SNU-869 cell lines (SNU-478-*ANO1*-OE, SNU-869-*ANO1*-OE, SNU-478-*PRKCI*-OE, SNU-869-*PRKCI*-OE), and transfected SNU-478 and SNU-869 with empty vector as a control (SNU-478-vector, SNU-869-vector). The relative mRNA level of *ANO1* and *PRKCI* was increased compared to the control (Supplementary Fig. S5j, k). We performed transwell migration assay to further evaluate the migration ability of ANO1 overexpression and PRKCI overexpression SNU-478 cells. As a result, transwell migration assay confirmed our findings and showed increased cell migration ability after ANO1 and PRKCI overexpressed in SNU-478 and SNU-869 cell lines (Fig. 5q–t), as shown in the barplots of the Fig. 5q–t, the ANO1 and PRKCI overexpression cell lines exhibited enhanced cell migration ability (Fig. 5q–t). These results all indicated that ANO1 high expression and PRKCI high expression could promote cell migration ability (Supplementary Fig. S5n). To further confirm the causal link between ANO1, PRKCI and metastasis, we conducted IP-MS to investigate ANO1-interacting proteins using anti-ANO1 antibody in both control group cells and *ANO1* overexpressing cells. Compared to the control group, we identified 34 proteins that specifically interacted with ANO1 in ANO1 overexpressing group cells (Supplementary Table S3f). GO enrichment analysis revealed the dominant pathways that were most significantly enriched by ANO1-interacting proteins were cell migration, cell adhesion, cation transport and angiogenesis (Fig. 5l and Supplementary Fig. S5l). The IP-MS results illustrated that ANO1 could interact with PRKCI in ANO1 overexpressing cells and showed a strong interaction with PRKCI (Supplementary Fig. S5m).

Besides, to verify the signaling pathways proposed in Fig. 4a, where the Mixed-type was primarily characterized by DNA replication and the Duodenum group was predominantly characterized by PPAR signaling pathway; we selected the key molecules MCM7 and POLA2 associated with the DNA replication and performed IHC staining in the Mixed-type. The IHC results showed that the expression levels of MCM7 and POLA2 in the Mixed-type were significantly higher than those in the other three pathological groups (Supplementary Fig. S6a, c). Additionally, we quantified the staining results by H-Score. We found that the H-Score of these two molecules was also significantly higher than that in the other three case groups (Supplementary Fig. S6b, d).

Similarly, we also validated the upregulation of the PPAR signaling pathway in the Duodenum pathological group by performing IHC staining on FABP1 and PCK1, which were the key molecules of PPAR signaling pathway. As shown in Supplementary Fig. S6e, f, it could be observed that the two molecules, which participated in PPAR signaling pathway, were highly expressed in the Duodenum pathological group. The H-score of PCK1 and FABP1 in the Duodenum pathological group was significantly higher than that in the other three pathological groups (Supplementary Fig. S6g, h).

In summary, two *cis* effects influenced their protein abundances to regulate the downstream signaling network, *PCNA* amp in the intestinal-type had a significant impact on cell proliferation, while *ANO1* amp in the pancreatobiliary-type had driven metastasis processes. The comprehensive proteogenomic analysis has presented the diversity between the intestinal-type and pancreatobiliary-type at both the genomic and proteomic levels. Additionally, IHC results validated the molecular features in the Mixed-type and Duodenum group patients, which were characterized by DNA replication and PPAR signaling pathway, respectively. These findings improved our understanding of the tumorigenesis mechanism related to the histological morphology and clinical characterization.

### Proteomic clusters of AMPAC patients

**Fig. 6:**
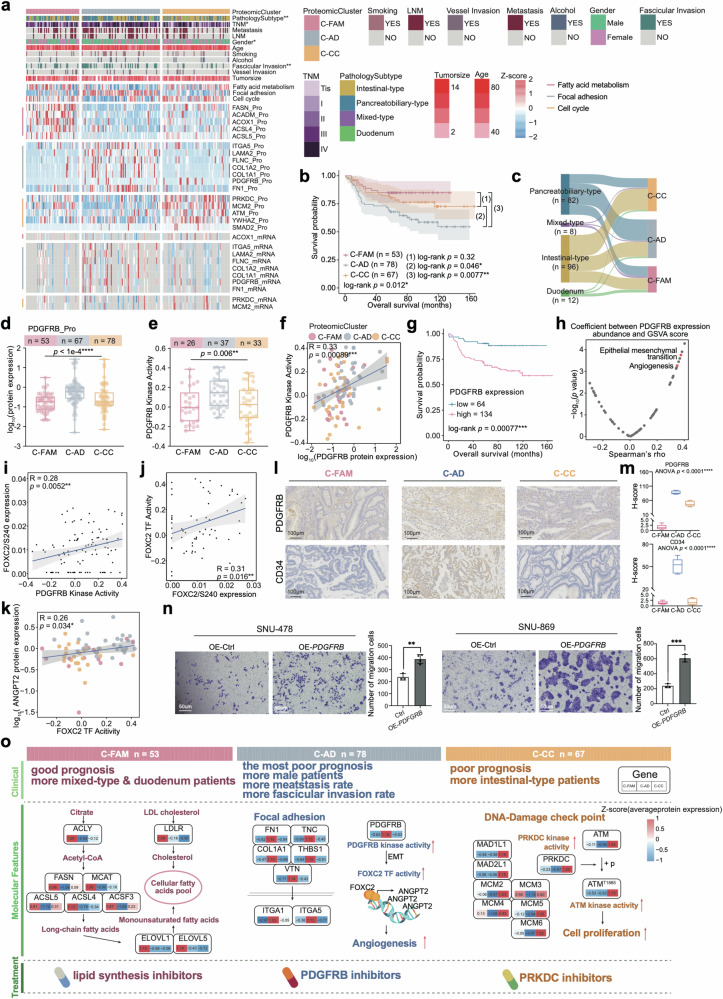
Proteomic subtypes of AMPAC patients. **a** Heatmap illustrated the characterization of three proteomic clusters. Each column represents a patient sample and rows indicate proteins or mRNAs. The color of each cell shows the *z*-score of the protein in that sample. AMPAC pathology classification, clinical features, and CNV status are shown above the heatmap. The *χ*^2^ test was used to evaluate the association of pathology clusters with the variables on the heatmap, and *p* values (****p < 1.0e−4, ***p < 1.0e−3, **p < 1.0e−2, *p < 0.05, n.s. > 0.05) were listed on the right. Single-sample Gene Set Enrichment Analysis (ssGSEA) based on proteomics data was also applied to identify the dominant pathway signatures in each proteomic cluster. **b** The Kaplan–Meier curve for overall survival based on proteomic clusters (log-rank test, *p* = 0.012 for comparison of 3 proteomic clusters, *p* = 0.32 for C-FAM and C-CC, *p* = 0.046 for the comparison of C-AD and C-CC, *p* = 0.0077 for the comparison of C-AD and C-FAM). **c** Sankey plot revealing the association between our proteomic clusters and pathology subtypes. **d** Boxplot illustrated PDGFRB protein expression among C-FAM (pink), C-AD (gray), and C-CC (yellow) (Kruskal–Wallis test, *p* < 1e−4). **e** Boxplot illustrated PDGFRB kinase activity among C-FAM (pink), C-AD (gray), and C-CC (yellow) (Kruskal–Wallis test, *p* = 6e−3). **f** Spearman correlation of PDGFRB protein expression and PDGFRB kinase activity (Spearman’s *r* = 0.33, *p* = 8.9e−4). **g** The Kaplan–Meier curve for overall survival based on the PDGFRB protein expression (log-rank test, *p* = 7.7e−4). **h** The scatter plot described the correlation between PDGFRB protein expression and the GSVA scores (Spearman’s correlation). **i** Spearman correlation of PDGFRB kinase activity and FOXC2/S240 abundance (Spearman’s *r* = 0.28, *p* = 5.2e−3). **j** Spearman correlation of the expression of FOXC2/S240 and the FOXC2 TF activity (Spearman’s *r* = 0.31, *p* = 0.016). **k** Spearman correlation of FOXC2 TF activity and ANGPT2 protein expression (Spearman’s *r* = 0.26, *p* = 0.034). **l** IHC staining illustrated that PDGFRB and CD34 we*r*e highly expressed in C-AD compared to C-FAM and C-CC cluster (Scale bars = 100 μm). **m** Boxplot exhibited the H-score of PDGFRB IHC images in the proteomic clusters (one-way ANOVA test, *p* < 0.0001). **n** Transwell assay illustrated that SNU-478-PDGFRB-OE group cells and SNU-869-PDGFRB-OE group cells had higher migration abilities compared to the control groups. The bar plots indicated the migrated cell counts of SNU-478 and SNU-869 cells under different treatments (Student’s *t*-test). **o** Schematic summary of the molecular characteristics of three proteomic clusters in C-FAM (left panel), C-AD (middle panel), and C-CC (right panel). Clinical features, molecular features, and potential treatment schedules for each proteomic cluster were listed on the top panel, middle panel, and bottom panel, separately.

The current pathologic staging system utilized for AMPAC is unable to accurately predict prognosis precisely or provide effective indications for treatment. Therefore, to systematically investigate the malignant cell heterogeneity of the pathologic subtype, consensus clustering was employed based on protein expression ranks in the tumor samples, and three clusters (C-FAM: *n* = 53; C-AD: *n* = 78; C-CC: *n* = 67) were identified among the 198 patients (Fig. 6a, Supplementary Fig. S7a and Table S4a). These three subgroups were found to be obviously different in overall survival (OS; log-rank test, *p* = 0.012, Fig. 6b). In addition, the distribution of histological subtypes varied among the proteomic clusters (*χ*^2^ test, *p* = 0.008) (Supplementary Fig. S8a and Fig. 6c).

The C-FAM cluster was characterized by the best prognosis; KEGG analysis illustrated that fatty acid metabolism was significantly enriched in C-FAM, and the crucial molecules involved in fatty acid metabolism, such as ACLY, FASN, ELOVL5, LDLR, and ACLS, showed high expression in the C-FAM cluster (Fig. 6a and Supplementary Fig. S9a). IHC staining further demonstrated that ACLY and FASN were highly expressed in the C-FAM cluster, and the H-Score qualified by IHC results illustrated that the expression of ACLY and FASN was higher in the C-CC cluster compared with the other two clusters (Supplementary Fig. S9b–d). Considering that 4q loss was identified as a CNV event related to fatty acid metabolism, we investigated its distribution in the C-FAM cluster and found that the C-FAM cluster had a lower proportion of 4q loss across three proteomic clusters (Supplementary Fig. S8h). These results indirectly validated that the *cis* effect on the 4q loss indeed regulated fatty acid metabolism. The C-FAM cluster harbored a lower incidence of 4q loss and was characterized by an upregulation of fatty acid metabolism.

The cluster C-AD was associated with the worst prognosis and was enriched in focal adhesion (Fig. 6a and Supplementary Fig. S8b). Additionally, the C-AD cluster displayed the largest proportion (55.1%) of pancreatobiliary-type samples (Supplementary Fig. S8a and Fig. 6c), the highest proportion of lymph node metastasis (LNM), the highest incidence rate of fascicular invasion, and the highest metastasis rate. The *cis* effect of *ANO1* amp could interact with PRKCI and promote the metastasis-associated processes. We observed that *ANO1* amp showed a slightly high level of enrichment in the C-AD cluster (Supplementary Fig. S8j). The mentioned above illustrated the heterogeneity of the tumor, and the role of *ANO1* amp in promoting metastasis.

The cluster C-CC had better overall survival than the C-AD cluster but poorer overall survival than the C-FAM cluster, which was characterized by cell cycle (Fig. 6a and Supplementary Fig. S9e). The cluster C-CC had the largest proportion (64.2%) of intestinal-type samples (Supplementary Fig. S8a and Fig. 6c). The intestinal-type samples were characterized by cell proliferation and influenced by *PCNA* amp and 4q loss. Consequently, we examined the distribution of *PCNA* amp and 4q loss, observing a greater accumulation of these two CNV events in the C-CC cluster (Supplementary Fig. S8h, i). Based on our genomic analysis results, genomic variations have an impact on the proteome, emphasizing the importance of multi-omics analyses for a deeply comprehensive understanding of tumor molecular mechanisms. Although these genomic variations associated with poor prognosis displayed a distinct distribution among the three clusters, there were no statistically significant differences in the distribution. We speculated that the distinction of proteins and phosphoproteins among the three clusters is crucial for the differences in prognostic outcome.

Therefore, we conducted differential analysis across the proteomic clusters at both the protein and phosphoprotein levels. PDGFRB protein expression level and RNA expression level were found to be the highest in the C-AD cluster and negatively correlated with overall survival (OS; log-rank test, *p* = 7.7e−4, Fig. 6d, g and Supplementary Table S4b, c). We found that PDGFRB kinase activity had a significantly positive correlation with its expression and was higher than that of the other two clusters (Fig. 6e, f). Spearman correlation analysis between PDGFRB kinase activity and GSVA score indicated that the most significant correlated pathways were epithelial mesenchymal transition (EMT) and angiogenesis (Fig. 6h). Then, we screened for PDGFRB substrates that were involved in EMT pathway, which had the highest expression in C-AD cluster. We identified a transcription factor (TF) FOXC2 (Supplementary Fig. S8c), phosphorylated by PDGFRB at Ser 240. The abundance of this phosphorylation site was positively correlated with PDGFRB kinase activity (Fig. 6i). FOXC2 is a TF belonging to the forkhead family that plays a crucial role in various processes, including angiogenesis, lymphangiogenesis, and adipogenesis^39^. We found FOXC2 TF activity was positively associated with FOXC2/S240 abundance (Fig. 6j), and also positively correlated with PDGFRB kinase activity (Supplementary Fig. S8e). These results illustrated the TF activity of FOXC2 was dominantly contributed by phosphor-FOXC2. In addition, FOXC2 TF activity was found to be negatively correlated with overall survival (OS; log-rank test, *p* = 2.1e−3, Supplementary Fig. S8d).

To gain insight into the mechanism of how FOXC2 TF activity impacted prognosis, we inferred the FOXC2 TF activity based on mRNA expression of its target genes (TGs) using the GSVA algorithm. ANGPT2 was the only TG of FOXC2 according to the database^40^, which was referred to as angiopoietin 2, affected angiogenesis and regulated endothelial cell adhesion, migration, and growth during tumorigenesis. The transcriptional regulatory pattern was inherited at the mRNA level, validated by a positive correlation between the ANGPT2 mRNA expression abundance and FOXC2 TF activity (Spearman’s *r* = 0.26, *p* = 0.034) (Fig. 6k)^41^. These findings above indicated that PDGFRB is highly expressed in the C-AD cluster, significantly influencing the downstream regulatory network associated with angiogenesis and EMT (Supplementary Fig. S8g).

To further verify PDGFRB impaction, we confirmed the high expression of PDGFRB in the C-AD cluster by IHC staining, and the angiogenesis marker CD34 was also verified highly expressed in C-AD cluster by IHC staining (Fig. 6l). Besides, the H-Score qualified by IHC staining results validated the high expression of PDGFRB and CD34 in the C-AD cluster compared with the other two clusters (Fig. 6m). We then transfected SNU-478 and SNU-869 cell lines with PDGFRB overexpressed plasmid to construct PDGFRB overexpression cells (SNU-478-*PDGFRB*-OE, SNU-869-*PDGFRB*-OE). RT-PCR analysis demonstrated that the *PDGFRB* mRNA level was significantly increased in PDGFRB overexpressing cells (Supplementary Fig. S8f). We then evaluated cell migration rates using transwell assay. As a result, the transwell migration assay verified our findings and validated increased cell migration ability after PDGFRB was overexpressed in SNU-478 and SNU-869 cell lines (Fig. 6n); the barplots in Fig. 6n also showed the enhanced cell migration ability in PDGFRB overexpressed cells (Fig. 6n).

Furthermore, we noted the highest levels of both PRKDC expression and kinase activity in C-CC (Supplementary Fig. S9f, g and Table S4b, c). IHC staining and the H-Score qualified by IHC result further confirmed elevated expression of PRKDC in the C-CC cluster (Supplementary Fig. S9h, i). We then investigated the relationship between the kinase activity of PRKDC and its expression abundance. As a result, the protein expression of PRKDC was positively correlated with its kinase activity (Spearman’s *r* = 0.21, *p* = 0.051) (Supplementary Fig. S9j). *PRKDC* encoded the catalytic subunit of the DNA-dependent protein kinase, we then investigated functional phosphor-substrates for PRKDC and referred kinase-substrates pairs from database^36–38^. These substrates involved in cell cycle showed a positive correlation with PRKDC kinase activity (Supplementary Fig. S9k). Notably, ATM/S1885 displayed a strongly positive correlation with PRKDC kinase activity (Supplementary Fig. S9l, m). *ATM*-encoded protein belongs to the PI3/PI4-kinase family, which is a crucial cell cycle checkpoint kinase that was phosphorylated by PRKDC in our data. Therefore, the C-CC cluster was correlated with cell cycle and might be accompanied by high PRKDC protein abundance and kinase activity, which led to downstream ATM phosphorylation, thereby promoting cell proliferation (Supplementary Fig. S9n).

Our findings not only reflected genomic alterations (including 4q loss, *PCNA* amp, *ANO1* amp) but also revealed the substantial role of protein and phosphoprotein profiles contribution to the proteomic cluster. We summarized the characteristics of three clusters and proposed potential therapeutic targets. The C-FAM cluster, characterized by fatty acid metabolism, can be potentially targeted using lipid synthesis inhibitors to suppress tumor development. Cluster C-AD was notable for its prominent angiogenesis signaling. In this context, inhibitors targeting PDGFRB could potentially be employed to suppress tumor progression in patients exhibiting characteristics of this cluster. The subtype characterized by the cell cycle can theoretically be targeted using PRKDC inhibitors to suppress tumor cell proliferation (Fig. 6o).

### Characterization of immune infiltration in AMPAC

**Fig. 7:**
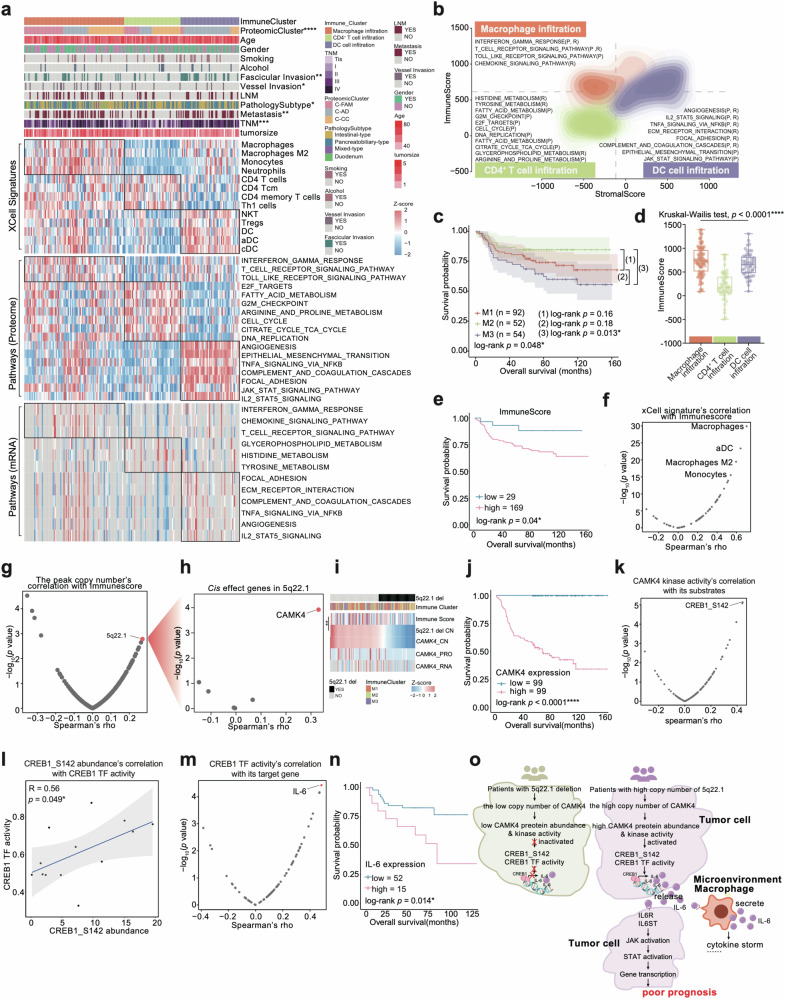
Characterization of immune infiltration in AMPAC. **a** Heatmap illustrated the characterization of three immune clusters. Each column represents a patient sample and rows indicate xCell signatures. The color of each cell shows the *z*-score of the xCell signature in that sample. AMPAC pathology classification, clinical features, and CNV status are shown above the heatmap. The *χ*^2^ test was used to evaluate the association of pathology subtypes with the variables on the heatmap, and *p* values (****p < 1.0e−4, ***p < 1.0e−3, **p < 1.0e−2, *p < 0.05, n.s. > 0.05) were listed on the right. Single-sample Gene Set Enrichment Analysis (ssGSEA) based on proteomic and transcriptomic data were also applied to identify the dominant pathway signatures in each immune cluster. **b** Contour plot of two-dimensional density based on immune scores (*y*-axis) and stromal scores (*x*-axis) for different immune clusters. For each immune cluster, key upregulated pathways and molecules were reported based on RNA-seq (R), and proteomics (P) in the annotation boxes. **c** Kaplan–Meier curves for overall survival based on immune clusters (log-rank test, *p* = 0.048 for the comparison of 3 immune clusters, *p* = 0.16 for the comparison of M1 and M2, *p* = 0.18 for the comparison of M2 and M3, *p* = 0.013 for the comparison of M2 and M3). **d** The boxplot indicated immune scores among the three immune clusters (Kruskal–Wallis test, *p* < 1e−4). **e** Kaplan–Meier curves for overall survival based on immune score (log-rank test, *p* = 0.04). **f** The scatter plot described the correlation between the immune score and the xCell signatures (Spearman correlation). **g** The scatter plot described the correlation between the immune score and all focal events copy number (Spearman correlation). **h** Volcano plot showing the *cis* effect genes on 5q22.1 (Spearman correlation). **i** The top panel was 5q22.1 deletion focal event distribution, and the medium panel was the immune cluster distribution; the bottom heatmap showed immune score, 5q22.1 copy number, CAMK4 copy number, CAMK4 protein expression abundance, and CAMK4 mRNA abundance. **j** Kaplan–Meier curves for overall survival based on CAMK4 protein expression (log-rank test, *p* < 1e−4). **k** The scatter plot described the correlation between the CAMK4 kinase activity and substrates of CAMK4 (Spearman correlation). **l** Spearman correlation of CREB1 TF activity and expression of CREB1/S142 (Spearman’s *r* = 0.56, *p* = 0.049). **m** The scatter plot described the correlation between the CAMK4 TF activity and target genes of CAMK4 (Spearman correlation). **n** Kaplan–Meier curves for overall survival based on IL-6 mRNA expression (log-rank test, *p* = 0.014). **o** The schematic diagram summarized that *CAMK4 cis* effect changed its downstream signaling, and led to alterations in the immune microenvironment, which were associated with a poorer prognosis for patients.

Immunotherapy has been applied to the treatment of several cancers. To explore the immune microenvironment characteristics of AMPAC, we utilized xCell analysis based on proteomic data to infer the relative abundance of different cell types in the tumor microenvironment^42^ (Fig. 7a and Supplementary Table S5a). Subsequently, consensus clustering based on inferred cell proportions identified three sets of tumors with noticeable clinical features and immune cell types: macrophage infiltration cluster (M1: *n* = 92), CD4^+^ T cell infiltration cluster (M2: *n* = 52) and DC cell infiltration cluster (M3: *n* = 54) (Fig. 7a, Supplementary Fig. S10a and Table S5b). Survival analysis displayed that the immune clusters significantly differed in overall survival (OS; log-rank test, *p* = 0.048), suggesting that different clusters of immune cell infiltration could lead to diverse prognostic outcomes. Among them, the CD4^+^ T cell infiltration cluster had the best prognostic outcome, and the DC cell infiltration cluster exhibited the worst prognostic outcome (Fig. 7c).

The macrophage infiltration cluster was characterized by macrophages, macrophage M2, monocytes, and neutrophils, and showed the highest immune score (Kruskal–Wallis test, *p* < 1e−4) (Fig. 7d and Supplementary Table S5c). Moreover, the macrophage infiltration cluster displayed upregulation of interferon γ response, T cell receptor signaling pathway, toll-like receptor signaling pathway, and chemokine signaling pathway (Kruskal–Wallis test, *p* < 0.05) (Fig. 7a, b). CD4^+^ T cell infiltration cluster showed the lowest immune score which was characterized by CD4^+^ T cell, CD4^+^ Tcm, and CD4^+^ memory T cell (Kruskal–Wallis test, *p* < 0.05) (Fig. 7d). GSVA analysis indicated an elevation in several pathways, including fatty acid metabolism, cell cycle, and tyrosine metabolism, within the CD4^+^ T cell infiltration cluster (Kruskal–Wallis test, *p* < 0.05) (Fig. 7a, b). The DC cell infiltration cluster predominantly displayed the infiltration of NKT, Tregs, DC, aDC, and cDC in deconvolution analyses (Fig. 7a, b). Compared to the CD4^+^ T cell infiltration cluster, the DC cell infiltration cluster had the higher immune score (Kruskal–Wallis test, *p* < 1e−4) (Fig. 7d). KEGG pathway enrichment analysis in the DC cell infiltration cluster revealed significant enrichment of the angiogenesis, epithelial mesenchymal transition, TNFα signaling via NFκB, complement and coagulation cascades, focal adhesion, JAK-STAT signaling pathway (Kruskal–Wallis test, *p* < 0.05) (Fig. 7a, b). The immune score was found to be varied among the three immune clusters (Fig. 7d), and survival analysis displayed a negative correlation between immune score and overall survival (OS; log-rank test, *p* = 0.04, Fig. 7e). Furthermore, it seemed that the differences in prognostic outcome among the three clusters could be linked to the distinct immune score in each immune cluster. This motivated us to explore the factors causing the negative correlation between immune score and overall survival.

The immune score was calculated by the sum of all immune and stromal cell types^42^. Therefore, before investigating the factors that cause the negative correlation between immune score and overall survival, it was necessary to examine which cell type predominantly contributes to the immune score. Spearman correlation analysis was utilized to check the relationship between cell type enrichment score and immune score. Macrophages, aDC, macrophage M2, and monocytes showed a significant association with immune score (Fig. 7f). Interestingly, the immune score was found to be negatively correlated with overall survival. CNVs were utilized to explore the internal mechanism of a high immune score linked to a poor prognosis.

To investigate what caused the high immune score featured with poor prognosis, Spearman correlation analysis was employed to explore the correlation of CNVs with the immune score. The copy number of 5q22.1 was found to be significantly positively correlated with the immune score (Spearman’s *r* = 0.27, *p* = 0.0016, Fig. 7g and Supplementary Table S5d). The copy number alteration, 5q22.1, was identified as a deletion event in the GISTIC analysis results in this study. Therefore, 5q22.1 deletion frequently occurred in the patients with a lower immune score. To investigate the potential mechanism by which 5q22.1 affected the immune score of AMPAC, genes with a significantly positive correlation between genes’ copy number and their mRNA or protein expression abundance located on 5q22.1 would be focused. CAMK4 was observed to have the most significantly positive correlation between its copy number and protein expression abundance (Spearman’s *r* = 0.33, *p* = 0.00012, Fig. 7h, i and Supplementary Table S2h). Since CAMK4 was located on 5q22.1, the copy number loss CAMK was induced by 5q22.1 deletion. Additionally, the copy number of CAMK4 showed a significant positive correlation with its expression abundance. In other words, 5q22.1 deletion is accompanied by a low CAMK4 copy number, resulting in a low expression abundance of CAMK4 (Fig. 7i). Therefore, the patients with a high immune score had a higher copy number of 5q22.1 and a higher copy number and expression abundance of CAMK4 (Fig. 7i). CAMK4 encoded the protein belonging to the serine/threonine protein kinase family and showed a negative correlation with overall prognosis in our study (OS; log-rank test, *p* < 1e−4, Fig. 7j). The kinase activity of CAMK4 was positively associated with its protein expression abundance (Spearman’s *r* = 0.32, *p* = 0.0015). This suggested that the high copy number of CAMK4 elevated its protein expression and upregulated its kinase activity. To explore the downstream signaling network of CAMK4, we conducted a correlation analysis between the substrate expression abundance of CAMK4 and CAMK4 kinase activity by using Spearman’s correlation test. CAMK4 kinase activity showed the most significant elevation of the CREB1/S142 phosphorylation (Fig. 7k, Spearman’s *r* = 0.44, *p* < 1e−4). CREB1 is a TF that regulates multiple signaling pathways^43^. Then, we inferred the CREB1 TF activity based on the mRNA expression of its target genes (TGs) using the GSVA algorithm. The inferred TF activity of CREB1 displayed high correlations with the abundance of CREB1/S142 (Fig. 7l, Spearman’s *r* = 0.56, *p* = 0.049). All of the TGs of CREB1, IL-6 showed the highest correlation with CREB1 TF activity (Fig. 7m, Spearman’s *r* = 0.56, *p* = 0.049) and negative correlation with overall prognosis at mRNA level (Fig. 7n).

IL-6 is a proinflammatory cytokine that plays a crucial role in cancer progression and high levels of IL-6 are associated with promoting tumorigenesis, invasiveness, and metastasis in various types of cancer^44^. Notably, we observed a significant elevation in the expression levels of IL-6 (Kruskal–Wallis test, *p* = 0.01) in macrophage infiltration cluster and DC cell infiltration cluster compared to CD4^+^ T cell infiltration cluster (Supplementary Fig. S11a). This was further confirmed by IHC staining and H-Score (Supplementary Fig. S11h, i). IL-6 signaling transduction requires interaction with its receptor. Thus, we conducted a comprehensive analysis of gene expression profiles, focusing on IL-6, IL6ST, and IL6R, across three distinct immune clusters, and calculated the Spearman correlation between IL-6 and IL6ST. Similarly, IL6ST and IL6R expression exhibited a corresponding increase with IL-6 in Macrophage infiltration cluster and DC cell infiltration cluster compared to CD4^+^ T cell infiltration cluster (IL6ST: Kruskal–Wallis test, *p* = 0.03), and IL-6 showed positively correlated with IL6ST (Spearman’s *r* = 0.3, *p* = 0.013) (Supplementary Fig. S11a, c). We postulated that the IL-6 identified in our data might interact with IL6ST expressed in tumor cells, immune and stromal cells. The secreted IL-6 may bind to IL-6 receptor expressed in immune cells, thereby promoting the release of IL-6 from these immune cells, potentially leading to the occurrence of a cytokine storm^45^. Furthermore, the interaction could trigger downstream activation of IL-6/JAK/STAT, facilitating transcription and translation processes, thus promoting downstream diverse biological processes.

The findings above encouraged us to investigate the synergistic association between IL-6 and IL-6/JAK/STAT signaling pathway. Before this, we checked the IL-6/JAK/STAT GSVA score across three distinct immune clusters. We observed a substantial upregulation of the IL-6/JAK/STAT3 signaling pathway, evident at both protein (Kruskal–Wallis test, *p* < 1e−4) and mRNA levels (Kruskal–Wallis test, *p* = 0.002) within macrophage infiltration cluster and DC cell infiltration cluster, in contrast to CD4^+^ T cell infiltration cluster (Supplementary Fig. S11b). Then, we investigated the correlation between the abundance of IL-6 and IL-6/JAK/STAT GSVA score. The Spearman correlation test was utilized to examine the correlations. The results displayed that IL-6 abundance was positively associated with the GSVA score of the IL-6/JAK/STAT signaling pathway (Spearman’s *r* = 0.26, *p* = 0.038) (Supplementary Fig. S11c, d). The positive correlation between IL-6 and the GSVA score of the IL-6/JAK/STAT signaling pathway indicated that the upregulation of IL-6 indeed increased the IL-6/JAK/STAT signaling pathway in this study. Furthermore, we checked the relationship between the expression levels of genes involved in IL-6/JAK/STAT (JAK1, JAK3, STAT1, STAT3) and IL-6. The expression levels of both JAK1 (Spearman’s *r* = 0.29, *p* = 0.017) and JAK3 (Spearman’s *r* = 0.37, *p* = 0.0023) showed a significant positive correlation with IL-6 (Supplementary Fig. S11e). The correlation of STAT1 (Spearman’s *r* = 0.25, *p* = 0.039), STAT3 (Spearman’s *r* = 0.37, *p* = 0.0021) and IL-6 has also exhibited a positive association (Supplementary Fig. S11f). We confirmed the high expression of STAT1 in macrophage infiltration cluster and DC cell infiltration cluster compared to CD4^+^ T cell infiltration cluster by IHC staining and H-Score (Supplementary Fig. S11h, j). In addition, we observed a positive correlation between the transcriptional activity of both STAT1 (Spearman’s *r* = 0.41, *p* = 0.00055) and STAT3 (Spearman’s *r* = 0.47, *p* < 1e−4) and the expression of IL-6 (Supplementary Fig. S11g). These findings suggested that IL-6, through its interaction with its receptor, activated the IL-6/JAK/STAT signaling pathway, thereby promoting downstream transcription associated processes.

Collectively, our findings demonstrated a significantly positive correlation between the immune score and the copy number of 5q22.1. Therefore, in patients with low immune score, the copy number of 5q22.1 was low. These individuals carried 5q22.1 deletion, resulting in a low copy number of *CAMK4*, a gene located on 5q22.1. Consequently, the protein expression level of CAMK4 was also low, leading to reduced kinase activity of CAMK4. This reduction prevented the phosphorylation of the CREB1 and subsequent transcriptional activation of IL-6, contributing to the downregulation of IL-6/JAK/STAT signaling pathway, which led to a better prognosis. In contrast, the patients without 5q22.1 deletion suffered the upregulated IL-6/JAK/STAT signaling pathway resulting from high CREB1 transcriptional activity, which led to a poor prognosis (Fig. 7o).

## Discussion

AMPAC is a rare malignant neoplasm with an incidence of around 4–7 cases per 1,000,000 individuals. To date, only a limited number of genomic cohort studies exist, and a comprehensive multi-omics study has yet to be performed. This work represented a proteogenomic comprehensive analysis performed for 186 AMPAC patients and 12 DAC patients of the Fudan cohort. Proteomics, phosphoproteomics, whole-exome sequencing, and RNA sequencing data were generated as resources from this cohort. The previous studies identified a series of significantly mutated genes, *TP53*, *KRAS*, *APC*, *SMAD4*, *ARID2*, *CTNNB1*, and *ELF3*^11,12^. In Fudan cohort, *KRAS*, *TP53*, *APC*, *ARID2*, *SMAD4*, *CTTNB1*, *ATM*, *ELF3*, *PBRM1*, *CTTNB1* was also significantly identified. Consistent with previous studies, we identified focal events such as 9p21.3 deletion, 18q21.2 deletion, 8q24.21 amplification, 12q15 amplification, and 3q26.2 amplification. Arm-level events, such as 1q gain, 1p loss and 8p loss, have been consistently identified in both our data and previous studies. In addition, we have also observed additional CNVs such as 4q loss, 5q22.1 deletion in our data.

Compared to previous work, we identified a chromosome arm event, 4q loss, which was negatively correlated with overall prognosis. The most significant *cis* effect on 4q was hydroxyacyl-CoA dehydrogenase (HADH), which participated in fatty acid metabolism. Due to the *cis* effect, HADH downregulation decreased the fatty acid metabolism in our cohort and induced the accumulation of fatty acids. Dysregulation of fatty acid metabolism may lead to excessive accumulation of fatty acids in cancer cells, promoting uncontrolled cell proliferation and the development of cancer^46^, and a previous study illustrated that HADH downregulation promotes cell proliferation in tumor cells via PI3K-Akt signaling pathway^33^. In our data, we observed rapid tumor growth in mice treated with subcutaneous injection of HADH siRNA SNU-478 cells. This metabolism disruption was supposed to be linked to the metabolic therapy, ketogenic diet. The ketogenic diet (KD) containing high-fat, moderate-to-low-protein, and low-carbohydrate levels, which may target cancer cell metabolism, has the potential to impact tumor treatment and prognosis^47^. The KD had been discovered to possess an anti-tumor effect as a therapeutic intervention in cancer^48^, encompassing various types of tumors, such as colon and rectal cancer (CRC)^49^, pancreatic adenocarcinoma (PDAC)^50^ and hepatocellular carcinoma (HCC)^51^. However, the KD has also been reported to promote prostate cancer^52^ tumorigenesis and play a role in glioma recurrence^53^. The intestinal-type and pancreaticobiliary-type of AMPAC exhibit similarities with both colorectal cancer and pancreatic cancer^54^. Our study revealed that the accumulation of fatty acids, resulting from metabolic disruptions, may lead to a glucose dependency in tumor cells. These findings suggest that AMPAC patients might benefit from a ketogenic diet.

At the histological level, intestinal-type and pancreatobiliary-type belong to the category of AMPAC, which harbored distinct prognostic outcomes and molecular characteristics. Our study built upon previous research by incorporating genomic, proteomic and phosphoproteomic data, provides a deeper understanding of the mechanisms underlying the tumorigenesis of different AMPAC histological subtypes. We found that the intestinal-type featured cell proliferation and the pancreatobiliary-type featured a higher metastasis rate, which were induced by *PCNA* amp and *ANO1* amp, respectively. *PCNA* amp elevated PCNA expression level and promoted cell proliferation. *ANO1* amp induced ANO1 high expression, which interacted with PRKCI and facilitated the elevation of adherens junction and cell migration. This study revealed the important role in the tumorigenesis of PCNA in intestinal-type, and ANO1 in pancreatobiliary-type tumorigenesis. The results raised therapeutic targets for AMPAC. Specifically, the PCNA inhibitors (such as AOH1996^55^, T2AA^56^) are suggested for the intestinal-type to suppress cell proliferation, while the ANO1 inhibitors (such as benzbromarone^57^, Cepharanthine^58^, Ani9^59^) are recommended for the pancreatobiliary-type to attenuate the metastasis process.

Currently, there is no specific targeted therapy for AMPAC. Treatment regimens tend to favor gemcitabine for the pancreaticobiliary-type and fluorouracil for the intestinal-type, but there is no significant difference in chemotherapy effect within the two histological subtypes^60,61^. Nevertheless, due to the inherent subjectivity of histological classification and the limited specificity and sensitivity of these drugs for AMPAC, both therapies yielded poor responses. Thus, based on the current histological classification, our research presented the first proteomic classification of AMPAC, and the three proteomic clusters had distinct molecular features associated with the prognostic outcome and pathological characteristics. These three clusters effectively stratify the intestinal-type and pancreatobiliary-type into more aggressive pancreatobiliary-type, more aggressive intestinal-type, and better-prognosis pancreatobiliary-type and intestinal-type. The phosphoproteome was applied to analyze the kinase features of the proteomic cluster. The results showed that PDGFRB was activated in C-AD characterized by focal adhesion, PRKDC was activated in C-CC featured with cell cycle, the rest of the three clusters, and C-FAM displayed fatty acid metabolism upregulation. These observations suggest that PDGFRB inhibitor has the potential to be considered as a therapeutic drug for C-AD, and PRKDC inhibitor as a potential drug target for C-CC, the inhibitor to block fatty acid metabolism for C-FAM. This work demonstrated the impact of genomic alterations on the proteomic cluster and unveiled the crucial roles played by protein and phosphoprotein profiles in the determination of the proteomic cluster. Furthermore, our classification provides a resource for exploring biomarker candidates or potentially therapeutic targets in the future.

XCell scoring based on the proteomic data delineated distinct immune subtypes characterized by varying immune cell infiltration with distinctive molecular features. The M1 cluster and M3 cluster with poor prognosis exhibited a higher immune score compared to the M2 cluster. These features have been suggested to be induced by the significant correlation between the copy number of CAMK4 and its protein expression. CAMK4 functioned by phosphorylated serine 142 of CREB1 to elevate CREB1 transcription factor activity, and further promote IL-6 secretion. IL-6 is a proinflammatory cytokine that plays a complex role in cancer progression. Previous research has reported that high levels of IL-6 are associated with promoting tumorigenesis, invasiveness, and metastasis in various types of cancer, such as gastric cancer, pancreatic cancer, colorectal cancer, lung cancer, and bile duct cancer^44,62^, with its substantial involvement in driving tumor progression via the IL-6/JAK/STAT pathway. We observed substantial IL-6 secretion in AMPAC, supporting the reasonable hypothesis that IL-6 could serve as a potential immunotherapy target for AMPAC. The abundance of IL-6 was negatively correlated with overall prognosis in our data, and it was found to activate the IL-6/JAK/STAT pathway. Consequently, the treatment targeting IL-6 has the potential to prolong the overall survival for patients in AMPAC.

In this research, we must acknowledge that there were indeed common flaw of multi-omics studies in our work. It underplayed the variability in response to the same genomic drivers among different groups. This was shown by box plots that have significant differences in mean abundance values but overlapping expression ranges. This could be caused by the heterogeneity among tumor samples. The similar phenomenon also occurred in some multi-omics cancer research^17,63^; the molecules reported in these studies were validated not only at the IHC level but also with further functional experiments, potentially leading to their development as drug targets or biomarkers. These illustrated that the overlapping range of expression of differentially expressed proteins was generally observed in multi-omics cancer studies. Nevertheless, this was still a point that must be acknowledged and noted, requiring careful verification and validation after proposing such biomarkers or drug targets. Additionally, The partial Spearman correlation in this study was not very strong, possibly due to the cohort size and other clinical factors. Statistical significance was used to assess biological process inferences, but outliers and extreme values might affect the results. Removing extreme values and verifying processes through cell line assays could improve reliability.

In this study, the pancreatobiliary-type ampullary adenocarcinoma cell line SNU-478 and the intestinal-type ampullary adenocarcinoma cell line SNU-869 were used to functionally validate potential molecular targets identified through multi-omics data analysis. Despite using two cell lines, the number of cell lines available for functional validation remains very limited compared to the multiple cell lines available for other tumors. This limitation is related to the rarity of ampullary adenocarcinoma, as there are currently few recognized primary cell lines for ampullary adenocarcinoma, which are also difficult to obtain. Nevertheless, the limitation of the small number of AMPAC cell lines employed for functional validation should be acknowledged.

In summary, this multi-omics research facilitated the identification of the disruptions in fatty acid metabolism in AMPAC, attributed to the *cis* effect of 4q loss. The work offered insight into the mechanisms driving tumorigenesis in the two histological subtypes of AMPAC, and presented potential therapeutic targets based on CNV events. Furthermore, we performed the proteomic classification and immune clustering for AMPAC, and raised corresponding theoretical drug targets based on clusters. Overall, our study provides a comprehensive data resource which helps elucidate the functional mechanisms of genomic alterations that impact survival, treatment and other factors affecting the patient outcome and quality of life.

## Materials and methods

### Sample selection

The 186 ampullary adenocarcinoma and 12 duodenum adenocarcinoma FFPE tissues and paired NATs were acquired from Zhongshan Hospital, Fudan University from 2008 to 2017. All cases were collected regardless of histologic grade or surgical stage. Clinical information of these patients, including gender, age, drinking status, smoke status, vessel invasion, fascicular invasion, histological subtypes, TNM stages (AJCC cancer staging system 8th edition), cancer metastasis status, survival status, is listed in Supplementary Table S1a. All the patients received primary resection without any anti-cancer treatments prior to surgery. Postoperative surveillance and treatment were conducted consistently according to Zhongshan Hospital’s guidelines. Each sample was assigned a new research ID, and the patient’s name or medical record number used during hospitalization was deidentified.

### Sample preparation

FFPE specimens were prepared and provided by Zhongshan Hospital. One 3-μm-thick slide from FFPE blocks was sectioned for hematoxylin and eosin (H&E) staining. For genomic, proteomic, and phosphoproteomic sample preparation, 10-μm-thick slides were sectioned, deparaffinized with xylene and washed with gradient ethanol. Samples were just sectioned by 10-μm-thick slides without xylene deparaffinization nor gradient ethanol wash for RNA sample preparation. Selected specimens according to H&E staining were scraped, and materials were aliquoted and kept in storage at −80 °C until further processing.

### Pathology review

All samples were systematically evaluated to confirm the histopathological diagnosis and any variant histology according to the AJCC eighth edition 2017 staging system^35^ by three expert pathologists. Additionally, all tumor samples were assessed for tumor content, the presence and extent of tumor necrosis, and signs of invasion into the muscularis propria. The samples used for multi-omics analysis were characterized with histologic tumor purity ranged from 70% to 90%. Tumor samples were also evaluated for the presence and extent of inflammatory infiltrates, as well as for the type of the infiltrating cells (lymphocytes, neutrophils, eosinophils, histiocytes, plasma cells) in the tumor microenvironment. Any non-concordant diagnoses among the three pathologists were re-reviewed, and a resolution was reached following discussion.

The ABSOLUTE algorithm was utilized to evaluate the overall computational purity score for each sample. Computational tumor purity was inferred by R package ABSOLUTE^64^ using WES data, respectively. The detailed was provided in Supplementary Table S1c. Tumor purity among 4 pathological groups, proteomic clusters and Immune clusters were shown in Supplementary Figs. S1k, l, S8k and S11k, respectively.

### DNA extraction

For the WES analysis, DNA from 133 FFPE specimens of AMPAC was extracted according to the manufacturer’s instructions (QIAamp DNA Mini Kit; QIAGEN, Hilden, Germany). The isolated DNA quality and contamination were verified using the following methods:

1. DNA degradation and contamination were monitored on 1% agarose gels.
2. DNA concentration was measured via Qubit® DNA Assay Kit in Qubit® 2.0 Fluorometer (Invitrogen, CA, USA).

### Library preparation

A total quantity of 0.6 μg genomic DNA per sample was used as the input material for DNA preparation. Sequencing libraries were generated using Agilent SureSelect Human All Exon Kit (Agilent Technologies, CA, USA) following the manufacturer’s recommendations; further, index codes were added to each sample. Briefly, fragmentation was carried out by a hydrodynamic shearing system (Covaris, Massachusetts, USA) to generate 180–280- bp fragments. The remaining overhangs were converted into blunt ends via exonuclease/polymerase activity. Adapter oligonucleotides were ligated after adenylation of the 3′-ends of the DNA fragments. DNA fragments with ligated adapter molecules on both ends were selectively enriched via a polymerase chain reaction (PCR). Thereafter, libraries were hybridized with the liquid phase of biotin-labeled probes, and magnetic beads with streptomycin were used to capture the exons of genes. Captured libraries were enriched in another PCR reaction to add index tags to prepare them for sequencing. Finally, the products were purified using AMPure XP system (Beckman Coulter, Beverly, USA) and quantified using an Agilent high sensitivity DNA assay (Agilent) on an Agilent Bioanalyzer 2100 system (Agilent Technologies, CA, USA).

### Clustering and DNA sequencing

The clustering of the index-coded samples was performed on a cBot Cluster Generation System using a HiSeq PE Cluster Kit (Illumina) according to the manufacturer’s instructions. After cluster generation, the DNA libraries were sequenced on Illumina NovaSeq 6000 platform, and 150 bp paired-end reads were generated.

### WES quality control

The original fluorescence image files obtained from Novaseq platform are transformed into short reads (Raw data) by base calling and these short reads are recorded in FASTQ format, which contains sequence information and corresponding sequencing quality information. Sequence artifacts, including reads containing adapter contamination, low-quality nucleotides, and unrecognizable nucleotides^65^, undoubtedly set the barrier for the subsequent reliable bioinformatics analysis. Hence quality control is an essential step applied to guarantee meaningful downstream analysis. The steps of data processing were as follows:

1. Discard the paired reads if either one read contains adapter contamination (> 10 nucleotides aligned to the adapter, allowing ≤ 10% mismatch).
2. Discard the paired reads if more than 10% of bases are uncertain in either one read.
3. Discard the paired reads if the proportion of low quality (Phred quality < 5) bases is over 50% in either one read.

All the downstream bioinformatics analyses were based on the high-quality clean data, which were retained after these steps. At the same time, QC statistics including total reads number, raw data, raw depth, sequencing error rate, percentage of reads with Q30 (the percent of bases with phred-scaled quality scores > 30), and GC content distribution were calculated and summarized. WES was conducted with mean coverage depths of 108X for tumor samples and 118X for adjacent non-tumor brain samples, which is consistent with the recommendations for WES.

### Read mapping to reference sequence

Valid sequencing data were mapped to the reference human genome (UCSC hg19) using Burrows–Wheeler aligner (BWA) software to obtain the original mapping results stored in BAM format^66^. If one read, or one paired read, was mapped to multiple positions, the strategy adopted by the BWA was to choose the most likely placement. If two or more most likely placements were present, the BWA picked one randomly. Then, SAMtools^67^ and Picard (http://broadinstitute.github.io/picard/) were used to sort BAM files and perform duplicate marking, local realignment, and base quality recalibration to generate final BAM files for computation of the sequence coverage and depth. The mapping step was very difficult due to mismatches, including true mutations and sequencing errors, and duplicates resulting from PCR amplification. These duplicate reads were uninformative and should not be considered as evidence for variants. We used Picard to mark these duplicates for the follow-up analysis.

### Variant calling

Samtools mpileup and bcftools were used to perform variant calling and identify SNPs and InDels. Somatic SNP variant calls were assessed using MuTect^68^, and the Indels variant calls were assessed using Strelka^69^ with default options. The resulting somatic mutations were annotated using the ANOVAR RefSeq gene-based annotation.

### Copy number analysis

CNVs were called by following the somatic CNV calling pipeline in GATK’s (GATK 4) Best Practice. The results of this pipeline and segment files of every 1000 were input in GISTIC2^70^, to identify significantly amplified or deleted focal-level and arm-level events, with a *q* value < 0.1 considered significant. A log2 ratio cutoff 1 was used to define SCNA amplification and deletion. We further summarize the arm-level copy number change based on a weighted sum approach^15^, in which the segment-level log2 copy ratios for all the segments located in the given arm were added up with the length of each segment being weighted. To exclude false positives as much as possible, relatively stringent cutoff thresholds were used with the following parameters: -ta 0.1 -tb 0.1 -brlen 0.98 -conf 0.9. Other parameters were the same as default values.

### Co‑occurrence and mutual exclusivity analysis of mutations

Co-occurrence and mutually exclusive mutated genes were detected using Fisher’s exact test in order to determine the co-occurrence and mutually exclusively of significantly mutated genes in our mutational dataset.

### Analysis of significantly mutated genes

Filtered mutations (including SNV and indel) were further used to identify significantly mutated genes by MutSigCV (https://software.broadinstitute.org/cancer/cga/mutsig, version 1.41) with default parameters. Final MutSigCV *p* values were converted to *q* values using the method of Benjamini and Hochberg^71^, and genes with *q* ≤ 0.1 were declared to be significantly mutated.

### Mutation frequency in the Fudan cohort and previous AMPAC studies

Mutation frequencies for previous AMPAC studies were obtained from their supplementary materials^11,12^. The frequencies of all genes were compared with those from the Fudan cohort using Fisher’s exact test.

### Mutational signature analysis using the sigminer approach

Mutation signatures were jointly inferred for 133 tumors using the R package sigminer^72^. The sigminer approach (https://github.com/ShixiangWang/sigminer) was used to extract the underlying mutational signatures. The 96 mutation vectors (or contexts) generated by somatic SNVs based on six base substitutions (C > A, C > G, C > T, T > A, T > C, and T > G) within 16 possible combinations of neighboring bases for each substitution were used as input data to infer their contributions to the observed mutations. Sigminer using a non-negative matrix factorization (NMF) approach was applied to decipher the 96 × 133 (i.e., mutational context-by-sample) matrix for the 30 known COSMIC cancer signatures (https://cancer.sanger.ac.uk/cosmic/signatures) and infer their exposure contributions.

### Tumor mutational burden

Tumor mutational burden (TMB) was defined as the number of somatic mutations (including base substitutions and indels) in the coding region. Synonymous alterations were also counted^73^. To calculate the TMB, the total number of mutations counted was divided by the size of the coding sequence region of the Agilent SureSelect Human All Exon V6.

### RNA extraction

RNA was extracted from tissues by using TIANGEN® RNAprep Pure FFPE Kit (#DP439) according to the reagent protocols. For library preparation of RNA sequencing, a total amount of 500 ng RNA per sample was used as the input material for the RNA sample preparations. Sequencing libraries were generated using Ribo-off® rRNA Depletion Kit (H/M/R) (Vazyme #N406) and VAHTS® Universal V6 RNA-seq Library Prep Kit for Illumina (#N401-NR604) following the manufacturer’s recommendations, and index codes were added to attribute sequences to each sample. The libraries were sequenced on an Illumina platform and 150 bp paired-end reads were generated.

### RNA‑seq data analysis

RNA-seq raw data quality was assessed using FastQC (v0.11.9), and the adapter was trimmed with Trim_ Galore (version 0.6.6) before any data filtering criteria were applied. Reads were mapped onto the human reference genome (UCSC hg19) using STAR software (v2.7.7a). The mapped reads were assembled into transcripts or genes by using StringTie software (v2.1.4) and the genome annotation file (version hg19). For quantification purpose, the relative abundance of the transcript/gene was measured using the normalized metrics, fragments per kilobase of transcript per million mapped reads (FPKM). Transcripts with an FPKM score above 1 were retained, resulting in a total of 19,193 gene IDs. All known exons in the annotated files were 100% covered.

### Protein extraction and tryptic digestion

Lysis buffer (0.1 M Tris-HCl (pH 8.0), 0.1 M DTT (Sigma, 43,815), 1 mM PMSF (Amresco, M145)) was added to the extracted tissues and subsequently sonicated for 1 min (3 s on and 3 s off, amplitude 25%) on ice. The supernatants were collected, and the extracted tissues were then lysed with 4% sodium dodecyl sulfate (SDS) and kept for 2 h at 99 °C with shaking at 1500 rpm. The solution was collected by centrifugation at 12,000× *g* for 5 min. A fourfold volume of acetone was added to the supernatant and kept in –20 °C for a minimum of 4 h. Subsequently, the acetone-precipitated proteins were washed three times with cooled acetone and then pumped out using the Concentrator plus (Eppendorf, Germany). Filter-aided sample preparation (FASP) procedure was used for protein digestion^74^. The proteins were resuspended in 200 μL 8 M urea (pH 8.0) and loaded twice in 30 kD Microcon filter tubes (Sartorius) and centrifuged at 12,000× *g* for 20 min. The precipitate in the filter was washed twice by adding 200 μL 50 mM NH_4_HCO_3_. The precipitate was resuspended in 50 μL 50 mM NH_4_HCO_3_. Protein samples underwent trypsin digestion (enzyme-to-substrate ratio of 1:50 at 37 °C for 18–20 h) in the filter and then were collected by centrifugation at 12,000× *g* for 15 min. Additional washing, twice with 200 μL of MS water, was essential to obtain greater yields. Finally, the centrifugate was dried by using the Concentrator plus (Eppendorf, Germany) for sub-sequential MS analysis.

### Phosphopeptide enrichment

For the phosphoproteomic analysis, peptides were extracted from the FFPE slides after trypsin digestion using the methods described above. The tryptic peptides were then enriched with High-Select™ Fe-NTA Phosphopeptide Enrichment Kit (Thermo Scientific cat. A32992), following the manufacturer’s recommendation. Briefly, peptides were suspended with binding/wash buffer (contained in the enrichment kit), mixed with the equilibrated resins, and incubated at 21–25 °C for 30 min. After incubation, the resins were washed thrice with binding/wash buffer and twice with water. The enriched peptides were eluted with elution buffer (contained in the enrichment kit) and dried in a Concentrator plus (Eppendorf, Germany).

### LC-MS/MS analysis

LC-MS/MS were performed on Easy-nLC liquid chromatography system (Thermo Scientific) coupled to an Orbitrap Fusion Lumos Tribrid platform with FAIMS (Thermo Fisher Scientific).

The peptides were dissolved with 10 μL loading buffer (5% methanol and 0.2% formic acid), and 5 μL was loaded onto a 360 μm I.D. × 2 cm, C18 trap column at a maximum pressure 280 bar with 12 μL solvent A (0.1% formic acid in water). Peptides were separated on 150 μm I.D. × 30 cm column (C18, 1.9 μm, 120 Å, Dr. Maisch GmbH) with a linear 5%–35% Mobile Phase B (ACN and 0.1% formic acid) at 600 nL/min for 150 min. FAIMS separations were performed with the following settings: inner electrode temperature = 100 °C (except where noted), outer electrode temperature = 100 °C, FAIMS carrier gas flow = 2.3 L/min. The dispersion voltage (DV) was set at −5000 V, and the compensation voltage was stepped into 40 V, 55 V and 70 V.

These analyses utilized a 120,000 resolving power survey scan with AGC = 3,000,000, followed by MS/MS of the most intense precursors for 80 ms. The MS/MS analyses were performed by 1.6 m/z isolation with the quadrupole, normalized HCD (higher-energy collisional dissociation) collision energy of 27%, and analysis of fragment ions in the ion trap using the “Turbo” speed scanning from 200 to 1200 m/z. Dynamic exclusion was set to 12 s. Monoisotopic precursor selection (MIPS) was set to Peptide, maximum injection time was set to 20 ms, AGC target was set to 20,000, and charge states unknown, +1, or > +5 were excluded and the advanced peak determination was toggled on.

For the phosphoproteomic analysis, the phosphopeptides were analyzed on FAIMS interfaced Orbitrap Fusion Lumos Tribrid Mass Spectrometer (Thermo Fisher Scientific, Rockford, IL, USA) equipped with an Easy nLC-1000 (Thermo Fisher Scientific, Rockford, IL, USA) and a Nanoflex source (Thermo Fisher Scientific, Rockford, IL, USA). Dried peptide samples re-dissolved in buffer A (0.1% FA in water) were loaded to a 2 cm self-packed trap column using buffer A and separated on a 150 μm inner diameter column with a length of 30 cm over a 150 min gradient (buffer A: 0.1% FA in water; buffer B: 0.1% FA in 80% ACN) at a constant flow rate of 600 nL/min (0–150 min, 0 min, 4% B; 0–10 min, 4%–15% B; 10–125 min, 15%–30% B; 125–140 min, 30%–50% B; 140–141 min, 50%–100% B; 141–150 min, 100% B). The eluted phosphopeptides were ionized and detected. Compensation Voltages (CV) among –30 V, –60 V, and –120 V were interrogated to find precursor rich CVs. Mass spectra were acquired over the scan range of m/z 350–1500 at a resolution of 120,000 (AUG target value of 5E5 and max injection time 50 ms). For the MS2 scan, the higher-energy collision dissociation fragmentation was performed at a normalized collision energy of 30%. The MS2 AGC target was set to 1e4 with a maximum injection time of 10 ms, peptide mode was selected for monoisotopic precursor scan, and charge state screening was enabled to reject unassigned 1 +, 7 +, 8 +, and > 8 + ions with a dynamic exclusion time of 45 s to discriminate against previously analyzed ions between ±10 ppm.

### Metabolites extraction

Lipid extraction MS water (200 mL) and methanol (240 mL) was added to a sample aliquot, and the tube was vortexed. After grinding beads was added to each tube, the grinding tube was placed in the precooled adapter, the frequency of the grinding instrument was set to be 60 Hz, the grinding operation to be 15 s, the grinding interruption to be 5 s, and the grinding operation times to be 10 min. Then, 800 mL of methyl tert-butyl ether (MTBE) was added, and the mixture was placed in the ultrasonic cleaner for ice bath ultrasonic for 30 min. After the mixture was centrifuged at 14,000× *g* for 10 min, the upper (organic) phase was collected and dried.

### LC-MS/MS analysis of metabolites

LC-MS/MS of lipids were performed on Easy-nLC liquid chromatography system (Thermo Scientific) coupled to Q Exactive HFX platform (Thermo Fisher Scientific). A 2.1 mm I.D. 3 100 mm column (Waters, Acclaim C30) was balanced with 70% solvent A (10 mM ammonium formate and 60% ACN in water). The lipids were dissolved with 10 mL loading buffer (50% isopropyl alcohol (IPA) and 50% ACN), and 5 mL was loaded onto a 2.1 mm I.D. 3 100 mm column (Waters, Acclaim C30) at 0.26 mL/min. Lipids were separated with a linear 30%–100% Mobile Phase B (10 mM ammonium formate and 0.1% formic acid, 90% IPA in ACN) for 20 min. These analyses utilized a 120,000 resolving power survey scan with AGC = 1,000,000, followed by MS/MS of the most intense precursors for 80 ms. The MS/MS analyses were performed by 1.5 m/z isolation with the quadrupole, normalized HCD (higher-energy collisional dissociation) collision energy of 20%, 40%, and 60% and analysis of fragment ions in the ion trap scanning from 200 to 2000 m/z. Maximum injection time was set to 20 ms, AGC target was set to 200,000.

### Metabolite identification and quantification

Metabolite profiles were analyzed by LipidSearch 4.2 (Thermo Fisher Scientific, CA, USA), a leading commercial lipidomics software platform^75^. The target database was Q Exactive HFX and the peak detection was recalc isotope. The search options were as follows: parent tolerance, 5 ppm, product tolerance, 8 ppm; m-score threshold, 2; Quan *m*/*z* tolerance ± 5 ppm; Quan retention time (RT) range ± 0.5 min; use of main isomer filter and for the ID quality filter, A-B; adduct ions, H+ and NH4+ for positive ion mode and H− and HCOO− for negative ion mode. Lipid alignments were performed with below parameters: ExpType, LC-MS; Alignment method, Mean; R. T. Tolerance, 2; Calculate unassigned peak area, on; Filter Type, New Filter; Toprank Filter, on; Main Node Filter, Main isomer peaks; m-Score Threshold, 5.0; ID quality filter: A, B, C and D. The results were extracted using LipidSearch 4.1.3 software. Finally, the lipids were manually filtered according to the following rules:

1. The peak areas of lipids with m-Score < 10 were revised to 0;
2. The peak areas of the lipids with AreaScore < 0.7 were revised to 0;
3. The peak areas of the lipids with PeakQuality < 0.9 were revised to 0;
4. The peak areas of the lipids with Occupy < 5 were revised to 0;
5. The peak areas of the lipids with Grade C and D were revised to 0.

### MS database searching peptide and protein identification

MS raw files were processed with a “Firmiana” (a onestop proteomic cloud platform)^76^ against the human National Center for Biotechnology Information (NCBI) RefSeq protein database using Mascot 2.4 (Matrix Science Inc., London, UK). The maximum number of missed cleavages was set to two. Mass tolerances of 10 ppm for the precursor and 10 ppm for production were allowed. The fixed modification was cysteine carbamidomethylation, while the variable modifications were N-acetylation and methionine oxidation. For the quality control of protein identification, the target-decoy-based strategy was applied to confirm that the false discovery rate (FDR) of both peptides and proteins was lower than 1%. The program percolator was used to obtain the probability value (*q* value) and showed that the FDR (measured by the decoy hits) of every peptide–spectrum match (PSM) was lower than 1%. All peptides shorter than seven amino acids were removed. The cutoff ion score for peptide identification was set at 20. All PSMs in all fractions were combined for protein quality control, which was a stringent quality control strategy. The *q* values of both target and decoy peptide sequences were dynamically increased employing the parsimony principle until the corresponding protein FDR was less than 1%. Finally, to reduce the false positive rate, proteins with at least two unique peptides were selected for further investigation.

Phosphoproteome MS raw files were searched against the human RefSeq protein database using Proteome Discoverer (version 2.3.0.523) with a Mascot^77^ (version 2.3.01) engine with a percolator^78^. Carbamidomethyl cysteine was used as a fixed modification, and oxidized methionine, protein N-term acetylation, and phospho (S/T/Y) were set as variable modifications. The false discovery rate (FDR) of peptides and proteins was set at 1%. The tolerance for spectral searches a mass tolerance of 20 ppm for the precursor. The maximum number of missing cleavage site was set at 2. For phosphosite localization, ptmRS^79^ was used to determine phosphosite confidence and a phosphosite probability > 0.75 was used for further analysis.

### Label‑free‑based MS quantification of proteins

The one-stop proteomic cloud platform, “Firmiana,” was further employed for protein quantification. The identification results and the raw data from the mzXML files were loaded. Then, for each identified peptide, the extracted-ion chromatogram (XIC) was extracted by searching against MS1 based on its identification information, and the abundance was estimated by calculating the area under the extracted XIC curve. For protein abundance calculation, the non-redundant peptide list was used to assemble proteins following the parsimony principle. Protein abundance was then estimated by a traditional label-free, intensity-based absolute quantification (iBAQ) algorithm, which divided protein abundance (derived from identified peptide intensities) by the number of theoretically observable peptides^80,81^. The fraction of total (FOT), a relative quantification value that was defined as a protein’s iBAQ divided by the total iBAQ of all identified proteins in one experiment, was calculated as the normalized abundance of a particular protein in the experiments. Finally, the FOT was further multiplied by 1e6 for the ease of presentation, and NA values were replaced with 1e−5 to adjust extremely small values.

For the phosphoproteomic data, the intensities of the phosphopeptides were extracted from the Proteome Discover (version 2.3). For the phosphoprotein abundance calculation, the non-redundant phosphor-peptide list was used to assemble the proteins by following the parsimony principle. Next, the phosphoprotein abundance was estimated by a traditional label-free, iBAQ algorithm, which divided the protein abundance (derived from the intensities of the identified peptides) by the number of theoretically observable peptides^81^. For phosphosite localization, the ptmRS^79^ was used to determine phosphosite confidence and phosphosite probability > 0.75 is considered as confident phosphosites.

### Quality control of the MS data

For the quality control of the performance of MS, the HEK293T cell (National Infrastructure Cell Line Resource) lysate was measured every 3 days as the quality control standard. The quality control standard was digested and analyzed using the same method and conditions. A pairwise Spearman’s correlation coefficient was calculated for all quality control runs in the statistical analysis environment R (version 4.0.2). The average correlation coefficient among the standards was 0.9, demonstrating the consistent stability of the mass spectrometry platform.

### Kinase activity prediction

To estimate changes in kinase activity, we performed kinase enrichment analysis on significantly differentiated phosphosites in tumors compared to NATs, for intestinal-type and pancreatobiliary-type or each subtype via kinase–substrate enrichment analysis (KSEA)^82^. Known kinase–substrate site relationships from PhosphoSitePlus (PSP)^83^ or NetworKIN 3.0^84^ with scores greater than 1 were used for kinase–substrate analysis. A kinase score was given for each kinase based exclusively on the collective phosphorylation status of its substrates and transformed into a *z*-score. For kinase enrichment analysis, the threshold used for significantly enriched kinases was *p* < 0.05.

### Missing value imputation

For the proteomic and phosphoproteomic data, FOTs multiplied by 1e5 were used for quantification, and missing values were imputed with 1e−5 and finally, log2-transformed, if necessary.

### Differential protein analysis

Differential protein analysis Student *t*-test was used to examine whether proteins were differentially expressed between the tumors and NATs. Upregulated or downregulated proteins in tumors were defined as proteins differentially expressed in tumors compared with NATs (T/NAT > 2 or < 1/2, Wilcoxon rank-sum test, Benjamini–Hochberg adjusted *p* < 0.05). Kruskal–Wallis test was used to examine whether proteins were differentially expressed between patients with different histological subtypes (Benjamini–Hochberg adjusted *p* < 0.05). Kruskal–Wallis test was used to examine whether proteins were differentially expressed among three proteomic subtypes and immune subtypes (Benjamini–Hochberg adjusted *p* < 0.05).

### Pathway enrichment analysis

Differentially expressed genes were subjected to gene ontology and KEGG pathway enrichment analysis in DAVID^85^ with a *p* value/FDR < 0.1. We used gene sets of molecular pathways from the KEGG^86^/Hallmark^87^/Reactome^88^/GO^89^ databases to compute pathways.

### Pathway scores and correlation analysis

ssGSEA^90^ was utilized to obtain pathway scores for each sample based on RNA-seq, proteomic, and phosphoproteomic data using the R package GSVA^91^. Correlations between the pathway scores and other features were determined using Spearman’s correlation. Inferred activity was performed using ssGSEA implemented in the R package GSVA with a minimum gene set size of 10. The transcriptional targets of TFs mentioned in this work were collected from the ENCODE Project Consortium^92^ and used to infer TF activity via ssGSEA.

### Cell cycle analysis

Multi-gene proliferation scores (MGPSs) were calculated as the mean expression level of all cell cycle-regulated genes in each sample as described previously^93,94^. Briefly, MGPS was calculated from the mean normalized proteomic data in each sample in our study.

### Phosphopeptide analysis-kinase and substrate regulation

KSEA algorithm was used to estimate the kinase activities based on the abundance of phosphosites. Kinase-substrate enrichment analysis (KSEA) estimates changes in a kinase’s activity by measuring and averaging the amounts of its identified substrates instead of a single substrate, which enhances the signal-to-noise ratio from inherently noisy phosphoproteomic data^82,95^. If the same phosphorylation motif was shared by multiple kinases, it was used for estimating the activities of all known kinases. The use of all curated substrate sequences of a particular kinase minimizes the overlapping effects from other kinases and thus improves the precise measurement of kinase activities. The information of kinase-substrate relationships was obtained from publicly available databases including PhosphoSite^38^, Phospho.ELM^36^, and PhosphoPOINT^37^. The information of substrate motifs was obtained either from the studies^96^ or from an analysis of KSEA dataset with Motif-X^82^.

### Protein–protein interaction network construction

Interaction network among the proteins and phosphorylated proteins was generated with STRING v 11.0 (https://string-db.org/) using medium confidence (0.4), and experiments and database as the active interaction sources. The network was visualized using Cytoscape version 3.8.0^97^.

### Consensus clustering analysis

The protein expression matrix of the 198 tumor samples was used to identify the proteomic clusters using the consensus cluster method. Consensus clustering was performed using the ConsensusClusterPlus (R package ConsensusClusterPlus v.1.48.0)^98,99^, with the top 50% most varied protein. The following detail settings were used for clustering: number of repetitions = 10,000 bootstraps; pItem = 0.8 (resampling 80% of any sample); pFeature = 1 (resampling 100% of any protein); and clusterAlg = “pam”; and distance = “spearman. The number of clustering was determined by three factors, the average pairwise consensus matrix within consensus clusters, the delta plot of the relative change in the area under the cumulative distribution function (CDF) curve, and the average silhouette distance for consensus clusters. The consensus matrices for k = 2, 3, 4, and 5 clusters are shown in Supplementary Fig. S7a. A consensus matrix with k = 3 appeared to yield the clearest cut between clusters and showed a significant association with the patient survival.

### Correlation between proteomic subtypes and clinical features

For the purpose of measuring correlations between proteomic subtypes and clinical features, Fisher’s exact test was performed on histological subtypes, genomic alterations, gender, smoke status, alcohol habit, vessel invasion, fascicular invasion, metastasis, lymph node metastasis and TNM stage.

### Survival analysis

Kaplan–Meier survival curves (log-rank test) were used to determine the overall survival (OS) and progression-free survival (PFS) of proteomic subtypes and patients. The coefficient value, which is equal to ln (HR), was calculated using Cox proportional hazards regression analysis. *p* values less than 0.05 were considered significantly different and selected for Cox regression multivariate analysis. Prior to the log-rank test of a given protein, phosphoprotein, or phosphosite, survminer (version 0.2.4, R package) with maximally selected rank statistics (https://rpkgs.datanovia.com/survminer/reference/surv_cutpoint.html) was used to determine the optimal cutoff point for the selected samples according to a previous study^100^. OS curves were then calculated (Kaplan–Meier analysis, log-rank test) based on the optimal cutoff point.

### Effects of copy number alterations

SCNAs affecting mRNA and protein/phosphoprotein abundance in either “*cis*” (within the same aberrant locus) or “trans” (remote locus) mode were visualized by multiOmicsViz (R package)^101^ (diagonal patterns in Fig. 2a). Spearman’s correlation coefficients and associated multiple-test adjusted *p* values were calculated for all CNV–mRNA pairs, CNV–protein pairs and CNV–phosphoprotein pairs, respectively. The usage of the *cis* effect in this work was followed by the definition provided in the previous published research^101^, which defined the impact of copy number alteration (CNA) on the same loci protein or mRNA abundance as the *cis* effect, and the *trans* effect was defined as chromosomal loci whose alteration is significantly associated with abundance changes of many transcripts or proteins at other loci.

### Defining cancer‑associated genes

Cancer-associated genes (CAG) were compiled from genes defined by Bailey et al.^102^ and cancer-associated genes listed in Mertins et al.^103^ and adapted from Vogelstein et al.^104^. Gene Set Enrichment Analysis (GSEA) was performed by the GSEA software (https://www.gsea-msigdb.org/gsea/index.jsp). Gene sets including KEGG, GO Biological Process (BP), Reactome, and HALLMARK downloaded from the Molecular Signatures Database (MSigDB v7.1, http://software.broad institute.org/gsea/msigdb/index.jsp) were used.

### Immune subtype analysis

The immune score, stromal score and tumor purity were inferred using the R package ESTIMATE v1.0.11 using transcriptome data (Supplementary Table S5c)^105^. The abundances of 64 different cell types for AMPAC samples in protein level were computed via xCell (https://xcell.ucsf.edu/) (Supplementary Table S5a). Based on these 64 signatures, consensus clustering was performed in order to identify groups of samples with similar immune/stromal characteristics. Consensus clustering was performed using the R package ConsensusClusterPlus^98^. Consensus Cluster Plus parameters were reps = 1000, pItem = 0.8, pFeature = 1, clusterAlg = “pam,” distance = “spearman.” As summarized in Fig. 7, the clustering analysis of the tumors (vertical column) by xCell score (horizontal rows) divided 198 samples into three immune clusters. A consensus matrix with k = 3 appeared to have the clearest cut between clusters and showed significant association with the patients’ survival.

### IHC

Formalin-fixed, paraffin-embedded tissue sections of 10 μM thickness were stained in batches for detecting HADH, MTAP, PCNA, ANO1, PRKCI, PDGFRB, CD34, ACLY, FASN, PRKDC, IL-6, STAT1, CD4 in a central laboratory at the Zhongshan Hospital according to standard automated protocols. Deparaffinization and rehydration were performed, followed by antigen retrieval and antibody staining. HADH, MTAP, PCNA, ANO1, PRKCI, PDGFRB, CD34, ACLY, FASN, PRKDC, IL-6, STAT1 and CD4 IHC were performed using the Leica BONDMAX auto staining system (Roche). HADH Polyclonal antibody (Proteintech, Cat No. 19828-1-AP), MTAP Polyclonal antibody (Proteintech, Cat No. 11475-1-AP), PCNA Polyclonal antibody (Proteintech, Cat No. 10205-2-AP), ANO1/TMEM16A Polyclonal antibody (Proteintech, Cat No. 12652-1-AP), PKC Iota Polyclonal antibody (Proteintech, Cat No. 13883-1-AP), PDGFR beta Polyclonal antibody (Proteintech, Cat No. 13449-1-AP), CD34 Polyclonal antibody (Proteintech, Cat No. 14486-1-AP), ACLY Monoclonal antibody (Proteintech, Cat No. 15421-1-AP), FASN Monoclonal antibody (Proteintech, Cat No. 66591-1-Ig), DNA-PKcs Polyclonal antibody (Proteintech, Cat No. 19983-1-AP), IL-6 Polyclonal antibody (Proteintech, Cat No. 21865-1-AP), STAT1 Monoclonal antibody (Proteintech, Cat No. 66545-1-Ig), and CD4 Monoclonal antibody (Proteintech, Cat No. 67786-1-Ig) were introduced, followed by detection with a Bond Polymer Refine Detection DS9800 (Bond). Slides were imaged using an OLYMPUS BX43 microscope (OLYMPUS) and processed using a ScanScope (Leica).

### Cell lines

Human ampullary adenocarcinoma cell lines including SNU-869 and SNU-478 were obtained for Chinese Academy of Sciences (Shanghai, China). All cell lines were routinely tested for mycoplasma contamination and authenticated by Short Tandem repeat (STR) profiling. Cells were maintained in recommended medium, Roswell Park Memorial Institute-1640 (RPMI-1640, Corning) or DMEM (ATCC) supplemented with 10% fetal bovine serum (FBS, Sigma‐Aldrich) and 1% penicillin–streptomycin antibiotic (Sigma‐Aldrich) and incubated at 37 °C and 5% CO_2_ in a humidified atmosphere in an incubator.

### Plasmids

The sequence of human PDGFRB, ANO1 and PRKCI open reading frame was obtained using Polymerase chain reaction (PCR) from CDNA. The PCR fragment was inserted into pCMV-N-Flag, pcDNA3.1 Myc HisA by the recombinant method and was confirmed by sequencing identification.

### Cell transfections

Plasmid transfections were carried out by the polyethylenimine (PEI), Lipofectamine 3000 (Invitrogen), and Lipofectamine 2000 (Invitrogen) methods. In the PEI transfection method, 400 μL of DMEM (serum-free medium) and the plasmid were placed in an empty EP tube and PEI was added into the medium. The mixture was incubated for 15 min. Meanwhile, the cell culture medium was replaced with fresh 10% FBS medium. After 15 min, the mixture was added to the cells, and the fresh medium was replaced after 12–16 h. After 36–48 h, the transfection was completed. In the Lipofectamine 3000 transfection method, DMEM (250 μL) was added to two empty EP tubes and Lipofectamine 3000 was added to one of the tubes and mixed for 5 min. The plasmid and P3000 were added in the other tube and then added to the medium containing Lipofectamine 3000, mixed, and allowed to stand for 5 min. Meanwhile, the cell culture medium was replaced with fresh 10% FBS medium. After 5 min, the mixture was added to the cells, and the fresh medium was replaced after 12 h. After 36–48 h, the transfection was completed and the cells were treated. In the Lipofectamine 2000 transfection method, 125 μL of DMEM (serum-free medium) and the siRNA were placed in an empty EP tube. A total of 125 μL of DMEM (serum-free medium) and the Lipofectamine 2000 were placed in another empty EP tube then added to the medium containing siRNA. The mixture was incubated for 5 min. Meanwhile, the cell culture medium was replaced with fresh 10% FBS medium. After 5 min, the mixture was added to the cells. After 36–48 h, the transfection was completed.

### Gene silencing

To generate cells stably knockdown for HADH, MTAP was transfected into cells, using pCMV-VSVG and pCMV-Gag as packaging plasmids. Twenty-four hours after transfection, the virus supernatant was collected to infect target cells. Puromycin was used to select stable cells for ~7 days.

HADH siRNA-homo-sense: 5′-GGACTGGATACTACGAAGTTC-3′

HADH siRNA-homo-antisense: 5′-GAACTTCGTAGTATCCAGTCC-3′

MTAP siRNA-homo-sense: 5′-AAAAUUAAGGCAUCAGAUGGC-3′

MTAP siRNA-homo-antisense: 5′-CAUCUGAUGCCUUAAUUUUGG-3′

### Transwell migration assays

Cell migration assays were performed with 24-well transwells (8-μm pore size, Falcon). In total, 1.5 × 10^5^ transfected cells were suspended in serum-free DMEM medium and added to the upper chamber, and 700 μL DMEM with 10% FBS was placed in the lower chamber. After 16 h of incubation, cells on the lower surface of membrane were fixed in 4% paraformaldehyde and stained with crystal violet. Cells in six microscopic fields were counted and photographed.

### AMPAC cells proteome

For the proteomic analysis of AMPAC cells, cells were lysed in lysis buffer (8 M Urea, 100 mM Tris Hydrochloride, pH 8.0) containing protease and phosphatase Inhibitors (Thermo Scientific) followed by 1 min of sonication (3 s on and 3 s off, amplitude 25%). The lysate was centrifuged at 14,000× *g* for 10 min and the supernatant was collected as whole tissue extract. Protein concentration was determined by Bradford protein assay. Extracts from each sample (500 μg protein) was reduced with 10 mM dithiothreitol at 56 °C for 30 min and alkylated with 10 mM iodoacetamide at room temperature (RT) in the dark for additional 30 min. Samples were then digested using the filter-aided proteome preparation (FASP) method with trypsin. Briefly, samples were transferred into a 30 kD Microcon filter (Millipore) and centrifuged at 14,000× *g* for 20 min. The precipitate in the filter was washed twice by adding 300 μL washing buffer (8 M urea in 100 mM Tris, pH 8.0) into the filter and centrifuged at 14,000× *g* for 20 min. The precipitate was resuspended in 200 μL 100 mM NH_4_HCO_3_. Trypsin with a protein-to enzyme ratio of 50:1 (w/w) was added into the filter. Proteins were digested at 37 °C for 16 h. After tryptic digestion, peptides were collected by centrifugation at 14,000× *g* for 20 min and dried in a vacuum concentrator (Thermo Scientific). Dried peptides were then used for proteomic analysis.

### Immunoprecipitation

For immunoprecipitation, cells were lysed with 0.5% NP-40 buffer containing 50 mM Tris-HCl (pH 7.5), 150 mM NaCl, 0.3% NONIDET P-40, 1 μg/mL aprotinin, 1 μg/mL leupeptin, 1 μg/mL pepstatin, and 1 mM PMSF. Cell lysates were incubated with Flag beads (Sigma) for 3 h at 4 °C. The binding complexes were washed with 0.5% NP-40 buffer and mixed with loading buffer for SDS-PAGE.

### IP-MS for ANO1

The AMPAC SNU-478 cell line (SNU-478-*ANO1*-OE and SNU-478-vector) were lysed on ice in 0.5% NETN buffer (0.5% Nonidet P-40, 50 mM Tris-HCl (pH 7.4), 150 mM NaCl, 1 mM EDTA, and protease inhibitor mixture). After the removal of insoluble cell debris by highspeed centrifugation, protein concentration was then determined by Braford assay. Then 2 mg proteins were incubated with ANO1/TMEM16A Polyclonal antibody (1:100 dilution, Proteintech, Cat No. 12652-1-AP) and rotated overnight at 4 °C. Further, 20 μL Pre-wash magnetic beads (Protein A Magnetic Beads, #73778) were added for another 20 min incubation at room temperature. Pellet beads using magnetic separation rack. Wash pellets five times with 500 μL of 1x cell lysis buffer. Keep on ice between washes. Beads were further washed twice with ddH_2_O, and three times with 50 mM NH_4_HCO_3_. Then, “on-bead” tryptic digestion was performed at 37 °C overnight. The peptides in the supernatant were collected by centrifugation and dried in a speed vacuum (Eppendorf). Lastly, the samples were redissolved in loading buffer containing 0.1% formic acid before being subjected to MS.

### Quantitative RT‑PCR

The Superscript III RT kit (Invitrogen) was used with random 3 hexamer primers to produce cDNA from 4 μg total RNA. ACTIN was used as the endogenous control for samples. All primers for analysis were synthesized by TSINGKE Biological Technology (Shanghai). The analysis was performed by using an Applied Biosystems 7900HT Sequence Detection System, with SYBR green labeling.

QPCR-ANO1-F: 5′-CAAGT TTGGC TACAG CACGC-3′

QPCR-ANO1-R: 5′-AGACT AGGGA GCGAC GAAGT-3′

QPCR-PRKCI-F: 5′-GACGC AGGAG GTGTC TTGG-3′

QPCR-PRKCI-R: 5′-CTTGG CTTGG AAAGT GTGGC-3′

QPCR-PDGFRB-F: 5′-CCATC AGCAG CAAGG CGA-3′

QPCR-PDGFRB-R: 5′-CCAGA AAAGC CACGT TGGTG-3′

QPCR-HADH-F: 5′-AACTC GGGTT TGGGC TTTTC-3′

QPCR-HADH-R: 5′-TTTAA GGATG GGCTG GGCTG-3′

QPCR-MTAP-F: 5′-CGTGA AGGTG AGATG AGCCC-3′

QPCR-MTAP-R: 5′-TGTTC GCCTG GTAGT TGACC-3′

### Cell proliferation assay

Different groups of cells (2000 cells/well) were seeded into 96-well plates. At the indicated detection times, CCK8 reagent was added into each well. The plates were incubated at 37 °C for 1 h, and then, absorbance of the 96-well plates was detected at a wavelength of 450 nm.

### Xenograft tumorigenesis experiments

Different groups of ampullary adenocarcinoma cells (5 × 10^6^) were re-suspended in PBS and injected subcutaneously (SC) into the right flank of 5-week-old BALB/c-nude mice. The weight and the tumor diameter of each mouse were measured every week. Tumor volume (mm3) was calculated as follows: (shortest diameter)^2^ × (longest diameter) × 0.5. Four weeks later all mice were killed.

### Quantification and statistical analysis

Statistical details of experiments and analyses were noted in the figure legends and supplementary tables. Standard statistical tests were used to analyze the association between clinical information and multi-omics data. Student’s *t*-test, Wilcoxon rank-sum test, one-way ANOVA, and Kruskal–Wallis test were used for continuous data; Fisher’s exact test and *χ*^2^ test was used for categorical data. The Benjamini–Hochberg adjusted *p* values of differentially expressed RNA/proteins/phosphoproteins were calculated. Log-rank tests and Kaplan–Meier survival curves were used to compare the overall survival and progression-free survival. All statistical tests were two-sided, and statistical significance was considered when *p* < 0.05. Variables associated with survival were identified using univariate Cox proportional hazards regression models. The correlation between two sets of data was calculated using Spearman’s correlation. All the analyses of clinical data were performed in R (version 4.0.2) and GraphPad Prism 8. For functional experiments, each was repeated at least three times independently, and results were expressed as mean ± standard error of the mean (SEM). Statistical analysis was performed using GraphPad Prism 8.

## Supplementary information

Supplementary Table S1

Supplementary Table S2

Supplementary Table S3

Supplementary Table S4

Supplementary Table S5

Supplementary Information

## Data Availability

The proteomic data and phosphoproteomic data (MS raw data and the Mascot output tables) generated in this research have been deposited in the ProteomeXchange Consortium via the iProX partner repository (http://www.iprox.cn/) under Project ID IPX0002195000. The raw WES data and Transcriptomic data have been deposited in the National Genomics Data Center (GSA) database under accession code HRA005749 and HRA005872, respectively. The raw sequencing data are available under controlled access due to data privacy laws related to patient consent for data sharing and the data should be used for research purposes only. Access can be obtained by approval via their respective DAC (Data Access Committees) in the GSA-human database. According to the guidelines of GSA-human, all non-profit researchers are allowed access to the data and the Principle Investigator of any research group is allowed to apply for Controlled access of the data. The user can register and login to the GSA database website (https://ngdc.cncb.ac.cn/gsa-human/) and follow the guidance of “Request Data” to request the data step by step (https:// ngdc.cncb.ac.cn/gsahuman/document/GSAHuman_Request_Guide_for_Users_us.pdf). The approximate response time for accession requests is about 2 weeks. The access authority can be obtained for Research Use Only. The user can also contact the corresponding author directly. Once access has been granted, the data will be available to download for 3 months. The remaining data are available within the Article, Supplementary information, or Source Data file. Human reference genome (hg19 assembly) was downloaded from NCBI (https://www.ncbi.nlm.nih.gov/). The information of kinase-substrate relationships were available in PhosphoSite (https://www.phosphosite.org/homeAction.action)^38^, Phospho.ELM (http://phospho.elm.eu.org)^36^, and PhosphoPOINT (http://kinase.bioinformatics.tw/)^37^. Source data are provided with this paper.
