# Supplementary material for: Integrated proteogenomic characterization of ampullary adenocarcinoma
Source: Cell Discov. 2025 Jan 7;11:2. doi: 10.1038/s41421-024-00742-4 (PMC11704194; doi:10.1038/s41421-024-00742-4)
Supplement: Supplementary file 6 — Supplementary Information [file 41421_2024_742_MOESM6_ESM.pdf]

Supplementary Fig. S1

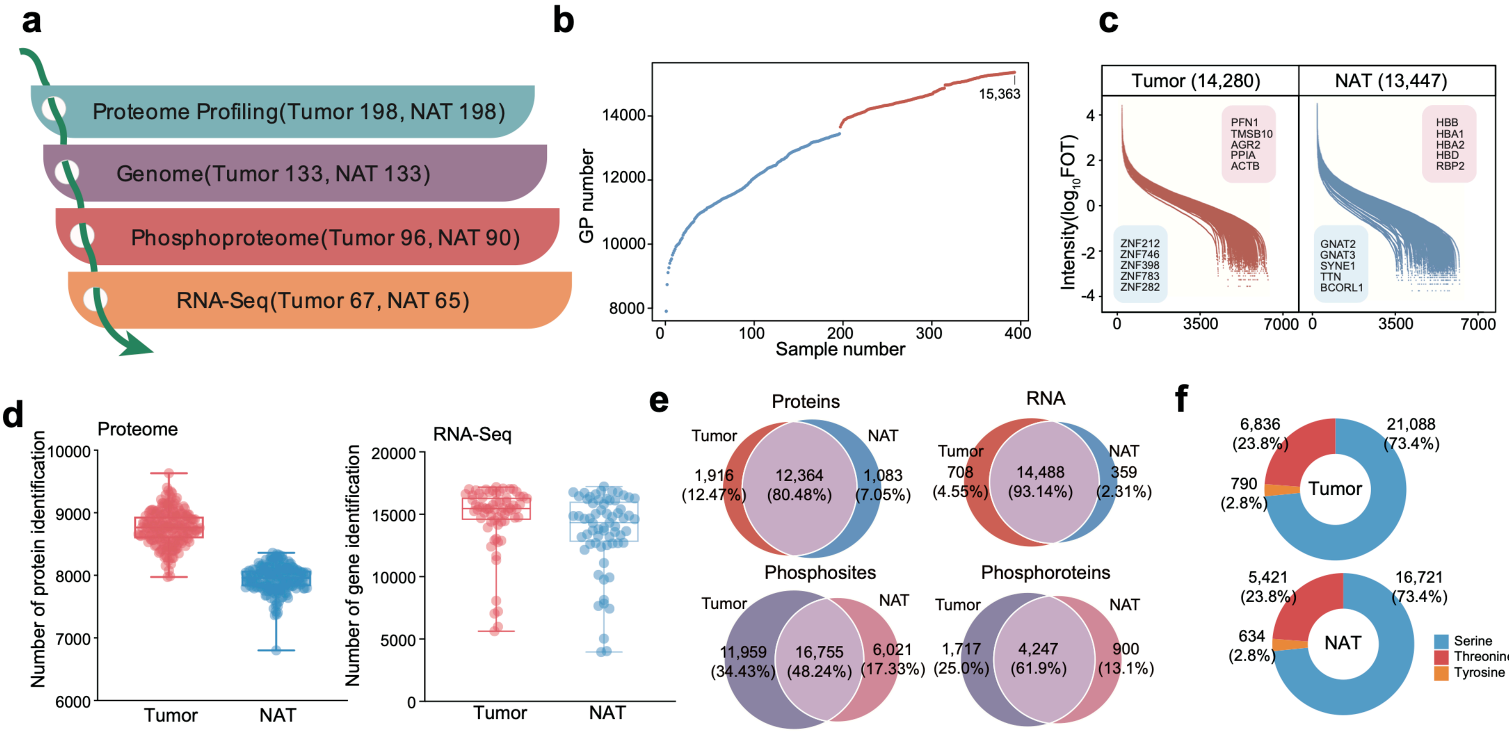

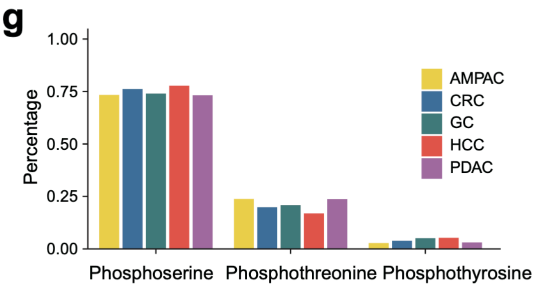

i

Frequencies of Altered Pathways

Pathways	Marie−Claude.et.al. (n = 93)	Shinichi.et.al (n = 159)	Fudan cohort (n = 133)
RTK-RAS signaling pathway (%)	86.02	71.07	84.21
Wnt signaling pathway (%)	51.02	60.37	59.40
TP53 signaling pathway (%)	77.42	62.89	57.14
TGF-β signaling pathway (%)	54.84	/	24.06

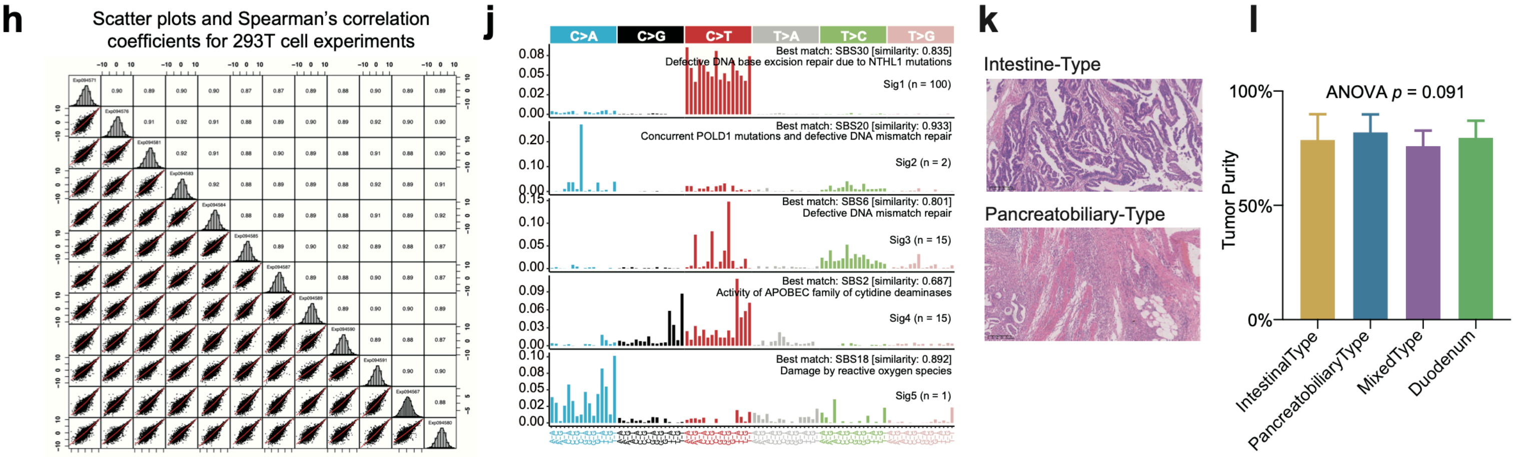

Supplementary Fig. S1 Proteogenomic landscape of AMPAC related to Fig. 1

a. The sample numbers in tumors and NATs at multi-omics levels.

b. The cumulative number of proteins identified in 396 proteome profiling samples.

c. Dynamic ranges of protein abundances identified in tumors and NATs.

d. The left boxplot illustrated the number of proteins identified among tumors (red) and NATs (blue). The right boxplot illustrated the number of gene identified among tumors (red) and NATs (blue).

e. Venn plots depicting the number of proteins (first panel), mRNAs (second panel), phosphosites (third panel), and phosphoproteins (fourth panel) detected in tumors and NATs.

f. Pie charts showing identified the types of phosphorylation sites in tumor (left panel), and NATs (right panel).

g. Phosphomodification sites in our AMPAC cohort compared with other tumors.

h. Longitudinal quality control of mass spectrometry using tryptic digest of HEK293T cells. The upper-right half of the panel represents the pairwise Spearman's correlation coefficients of the samples, and the bottom-left half of the panel depicts the pairwise scatter plots.

i. Mutational spectrum of the five mutational signatures extracted by Sigminer analysis. Corresponding COSMIC signatures are labeled in parentheses.

j. Frequency of altered pathways in this cohort compared with other studies.

k. Hematoxylin and eosin (H&E)-stained slides of tumor tissues for quality control.

l. Barplot for tumor purity among four pathological subtypes. (One-way ANOVA test).

Supplementary Fig. S2

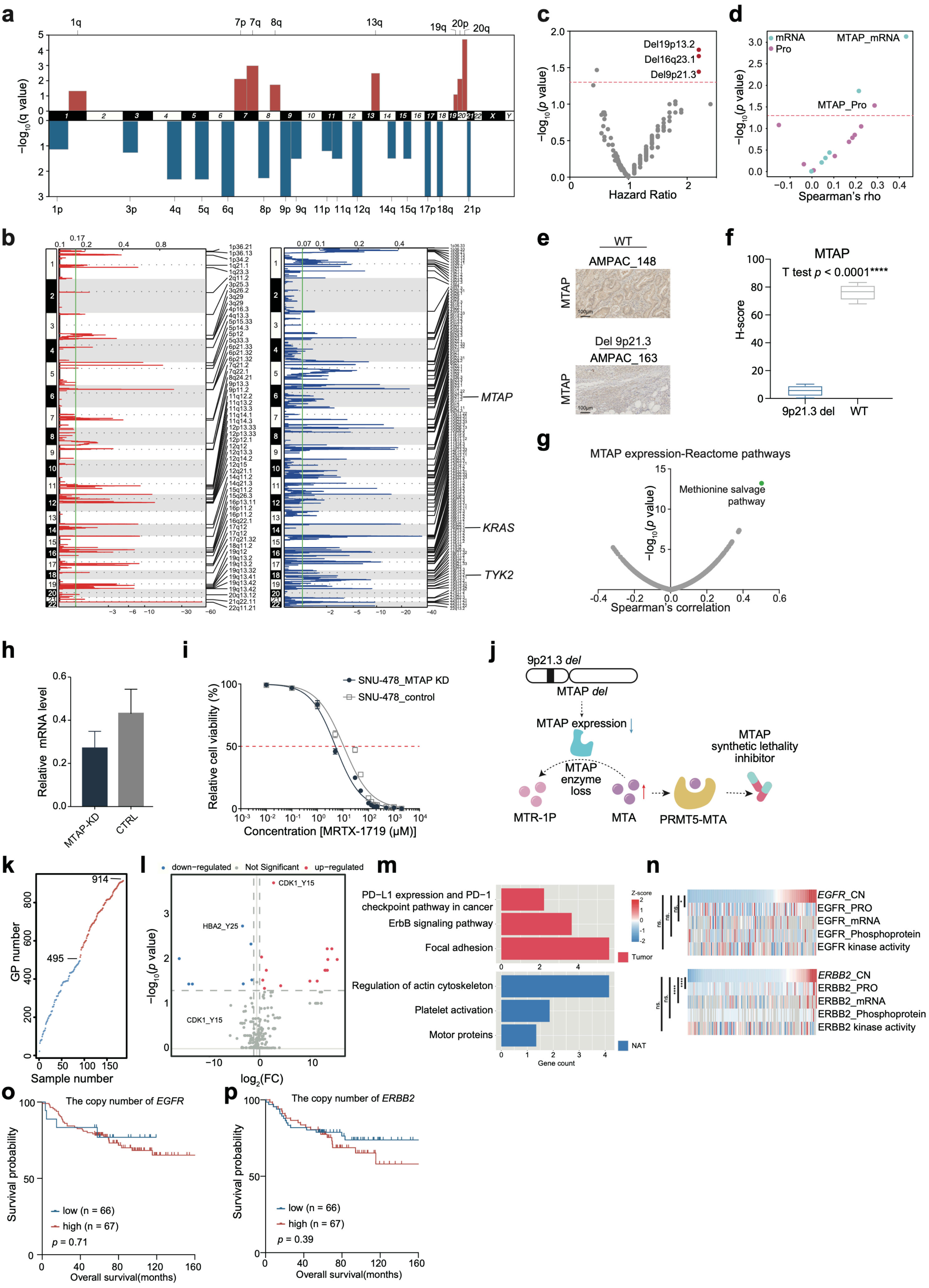

Supplementary Fig. S2 Somatic copy number variations in the AMPAC cohort related to Fig. 2 and Fig. 3

a. Arm-level CNVs. Red denotes amplification and blue denotes deletion.

b. GISTIC plots, G-scores are shown at the top and q values at the bottom, bars showing the locations of significant amplifications (left panel), and deletions (right panel) for each chromosome. The green vertical line is used to define statistical significance.

c. Peak alterations associated with prognosis (overall survival). Volcano plot showing log2-based hazard ratio for each alternative peak.

d. Left panel, the scatter plot described the correlation between MTAP protein (purple-colored dots) and mRNA (blue-colored dots) abundance and 9p21.3 copy number (Spearman correlation). Right panel, representative IHC staining illustrated MTAP was decreased in the 9p21.3 deletion group (Scale bar = 100 μm).

e. Representative IHC staining illustrated MTAP was decreased in the 9p21.3 deletion group (Scale bar = 100 μm).

f. Boxplot exhibited the H-score of MATP IHC images between 9p21.3 deleted samples and WT samples (Student t-test, $p < 0.0001$).

g. The volcano plot described the correlation between MTAP protein expression and GSVA scores (Spearman's correlation).

h. Relative mRNA abundance of MTAP in SNU-478 MTAP-KD and control groups.

i. Dose-response curve of MRTX-1719 in SNU-478 MTAP-KD and control groups.

j. Schematic plot exhibited the mechanism enhanced sensitivity to MTAP synthetic lethality inhibitors due to the downregulation of MTAP expression by 9p21.3 deletion through the *cis* effect.

k. The cumulative number of proteins identified in tumor (red dots) and NAT (blue dots) phosphoproteome samples.

l. Volcano plot showed differentially expressed tyrosine phosphosites in tumor (red dots) and NAT (blue dots) (Wilcoxon rank-sum test, $p < 0.05$, FC > 1.5).

m. Barplots indicating biological pathways upregulated in tumor (red bar, top) and NAT (blue bar, bottom) at the phosphoproteome level.

n. Top panel: The heatmap showed the copy number of *EGFR*, EGFR protein expression

abundance, EGFR mRNA expression abundance, EGFR phosphoprotein expression abundance, and EGFR kinase activity. Botton panel: Heatmap showed the copy number of *ERBB2*, ERBB2 protein expression abundance, ERBB2 mRNA expression abundance, ERBB2 phosphoprotein expression abundance, and ERBB2 kinase activity.

o. Kaplan-Meier curves for OS (analyzed samples: n = 133) based on the copy number of *EGFR*.

p. Kaplan-Meier curves for OS (analyzed samples: n = 133) based on the copy number of *ERBB2*.

Supplementary Fig. S3

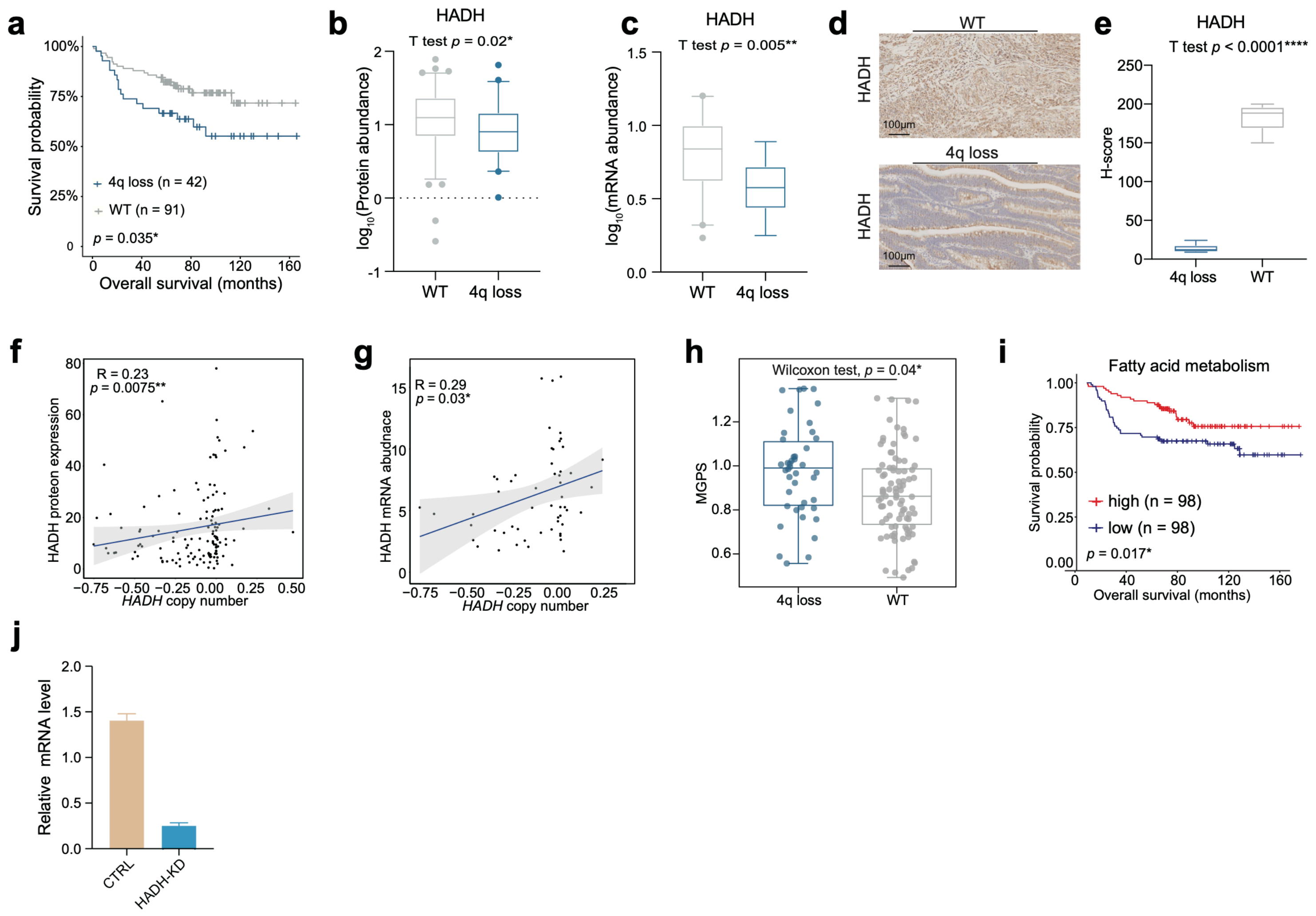

Supplementary Fig. S3 Downstream biological process of the cis effect of 4q loss Fig. 3

a. The Kaplan-Meier curve for overall survival based on chromosome 4q loss status (log-rank test, $p = 0.035$).

b. Boxplot exhibited the protein expression of HADH in the 4q loss samples and WT samples (Student t-test, $p = 0.02$).

c. Boxplot exhibited the mRNA expression of HADH in the 4q loss samples and WT samples (Student t-test, $p = 0.005$).

d. Representative IHC staining illustrated HADH was decreased in the 4q loss group (Scale bar = 100 μm).

e. Boxplot exhibited the H-score of HADH IHC images between the 4q loss samples and WT samples (Student t-test, $p < 0.0001$).

f. Spearman correlation of *HADH* copy number and HADH protein abundance (Spearman's r = 0.23, $p = 0.0075$).

g. Spearman correlation of *HADH* copy number and HADH mRNA abundance (Spearman's r = 0.29, $p = 0.03$).

h. Boxplot exhibited the MGPS between the 4q loss samples and WT samples (Wilcoxon test, $p = 0.04$).

i. The Kaplan-Meier curve for overall survival based on the score of the fatty acid metabolism pathway (log-rank test, $p = 0.017$).

j. Relative mRNA abundance of HADH in SNU-478 HADH-KD and control groups.

Supplementary Fig. S4

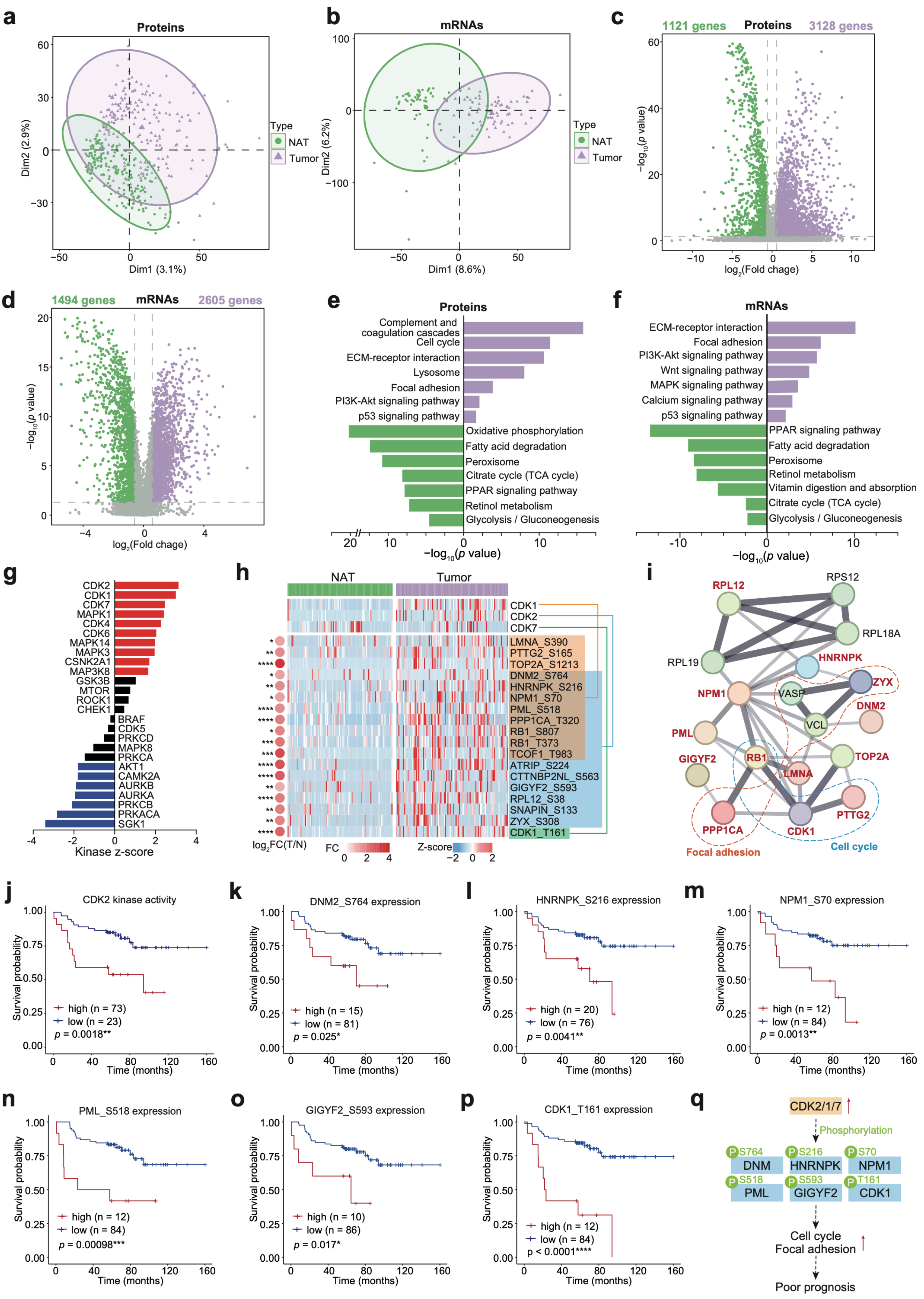

Supplementary Fig. S4 Integrated multi-omics features in tumor tissues compared with NATs of the AMPAC

a. PCA of proteomic data among tumor (purple, n = 198) and NAT (green, n = 198).

b. PCA of transcriptomic data among tumor (purple, n = 67) and NAT (green, n = 65).

c. Volcano plot showed differentially expressed genes in tumor (purple dots) and NAT (green dots) at the proteome level (Wilcoxon rank-sum test, $p < 0.05$, FC > 1.5).

d. Volcano plot showed differentially expressed genes in tumor (purple dots) and NAT (green dots) at the mRNA level (Wilcoxon rank-sum test, $p < 0.05$, FC > 1.5).

e. Bar plots indicating biological pathways upregulated in tumor (purple bar, right) and NAT (green bar, left) at the proteome level.

f. Bar plots indicating biological pathways upregulated in tumor (purple bar, right) and NAT (green bar, left) at the transcriptome level.

g. KSEA analyses of kinase activities in tumors and NATs.

h. Heatmap of activated kinases in tumors and substrates. Fold changes of the substrates are shown on the left (Kruskal-Wallis test). Substrates of CDK1 are listed with an orange background, substrates of CDK2 are listed with a blue background and substrate of CDK7 is listed with a green background.

i. The protein–protein interaction network constructed by the activated substrates of CDKs in tumor.

j-p. The Kaplan-Meier curve for overall survival based on (j). CDK2 kinase activity (log-rank test, p = 0.0018), (k). DNM2/S764 abundance (log-rank test, p = 0.025), (l). HNRNPK/S216 abundance (log-rank test, p = 4.1e-3), (m). NPM1/S70 abundance (log-rank test, p = 1.3e-3), (n). PML/S518 abundance (log-rank test, p = 9.8e-4), (o). GIGYF2/S593 abundance (log-rank test, p = 0.017), (p). CDK1/T161 abundance (log-rank test, p < 1e-4).

q. A brief model depicting the functional impact of activated kinase CDK2/1/7 in tumor.

Supplementary Fig. S5

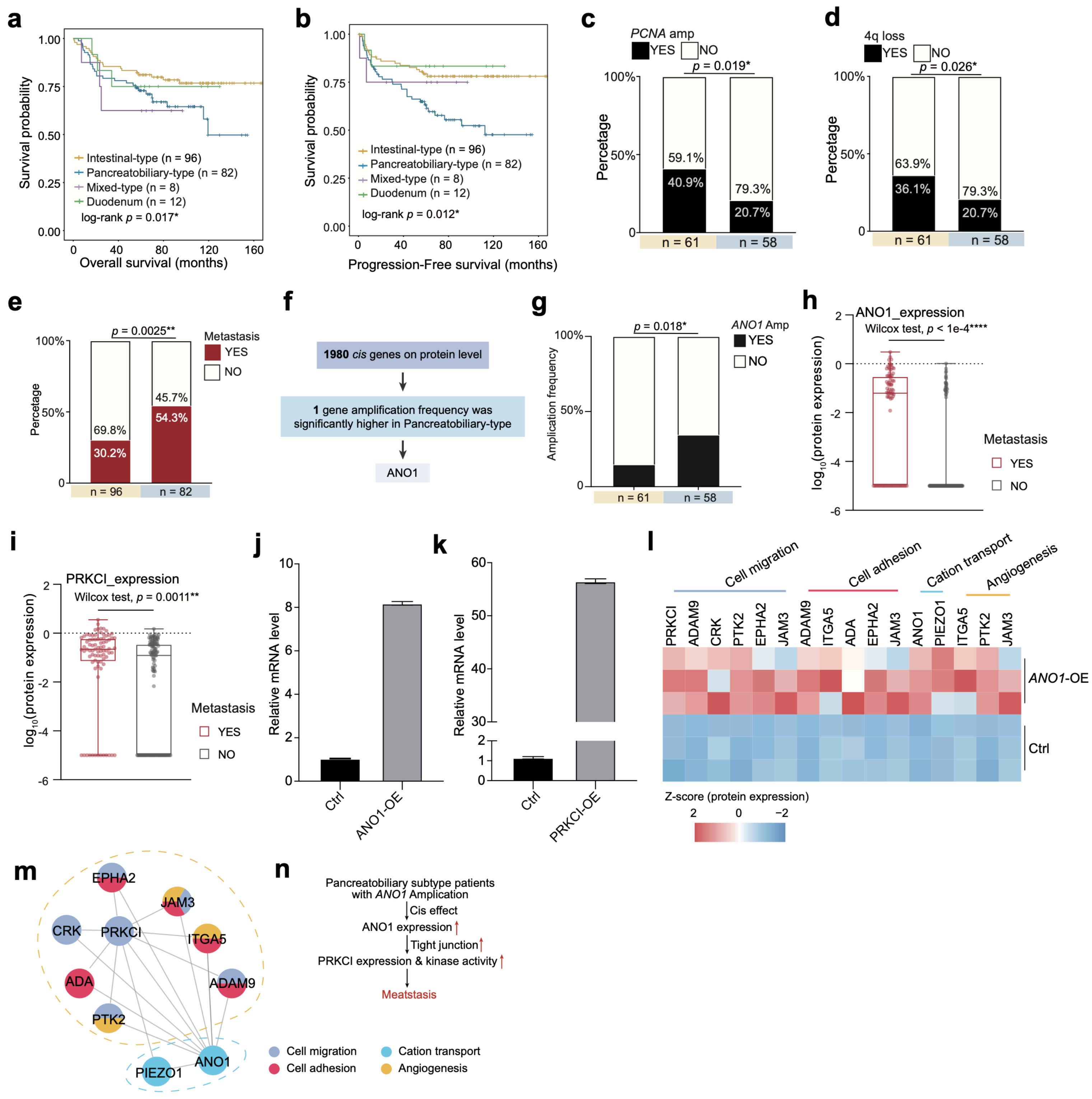

Supplementary Fig. S5 The differences between intestinal-type and pancreatobiliary-type related to Fig. 4 and Fig. 5

a. The Kaplan-Meier curve for overall survival based on pathology subtypes (log-rank test, $p = 0.017$).

b. The Kaplan-Meier curve for progression-free survival based on pathology subtypes (log-rank test, $p = 0.012$).

c. *PCNA* amplification status in intestinal-type (left column, yellow) and pancreatobiliary-type (right column, blue) (Fisher's exact test, $p = 0.019$).

d. 4q loss status in intestinal-type (left column, yellow) and pancreatobiliary-type (right column, blue) (Fisher's exact test, $p = 0.026$).

e. Metastasis status in intestinal-type (left column, yellow) and pancreatobiliary-type (right column, blue) (Fisher's exact test, $p = 0.0025$).

f. The Diagram illustrated the selection process of frequently occurred *cis* effect in the pancreatobiliary-type.

g. *ANO1* amplification status in intestinal-type (left column, yellow) and pancreatobiliary-type (right column, blue) (Fisher's exact test, $p = 0.018$).

h. Boxplot illustrated the ANO1 protein expression among the metastasis group (red) and no metastasis group (grey) (Wilcoxon rank-sum test, $p <$ 1e-4).

i. Boxplot illustrated the PRKCI protein expression among the metastasis group (red) and no metastasis group (grey) (Wilcoxon rank-sum test, $p =$ 1.1e-3).

j. Relative mRNA ANO1 abundance in SNU-478-*ANO1*-OE and control groups.

k. Relative mRNA PRKCI abundance in SNU-478-*PRKCI*-OE and control groups.

l. Heatmap of proteins interacted with ANO1 and these proteins associated pathways.

m. Interaction network constructed by the IP-MS results, proteins were colored by pathways (yellow, cell migration/adhesion, or angiogenesis; blue, cation transport).

n. A brief summary depicted the impact of ANO1 amplification in pancreatobiliary-type AMPAC.

Supplementary Fig. S6

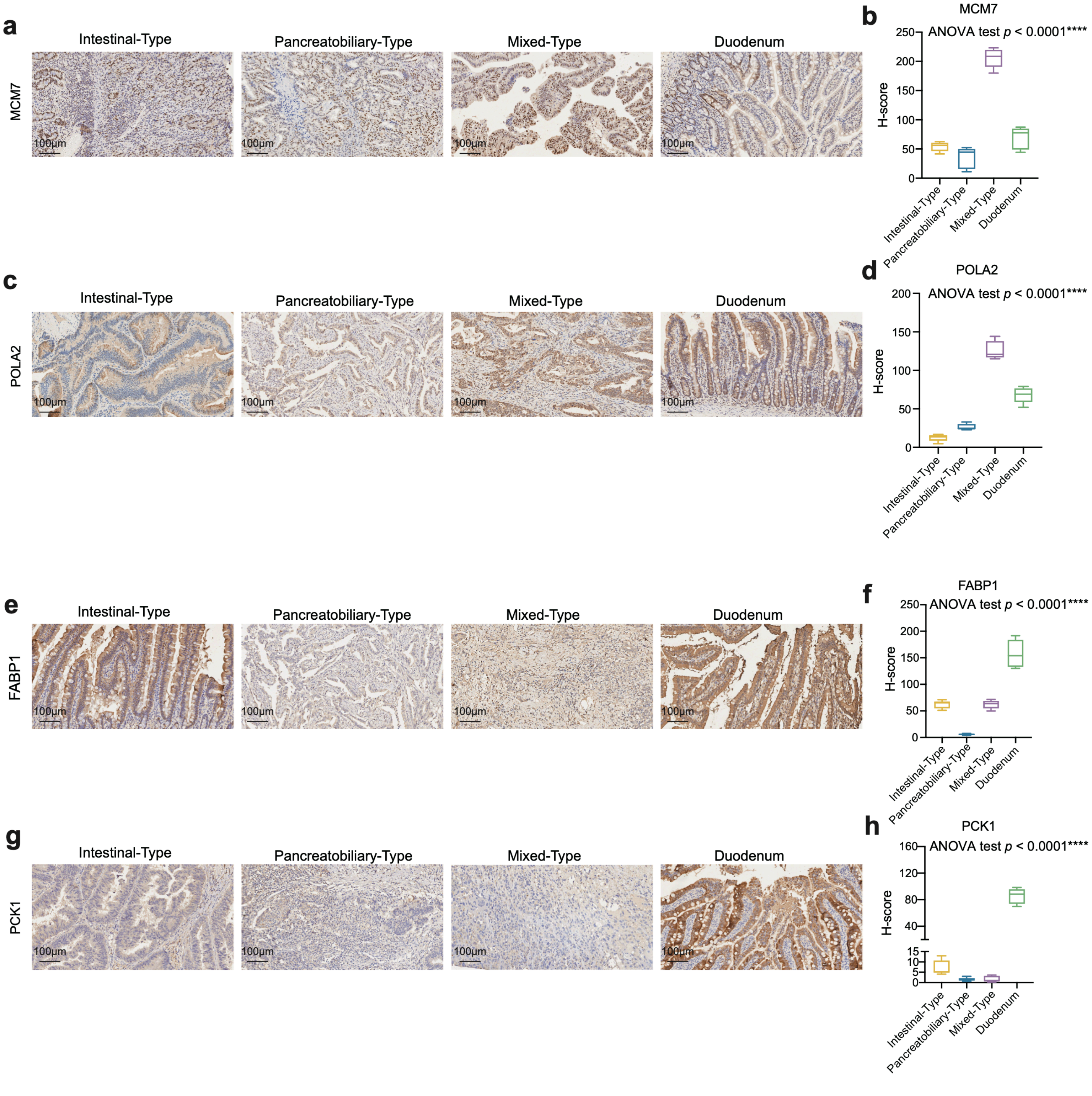

Supplementary Fig. S6 The IHC staining results related to Fig. 4

a. IHC staining images exhibited the expression of MCM7 among 4 pathological groups.

b. Boxplots exhibited the H-score of MCM7 IHC images.

c. IHC staining images exhibited the expression of POLA2 among 4 pathological groups.

d. Boxplots exhibited the H-score of POLA2 IHC images.

e. IHC staining images exhibited the expression of FABP1 among 4 pathological groups.

f. Boxplots exhibited the H-score of FABP1 IHC images.

g. IHC staining images exhibited the expression of PCK1 among 4 pathological groups.

h. Boxplots exhibited the H-score of PCK1 IHC images.

Supplementary Fig. S7

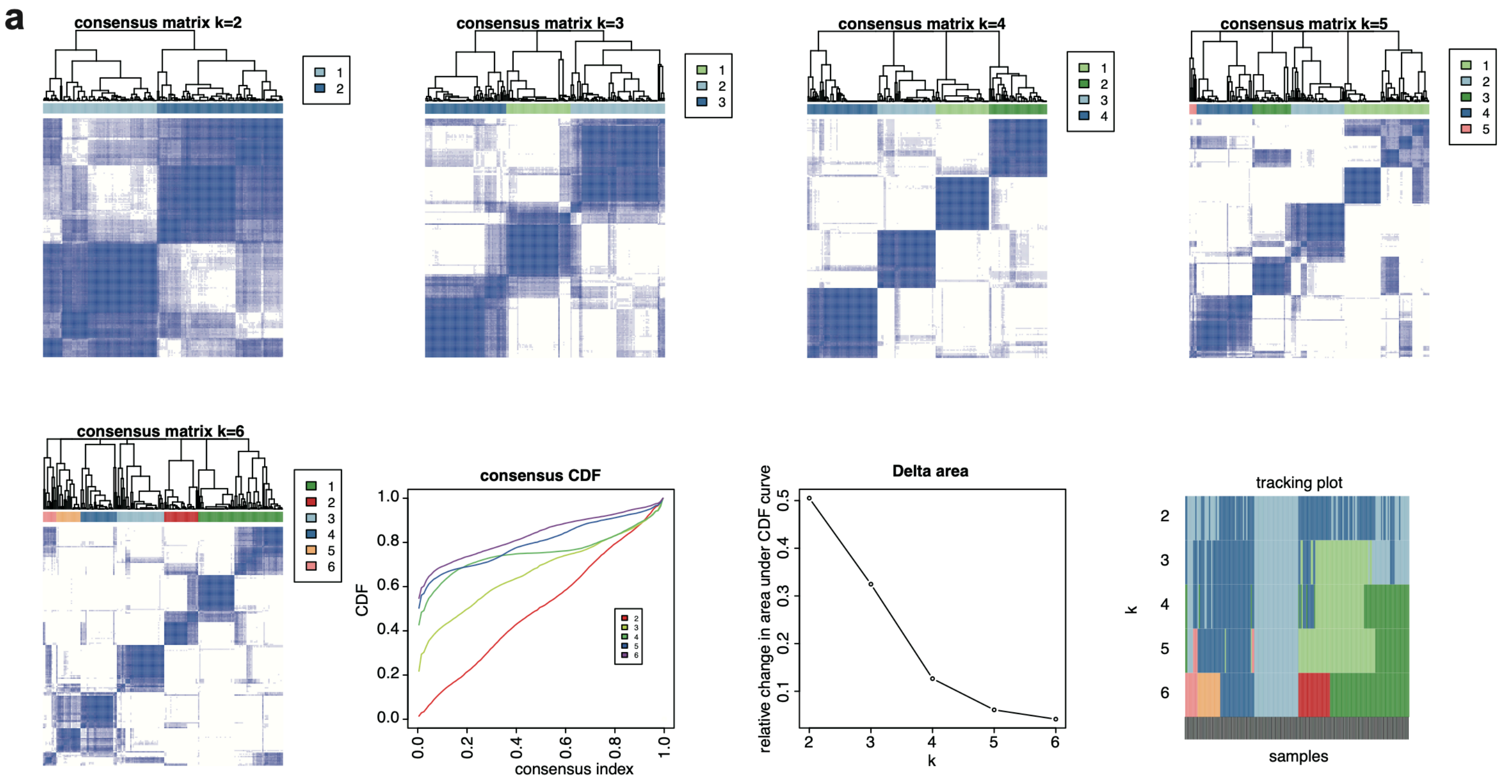

Supplementary Fig. S7 Proteomic clusters were identified based on proteomic data of AMPAC cohort (n = 198) by K-means consensus clustering upon their abundance. k was tested from 2 to 6 and consensus clustering was based on 1,000 resampled datasets. Consensus matrices, as well as consensus cumulative distribution function (CDF) plot, and delta area (change in CDF area) plot, are shown in order.

Supplementary Fig. S8

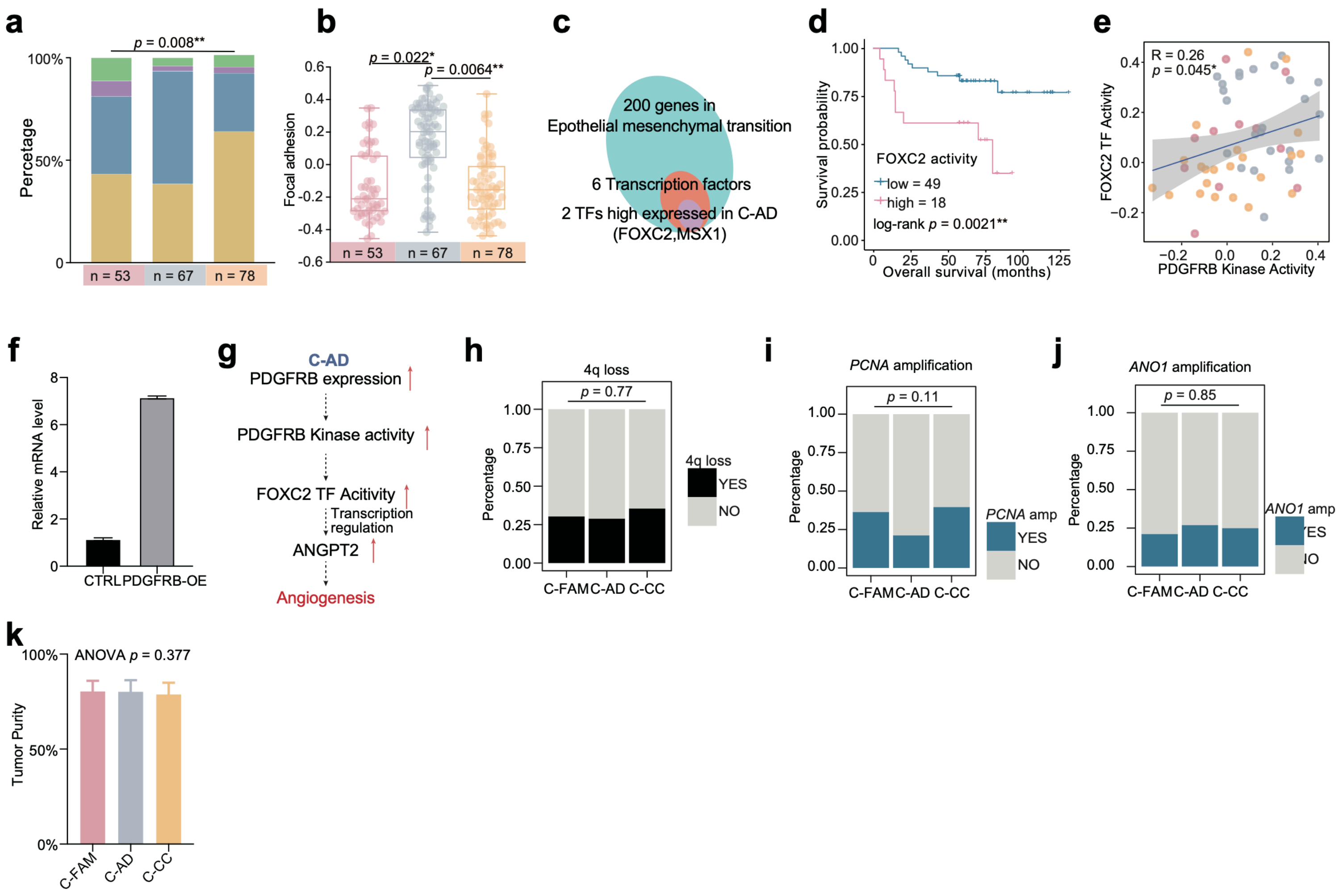

Supplementary Fig. S8 Proteomic cluster C-AD of AMPAC related to Fig. 6

a. Pathology subtypes in C-FAM (left column), C-AD (middle column), and C-CC (right column) (Chi-square test, $p = 0.008$).

b. Boxplot illustrated the C-FAM cluster has the highest GSVA score of fatty acid metabolism (Kruskal-Wallis test, C-FAM vs. C-AD: $p = 1.2e-4$, C-AD vs. C-CC: $p = 1.4e-4$).

c. Venn plot depicting activated TFs belonging to the epithelial mesenchymal transition pathway in C-AD.

d. The Kaplan-Meier curve for overall survival based on the FOXC2 TF activity (log-rank test, $p = 0.0021$).

e. Spearman correlation of PDGFRB kinase activity and FOXC2 TF activity (Spearman's r = 0.26, $p = 0.045$).

f. Relative mRNA abundance of PDGFRB in SNU-478 PDGFRB-OE and control groups.

g. Schematic representation of the mechanism of PDGFRB-induced angiogenesis in C-AD.

h. Chromosome 4q loss status in C-FAM (left box), C-AD (middle box), and C-CC (right box) (Fisher's exact test).

i. *PCNA* amplification status in C-FAM (left box), C-AD (middle box), and C-CC (right box) (Fisher's exact test).

j. *ANO1* amplification status in C-FAM (left box), C-AD (middle box), and C-CC (right box) (Fisher's exact test).

k. Barplot for tumor purity among three proteomic clusters.

Supplementary Fig. S9

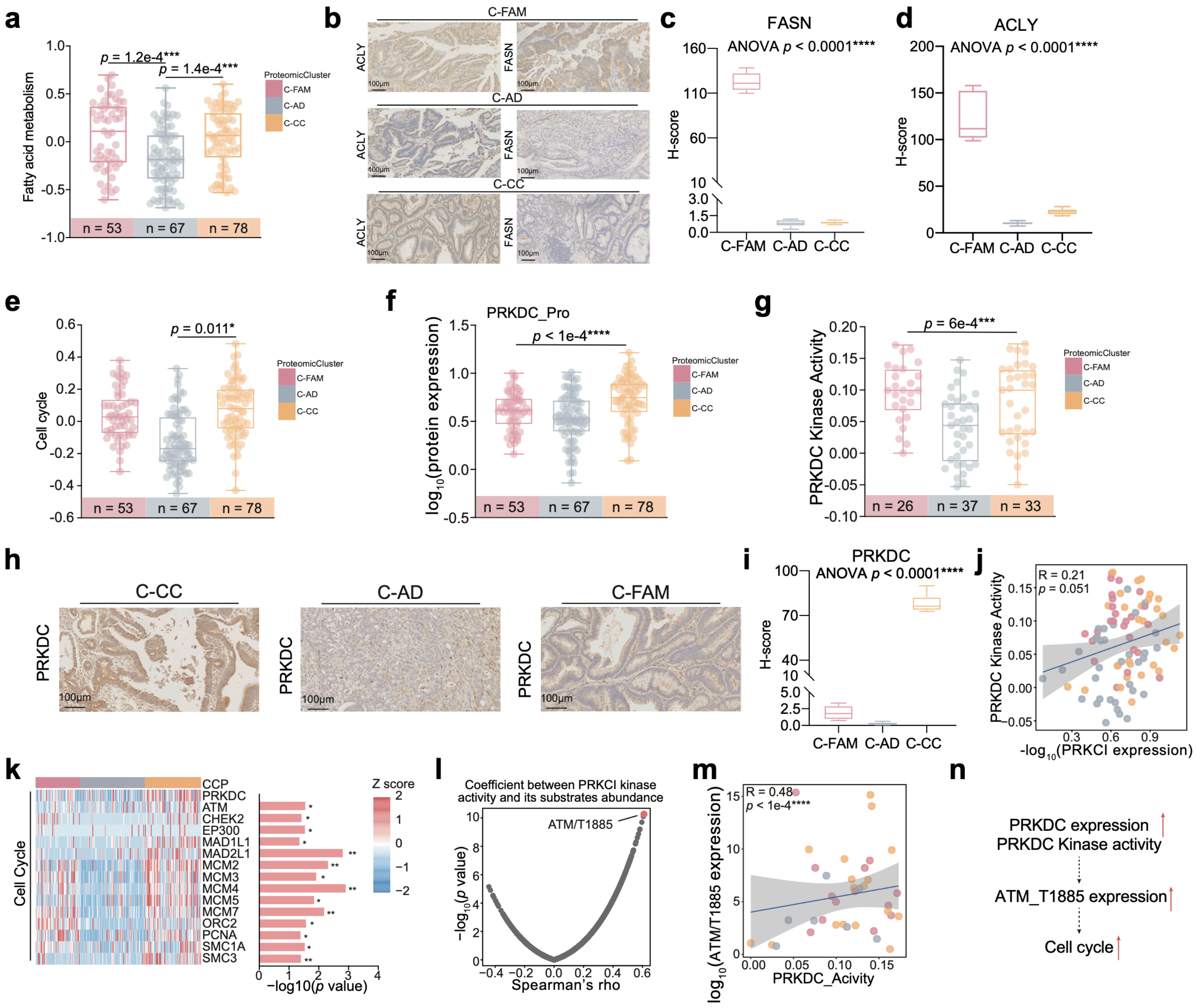

Supplementary Fig. S9 Proteomic clusters (C-FAM and C-CC) of AMPAC related to Fig. 6

a. Boxplot illustrated the C-FAM cluster has the highest GSVA score of the fatty acid metabolism pathway (Kruskal-Wallis test, C-FAM vs. C-AD: $p = 1.2e-4$, C-AD vs. C-CC: $p = 1.4e-4$).

b. IHC staining illustrated ACLY and FASN were highly expressed in the C-FAM compared to the other clusters (Scale bar = 100 μm).

c. Boxplot exhibited the H-score of FASN IHC images among C-FAM, C-AD, and C-CC (One-way ANOVA test, $p < 0.0001$).

d. Boxplot exhibited the H-score of ACLY IHC images among C-FAM, C-AD, and C-CC (One-way ANOVA test, $p < 0.0001$).

e. Boxplot illustrated the C-CC cluster has the highest GSVA score of the cell cycle pathway (Kruskal-Wallis test, C-AD vs. C-CC: $p = 0.011$).

f. Boxplot illustrated PRKDC protein expression among C-FAM (pink, left box), C-AD (gray, middle box), and C-CC (yellow, right box) (Kruskal-Wallis test, $p < 1e-4$).

g. Boxplot illustrated PRKDC kinase activity among C-FAM (pink, left box), C-AD (gray, middle box), and C-CC (yellow, right box) (Kruskal-Wallis test, $p = 6e-4$).

h. IHC staining illustrated PRKDC was highly expressed in the C-CC cluster (Scale bar = 100 μm).

i. Boxplot exhibited the H-score of PRKDC IHC images among C-FAM, C-AD, and C-CC (One-way ANOVA test, $p < 0.0001$).

j. Spearman correlation of PRKDC protein expression and PRKDC kinase activity (Spearman's r = 0.21, $p = 0.051$).

k. Heatmap for PRKDC and its substrates involved in cell cycle (left panel), and *p* values derived from Spearman correlation were shown on the right.

l. The volcano plot described the correlation between PRKDC kinase activity and the substrates (Spearman correlation).

m. Spearman correlation of PRKDC kinase activity and ATM/T1885 abundance (Spearman's r = 0.48, $p < 1e-4$).

n. The systematic diagram summarized the high level of PRKDC protein expression and

kinase activity that promote cell cycle.

Supplementary Fig. S10

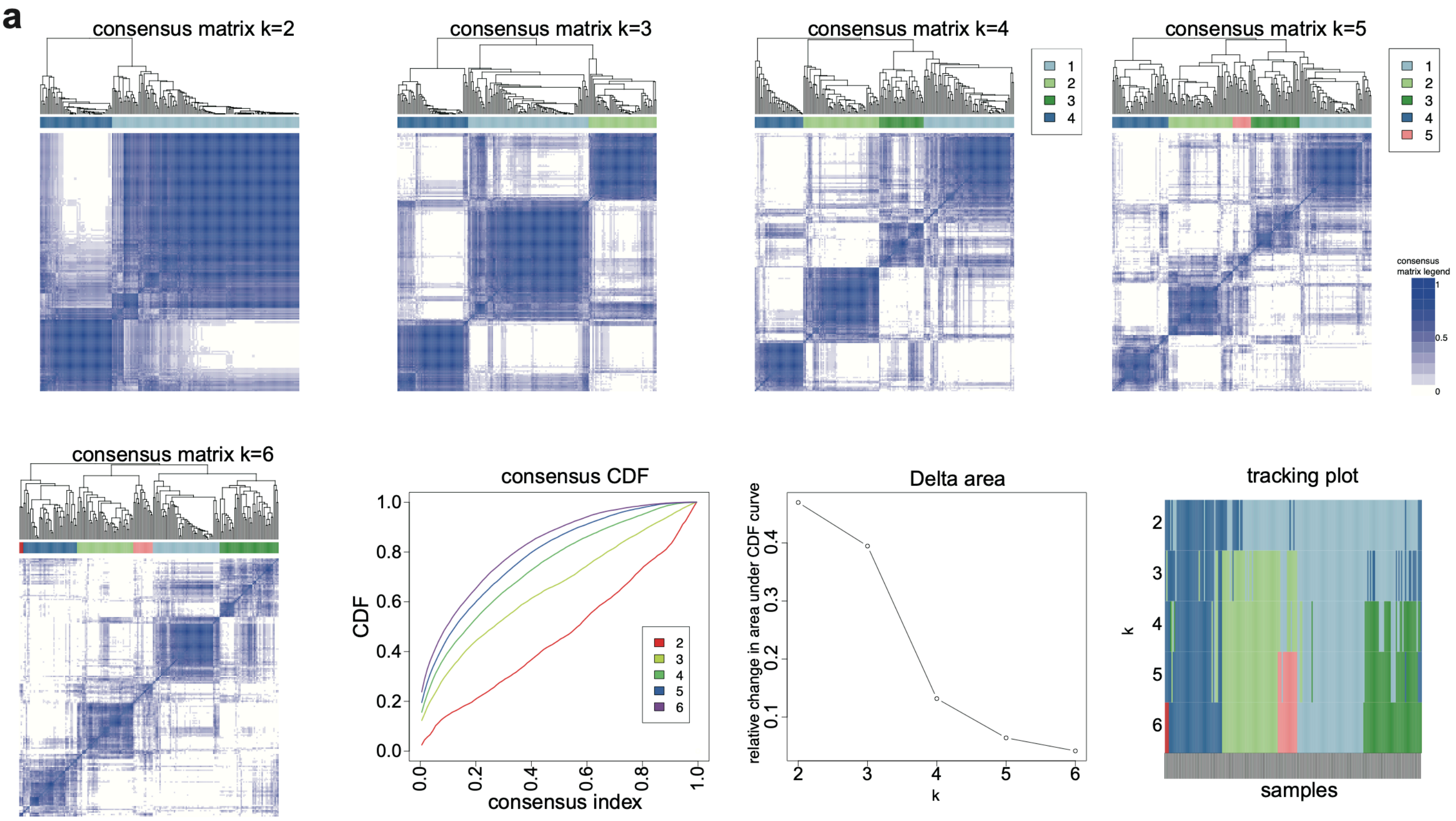

Supplementary Fig. S10 Immune clusters were identified based on xCell signatures of the AMPAC (n = 198) by K-means consensus clustering upon their abundance. k was tested from 2 to 6 and consensus clustering was based on 1,000 resampled datasets. Consensus matrices, as well as consensus cumulative distribution function (CDF) plot, and delta area (change in CDF area) plot, are shown in order.

Supplementary Fig. S11

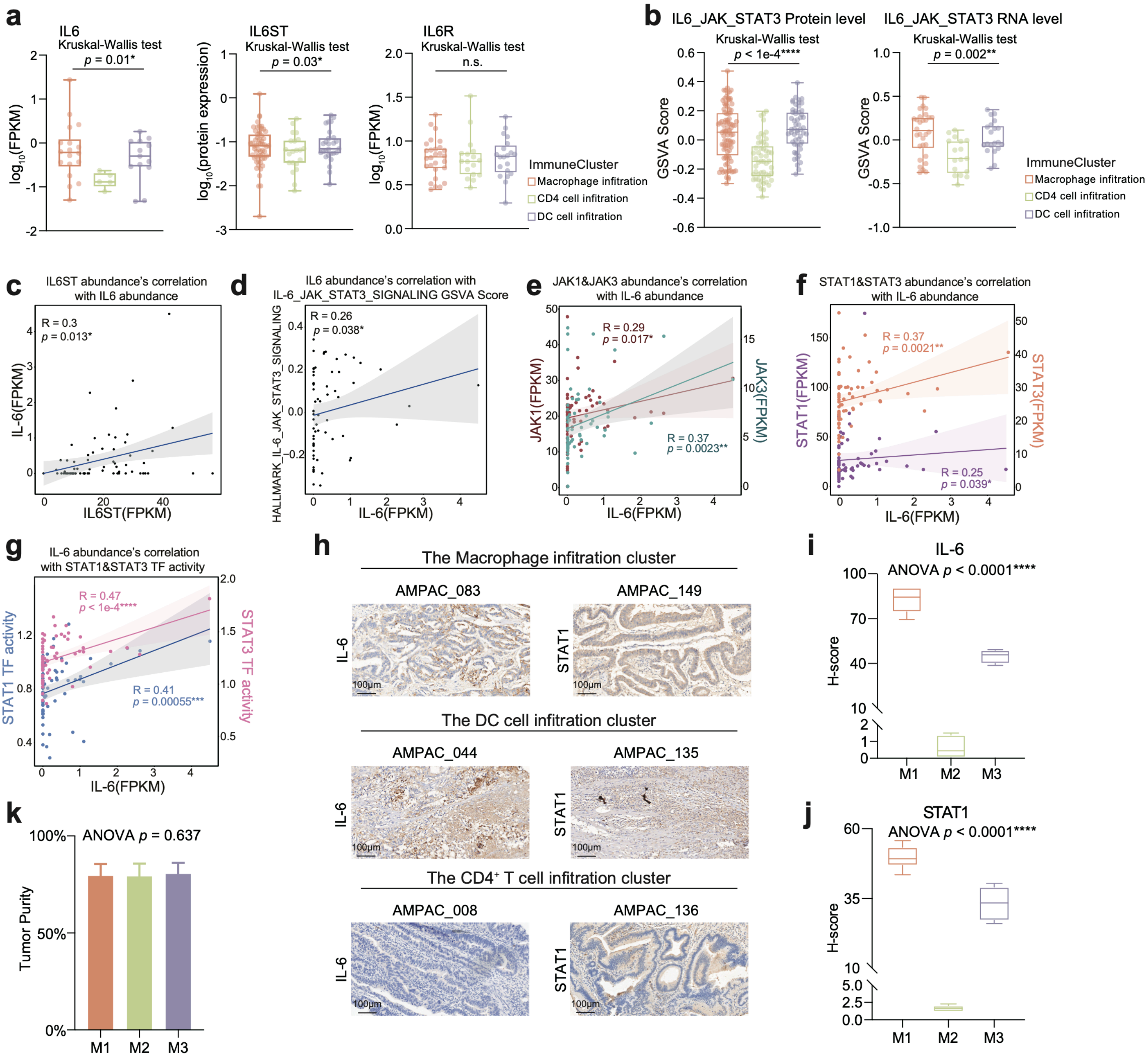

Supplementary Fig. S11 Immune clusters are identified based on proteomic data related to Fig. 7

a. Boxplot illustrated the IL-6 (left panel), IL6ST (middle panel), and IL6R (right panel) RNA expression among three immune clusters (Kruskal-Wallis test, IL-6: $p = 0.01$, IL6ST: $p = 0.03$, IL6R: n.s.).

b. Boxplot illustrated the IL-6/JAK/STAT3 GSVA score at protein level (left panel) and mRNA level (right panel) among three immune clusters (Kruskal-Wallis test, protein level: $p < 1e-4$, mRNA level: $p = 0.002$).

c. Spearman correlation of IL-6 FPKM and IL6ST RNA expression (Spearman's r = 0.3, $p = 0.013$).

d. Spearman correlation of IL-6 FPKM and IL-6/JAK/STAT3 GSVA score (Spearman's r = 0.26, $p = 0.038$).

e. Spearman correlation between IL-6 FPKM and JAK1 and JAK3 FPKM (IL-6 vs. JAK1: Spearman's r = 0.29, $p = 0.017$, IL-6 vs. JAK3: Spearman's r = 0.37, $p = 2.3e-3$).

f. Spearman correlation between IL-6 FPKM and STAT1 and STAT3 FPKM (IL-6 vs. STAT1: Spearman's r = 0.37, $p = 2.1e-3$, IL-6 vs. STAT3: Spearman's r = 0.25, $p = 0.039$).

g. Spearman correlation between IL-6 FPKM and STAT1 and STAT3 TF activity (IL-6 vs. STAT1: Spearman's r = 0.47, $p < 1e-4$, IL-6 vs. STAT3: Spearman's r = 0.41, $p = 5.5e-4$).

h. IHC staining illustrated IL-6 and STAT1 were highly expressed in the Macrophage infiltration cluster and the DC cell infiltration cluster (Scale bar = 100 μm).

i. Boxplot exhibited the H-score of IL-6 IHC images among M1, M2, and M3(One-way ANOVA test, $p < 0.0001$).

j. Boxplot exhibited the H-score of STAT1 IHC images among M1, M2, and M3(One-way ANOVA test, $p < 0.0001$).

k. Barplot for tumor purity among three immune clusters

Methods

Sample selection

The 186 ampullary adenocarcinoma and 12 duodenum adenocarcinoma formalin-fixed, paraffin-embedded (FFPE) tissues and paired NATs were acquired from Zhongshan Hospital, Fudan University from 2008 to 2017. All cases were collected regardless of histologic grade or surgical stage. Clinical information of these patients, including gender, age, drinking status, smoke status, vessel invasion, fascicular invasion, histological subtypes, TNM stages (AJCC cancer staging system 8th edition), cancer metastasis status, survival status, is listed in Supplementary Table S1a. All the patients received primary resection without any anti-cancer treatments prior to surgery. Postoperative surveillance and treatment were conducted consistently according to Zhongshan Hospital's guidelines. Each sample was assigned a new research ID, and the patient's name or medical record number used during hospitalization was deidentified.

Sample preparation

Formalin-fixed, paraffin-embedded (FFPE) specimens were prepared and provided by Zhongshan Hospital. One 3-μm-thick slide from FFPE blocks was sectioned for hematoxylin and eosin (H&E) staining. For genomic, proteomic, and phosphoproteomic sample preparation, 10-μm-thick slides were sectioned, deparaffinized with xylene and washed with gradient ethanol. Samples were just sectioned by 10-μm-thick slides without xylene deparaffinization nor gradient ethanol wash for RNA sample preparation. Selected specimens according to H&E staining were scraped, and materials were aliquoted and kept in storage at – 80 °C until further processing.

Pathology review

All samples were systematically evaluated to confirm the histopathological diagnosis and any variant histology according to the AJCC eighth edition 2017 staging system[35] by three expert pathologists. Additionally, all tumor samples were assessed for tumor content, the presence and extent of tumor necrosis, and signs of invasion into the muscularis propria. The samples used for multi-omics analysis were characterized with histologic tumor purity ranged from 70% to 90%. Tumor samples were also evaluated for the presence and extent of inflammatory infiltrates, as well as for the type of the infiltrating cells (lymphocytes, neutrophils, eosinophils, histiocytes, plasma

cells) in the tumor microenvironment. Any non-concordant diagnoses among the three pathologists were re-reviewed, and a resolution was reached following discussion.

The ABSOLUTE algorithm was utilized to evaluate the overall computational purity score for each sample. Computational tumor purity was inferred by R package ABSOLUTE[64] using WES data, respectively. The detailed was provided in **Supplementary Table S1c**. Tumor purity among 4 pathological groups, proteomic clusters and Immune clusters were shown in **Supplementary Fig. S1k**, **Supplementary Fig. S1l**, **Supplementary Fig. S8k** and **Supplementary Fig. S11k**, respectively.

DNA extraction

For the WES analysis, DNA from 133 FFPE specimens of AMPAC were extracted according to the manufacturer's instructions (QIAamp DNA Mini Kit; QIAGEN, Hilden, Germany). The isolated DNA quality and contamination were verified using the following methods:

(1) DNA degradation and contamination were monitored on 1% agarose gels.

(2) DNA concentration was measured via Qubit® DNA Assay Kit in Qubit® 2.0 Fluorometer (Invitrogen, CA, USA).

Library preparation

A total quantity of 0.6 μg genomic DNA per sample was used as the input material for DNA preparation. Sequencing libraries were generated using Agilent SureSelect Human All Exon Kit (Agilent Technologies, CA, USA) following the manufacturer's recommendations; further, index codes were added to each sample. Briefly, fragmentation was carried out by a hydrodynamic shearing system (Covaris, Massachusetts, USA) to generate 180–280 bp fragments. The remaining overhangs were converted into blunt ends via exonuclease/polymerase activity. Adapter oligonucleotides were ligated after adenylation of the 3′-ends of the DNA fragments. DNA fragments with ligated adapter molecules on both ends were selectively enriched via a polymerase chain reaction (PCR). Thereafter, libraries were hybridized with the liquid phase of biotin-labeled probes, and magnetic beads with streptomycin were used to capture the exons of genes. Captured libraries were enriched in another PCR reaction to add index tags to prepare them for sequencing. Finally, the products were purified using AMPure XP system (Beckman Coulter, Beverly, USA) and quantified using an Agilent high sensitivity DNA assay (Agilent)

on an Agilent Bioanalyzer 2100 system (Agilent Technologies, CA, USA).

Clustering and DNA Sequencing

The clustering of the index-coded samples was performed on a cBot Cluster Generation System using a HiSeq PE Cluster Kit (Illumina) according to the manufacturer's instructions. After cluster generation, the DNA libraries were sequenced on Illumina NovaSeq 6000 platform, and 150 bp paired-end reads were generated.

Whole-exome sequencing Quality control

The original fluorescence image files obtained from Novaseq platform are transformed into short reads (Raw data) by base calling and these short reads are recorded in FASTQ format, which contains sequence information and corresponding sequencing quality information. Sequence artifacts, including reads containing adapter contamination, low-quality nucleotides, and unrecognizable nucleotides[65], undoubtedly set the barrier for the subsequent reliable bioinformatics analysis. Hence quality control is an essential step applied to guarantee meaningful downstream analysis. The steps of data processing were as follows:

(1). Discard the paired reads if either one read contains adapter contamination (>10 nucleotides aligned to the adapter, allowing ≤10% minimasmatches).

(2). Discard the paired reads if more than 10% of bases are uncertain in either one read.

(3). Discard the paired reads if the proportion of low quality (Phred quality <5) bases is over 50% in either one read.

All the downstream bioinformatics analyses were based on the high-quality clean data, which were retained after these steps. At the same time, QC statistics including total reads number, raw data, raw depth, sequencing error rate, percentage of reads with Q30 (the percent of bases with phred-scaled quality scores >30), and GC content distribution were calculated and summarized. WES was conducted with mean coverage depths of 108X for tumor samples and 118X for adjacent non-tumor brain samples, which is consistent with the recommendations for WES.

Reads mapping to reference sequence

Valid sequencing data were mapped to the reference human genome (UCSC hg19) using Burrows–

Wheeler aligner (BWA) software to obtain the original mapping results stored in BAM format[66]. If one read, or one paired read, was mapped to multiple positions, the strategy adopted by the BWA was to choose the most likely placement. If two or more most likely placements were present, the BWA picked one randomly. Then, SAMtools[67] and Picard (http://broadinstitute.github.io/picard/) were used to sort BAM files and perform duplicate marking, local realignment, and base quality recalibration to generate final BAM files for computation of the sequence coverage and depth. The mapping step was very difficult due to mismatches, including true mutations and sequencing errors, and duplicates resulting from PCR amplification. These duplicate reads were uninformative and should not be considered as evidence for variants. We used Picard to mark these duplicates for the follow-up analysis.

Variant calling

Samtools mpileup and bcftools were used to perform variant calling and identify SNPs and InDels. Somatic SNP variant calls were assessed using MuTect[68], and the Indels variant calls were assessed using Strelka[69] with default options. The resulting somatic mutations were annotated using the ANOVAR RefSeq gene-based annotation.

Copy number analysis

Copy number varivations (CNVs) were called by following the somatic CNV calling pipeline in GATK's (GATK 4) Best Practice. The results of this pipeline and segment files of every 1000 were input in GISTIC2[70], to identify significantly amplified or deleted focal-level and armlevel events, with a Q value < 0.1 considered significant. A log2 ratio cutoff 1 was used to define SCNA amplification and deletion. We further summarize the arm-level copy number change based on a weighted sum approach[15], in which the segment-level log2 copy ratios for all the segments located in the given arm were added up with the length of each segment being weighted. To exclude false positives as much as possible, relatively stringent cutoff thresholds were used with the following parameters: -ta 0.1 -tb 0.1 -brlen 0.98 -conf 0.9. Other parameters were the same as default values.

Co-occurrence and mutual exclusivity analysis of mutations

Co-occurrence and mutually exclusive mutated genes were detected using Fisher's exact test in order to determine the co-occurrence and mutually exclusively of significantly mutated genes in our mutational dataset.

Analysis of significantly mutated genes

Filtered mutations (including SNV and indel) were further used to identify significantly mutated genes by MutSigCV (https://software.broadinstitute.org/cancer/cga/mutsig, version 1.41) with default parameters. Final MutSigCV p values were converted to q values using the method of Benjamini and Hochberg[71], and genes with $q \leq 0.1$ were declared to be significantly mutated.

Mutation frequency in the Fudan cohort and previous AMPAC studies

Mutation frequencies for previous AMPAC studies were obtained from their supplementary materials[9,10]. The frequencies of all genes were compared with those from the Fudan cohort using Fisher's exact test.

Mutational signature analysis using the Sigminer approach

Mutation signatures were jointly inferred for 133 tumors using the R package sigminer[72]. The sigminer approach (https://github.com/ShixiangWang/sigminer) was used to extract the underlying mutational signatures. The 96 mutation vectors (or contexts) generated by somatic SNVs based on six base substitutions (C>A, C>G, C>T, T>A, T>C, and T>G) within 16 possible combinations of neighboring bases for each substitution were used as input data to infer their contributions to the observed mutations. Sigminer using a nonnegative matrix factorization (NMF) approach was applied to decipher the 96 × 133 (i.e., mutational context-by-sample) matrix for the 30 known COSMIC cancer signatures (https://cancer.sanger.ac.uk/cosmic/signatures) and infer their exposure contributions.

Tumor mutational burden

Tumor mutational burden (TMB) was defined as the number of somatic mutations (including base substitutions and indels) in the coding region. Synonymous alterations were also counted[73]. To

calculate the TMB, the total number of mutations counted was divided by the size of the coding sequence region of the Agilent SureSelect Human All Exon V6.

RNA extraction

RNA was extracted from tissues by using TIANGEN® RNAprep Pure FFPE Kit (#DP439) according to the reagent protocols. For library preparation of RNA sequencing, a total amount of 500 ng RNA per sample was used as the input material for the RNA sample preparations. Sequencing libraries were generated using Ribo-off® rRNA Depletion Kit (H/M/R) (Vazyme #N406) and VAHTS® Universal V6 RNA-seq Library Prep Kit for Illumina (#N401-NR604) following the manufacturer's recommendations, and index codes were added to attribute sequences to each sample. The libraries were sequenced on an Illumina platform and 150 bp pairedend reads were generated.

RNA-Seq data analysis

RNA-seq raw data quality was assessed using FastQC (v0.11.9), and the adaptor was trimmed with Trim_ Galore (version 0.6.6) before any data filtering criteria were applied. Reads were mapped onto the human reference genome (UCSC hg19) using STAR software (v2.7.7a). The mapped reads were assembled into transcripts or genes by using StringTie software (v2.1.4) and the genome annotation file (version hg19). For quantification purpose, the relative abundance of the transcript/gene was measured using the normalized metrics, FPKM (fragments per kilobase of transcript per million mapped reads). Transcripts with an FPKM score above one were retained, resulting in a total of 19,193 gene IDs. All known exons in the annotated files were 100% covered.

Protein extraction and tryptic digestion

Lysis buffer [0.1 M Tris–HCl (pH 8.0), 0.1 M DTT (Sigma, 43,815), 1 mM PMSF (Amresco, M145)] was added to the extracted tissues and subsequently sonicated for 1min (3 s on and 3 s off, amplitude 25%) on ice. The supernatants were collected, and the extracted tissues were then lysed with 4% sodium dodecyl sulfate (SDS) and kept for 2 h at 99 °C with shaking at 1500 rpm. The solution was collected by centrifugation at 12,000 × g for 5 min. A fourfold volume of acetone was added to the supernatant and kept in -20 °C for a minimum of 4 h. Subsequently, the acetone-precipitated proteins

were washed three times with cooled acetone and then pumped out using the Concentrator plus (Eppendorf, Germany). Filter-aided sample preparation (FASP) procedure was used for protein digestion[74]. The proteins were resuspended in 200 μL 8 M urea (pH 8.0) and loaded twice in 30 kD Microcon filter tubes (Sartorius) and centrifuged at 12,000g for 20 min. The precipitate in the filter was washed twice by adding 200 μL 50 mM NH4HCO3. The precipitate was resuspended in 50 μL 50 mM NH4HCO3. Protein samples underwent trypsin digestion (enzyme-to-substrate ratio of 1:50 at 37 °C for 18–20 h) in the filter and then were collected by centrifugation at 12,000 g for 15 min. Additional washing, twice with 200 μL of MS water, was essential to obtain greater yields. Finally, the centrifugate was dried by using the Concentrator plus (Eppendorf, Germany) for sub-sequential MS analysis.

Phosphopeptide enrichment

For the phosphoproteomic analysis, peptides were extracted from the FFPE slides after trypsin digestion using the methods described above. The tryptic peptides were then enriched with High-Select™ Fe-NTA Phosphopeptide Enrichment Kit (Thermo Scientific cat. A32992), following the manufacturer's recommendation. Briefly, peptides were suspended with binding/wash buffer (contained in the enrichment kit), mixed with the equilibrated resins, and incubated at 21–25 °C for 30 min. After incubation, the resins were washed thrice with binding/wash buffer and twice with water. The enriched peptides were eluted with elution buffer (contained in the enrichment kit) and dried in a Concentrator plus (Eppendorf, Germany).

LC-MS/MS analysis

LC-MS/MS were performed on Easy-nLC liquid chromatography system (Thermo Scientific) coupled to an Orbitrap Fusion Lumos Tribrid platform with FAIMS (Thermo Fisher Scientific).

The peptides were dissolved with 10 μl loading buffer (5% methanol and 0.2% formic acid), and 5 μl was loaded onto a 360 μm I.D. × 2 cm, C18 trap column at a maximum pressure 280 bar with 12 μl solvent A (0.1% formic acid in water). Peptides were separated on 150 μm I.D. × 30 cm column (C18, 1.9 μm, 120 Å, Dr. Maisch GmbH) with a linear 5–35% Mobile Phase B (ACN and 0.1% formic acid) at 600 nl/min for 150 mins. FAIMS separations were performed with the following settings: inner electrode temperature = 100 °C (except where noted), outer electrode temperature =

100 °C, FAIMS carrier gas flow = 2.3 L/min. The dispersion voltage (DV) was set at−5000 V, and the compensation voltage was stepped into 40 V, 55 V and 70 V.

These analyses utilized a 120 000 resolving power survey scan with AGC = 3 000 000, followed by MS/MS of the most intense precursors for 80 ms. The MS/MS analyses were performed by1.6 m/z isolation with the quadrupole, normalized HCD (higher-energy collisional dissociation) collision energy of 27%, and analysis of fragment ions in the ion trap using the "Turbo" speed scanning from 200 to 1200 m/z. Dynamic exclusion was set to 12 s. Monoisotopic precursor selection (MIPS) was set to Peptide, maximum injection time was set to 20 ms, AGC target was set to 20 000, and charge states unknown, +1, or >+5 were excluded and the advanced peak determination was toggled on.

For the phosphoproteomic analysis, the phosphopeptides were analyzed on FAIMS interfaced Orbitrap Fusion Lumos Tribrid Mass Spectrometer (Thermo Fisher Scientific, Rockford, IL, USA) equipped with an Easy nLC-1000 (Thermo Fisher Scientific, Rockford, IL, USA) and a Nanoflex source (Thermo Fisher Scientific, Rockford, IL, USA). Dried peptide samples re-dissolved in buffer A (0.1% FA in water) were loaded to a 2 cm self-packed trap column using buffer A and separated on a 150 μm inner diameter column with a length of 30 cm over a 150 min gradient (buffer A: 0.1% FA in water; buffer B: 0.1% FA in 80% ACN) at a constant flow rate of 600 nL/min (0–150 min, 0 min, 4% B; 0–10 min, 4–15% B; 10–125 min, 15–30% B; 125–140 min, 30–50% B; 140–141 min, 50–100% B; 141–150 min, 100% B). The eluted phosphopeptides were ionized and detected. Compensation Voltages (CV) among -30 V, -60 V, and -120 V were interrogated to find precursor rich CVs. Mass spectra were acquired over the scan range of m/z 350–1500 at a resolution of 120,000 (AUG target value of 5E5 and max injection time 50 ms). For the MS2 scan, the higher-energy collision dissociation fragmentation was performed at a normalized collision energy of 30%. The MS2 AGC target was set to 1e4 with a maximum injection time of 10 ms, peptide mode was selected for monoisotopic precursor scan, and charge state screening was enabled to reject unassigned 1 +, 7 +, 8 +, and > 8 + ions with a dynamic exclusion time of 45 s to discriminate against previously analyzed ions between ± 10 ppm.

metabolites extraction

Lipid extraction MS water (200 mL) and methanol (240 mL) was added to a sample aliquot, and the tube was vortexed. After grinding beads was added to each tube, the grinding tube was

placed in the precooled adapter, the frequency of the grinding instrument was set to be 60Hz, the grinding operation to be 15s, the grinding interruption to be 5s, and the grinding operation times to be 10 min. Then, 800 mL of methyl tert-butyl ether [MTBE] was added, and the mixture was placed in the ultrasonic cleaner for ice bath ultrasonic for 30 min. After the mixture was centrifuged at 14,000 g for 10 min, the upper (organic) phase was collected and dried.

LC-MS/MS analysis of metabolites

LC-MS/MS of lipids were performed on Easy-nLC liquid chromatography system (Thermo Scientific) coupled to Q Exactive HFX platform (Thermo Fisher Scientific). A 2.1 mm I.D. 3 100 mm column (Waters, Acclaim C30) was balanced with 70% solvent A (10 mM ammonium formate and 60% ACN in water). The lipids were dissolved with 10 mL loading buffer (50% isopropyl alcohol [IPA] and 50% ACN), and 5 mL was loaded onto a 2.1 mm I.D. 3 100 mm column (Waters, Acclaim C30) at 0.26 mL/min. Lipids were separated with a linear 30–100% Mobile Phase B (10 mM ammonium formate and 0.1% formic acid, 90% IPA in ACN) for 20 min. These analyses utilized a 120,000 resolving power survey scan with AGC = 1 000 000, followed by MS/MS of the most intense precursors for 80 ms. The MS/MS analyses were performed by1.5 m/z isolation with the quadrupole, normalized HCD (higher-energy collisional dissociation) collision energy of 20%, 40%, and 60% and analysis of fragment ions in the ion trap scanning from 200 to 2000 m/z. Maximum injection time was set to 20 ms, AGC target was set to 200,000.

Metabolites identification and quantification

Metabolite profiles were analyzed by LipidSearch 4.2 (Thermo Fisher Scientific, CA, USA), a leading commercial lipidomics software platform[75]. The target database was Q Exactive HFX and the peak detection was recalc isotope. The search options were as follows: parent tolerance, 5 ppm, product tolerance, 8 ppm; m-score threshold, 2; Quan m/z tolerance ±5 ppm; Quan retention time (RT) range ±0.5 min; use of main isomer filter and for the ID quality filter, A-B; adduct ions, H+ and NH4 + for positive ion mode and H- and HCOO- for negative ion mode. Lipid alignments were performed with below parameters: ExpType, LC-MS; Alignment method, Mean; R. T. Tolerance, 2; Calculate unassigned peak area, on; Filter Type, New Filter; Toprank Filter, on; Main Node Filter, Main isomer peaks; m-Score Threshold, 5.0; ID quality

filter: A, B, C and D. The results were extracted using LipidSearch 4.1.3 software. Finally, the lipids were manually filtered according to the following rules:

(1) The peak areas of lipids with m-Score <10 were revised to 0;

(2): The peak areas of the lipids with AreaScore < 0.7 were revised to 0;

(3): The peak areas of the lipids with PeakQuality < 0.9 were revised to 0;

(4): The peak areas of the lipids with Occupy < 5 were revised to 0;

(5): The peak areas of the lipids with Grade C and D were revised to 0.

MS database searching Peptide and protein identification

MS raw files were processed with a "Firmiana" (a onestop proteomic cloud platform)[76] against the human National Center for Biotechnology Information (NCBI) RefSeq protein database using Mascot 2.4 (Matrix Science Inc., London, UK). The maximum number of missed cleavages was set to two. Mass tolerances of 10 ppm for the precursor and 10 ppm for production were allowed. The fixed modification was cysteine carbamidomethylation, while the variable modifications were N-acetylation and methionine oxidation. For the quality control of protein identification, the targetdecoy-based strategy was applied to confirm that the false discovery rate (FDR) of both peptides and proteins was lower than 1%. The program percolator was used to obtain the probability value (q value) and showed that the FDR (measured by the decoy hits) of every peptide–spectrum match (PSM) was lower than 1%. All peptides shorter than seven amino acids were removed. The cutoff ion score for peptide identification was set at 20. All PSMs in all fractions were combined for protein quality control, which was a stringent quality control strategy. The q values of both target and decoy peptide sequences were dynamically increased employing the parsimony principle until the corresponding protein FDR was less than 1%. Finally, to reduce the false positive rate, proteins with at least two unique peptides were selected for further investigation.

Phosphoproteome MS raw files were searched against the human RefSeq protein database using Proteome Discoverer (version 2.3.0.523) with a Mascot[77] (version 2.3.01) engine with a percolator[78]. Carbamidomethyl cysteine was used as a fixed modification, and oxidized methionine, protein N-term acetylation, and phospho (S/T/Y) were set as variable modifications. The false discovery rate (FDR) of peptides and proteins was set at 1%. The tolerance for spectral searches a mass tolerance of 20 ppm for the precursor. The maximum number of missing cleavage site was set at 2. For

phosphosite localization, ptmRS[79] was used to determine phosphosite confidence and a phosphosite probability > 0.75 was used for further analysis.

Label-free-based MS quantification of proteins

The one-stop proteomic cloud platform, "Firmiana," was further employed for protein quantification. The identification results and the raw data from the mzXML files were loaded. Then, for each identified peptide, the extracted-ion chromatogram (XIC) was extracted by searching against MS1 based on its identification information, and the abundance was estimated by calculating the area under the extracted XIC curve. For protein abundance calculation, the non-redundant peptide list was used to assemble proteins following the parsimony principle. Protein abundance was then estimated by a traditional label-free, intensity-based absolute quantification (iBAQ) algorithm, which divided protein abundance (derived from identified peptide intensities) by the number of theoretically observable peptides[80,81]. The fraction of total (FOT), a relative quantification value that was defined as a protein's iBAQ divided by the total iBAQ of all identified proteins in one experiment, was calculated as the normalized abundance of a particular protein in the experiments. Finally, the FOT was further multiplied by 1e6 for the ease of presentation, and NA values were replaced with 1e−5toadjust extremely small values.

For the phosphoproteomic data, the intensities of the phosphopeptides were extracted from the Proteome Discover (version 2.3). For the phosphoprotein abundance calculation, the non-redundant phosphor-peptide list was used to assemble the proteins by following the parsimony principle. Next, the phosphoprotein abundance was estimated by a traditional label-free, iBAQ algorithm, which divided the protein abundance (derived from the intensities of the identified peptides) by the number of theoretically observable peptides[81]. For phosphosite localization, the ptmRS[79] was used to determine phosphosite confidence and phosphosite probability > 0.75 is considered as confident phosphosites.

Quality control of the mass spectrometry data

For the quality control of the performance of mass spectrometry, the HEK293T cell (National Infrastructure Cell Line Resource) lysate was measured every three days as the quality control standard. The quality control standard was digested and analyzed using the same method and

conditions. A pairwise Spearman's correlation coefficient was calculated for all quality control runs in the statistical analysis environment R (version 4.0.2). The average correlation coefficient among the standards was 0.9, demonstrating the consistent stability of the mass spectrometry platform.

Kinase activity prediction

To estimate changes in kinase activity, we performed kinase enrichment analysis on significantly differentiated phosphosites in tumors compared to NATs, for intestinal-type and pancreatobiliary-type or each subtype via kinase–substrate enrichment analysis (KSEA)[82]. Known kinase–substrate site relationships from PhosphoSitePlus (PSP)[83]or NetworKIN 3.0[84] with scores greater than 1 were used for kinase–substrate analysis. A kinase score was given for each kinase based exclusively on the collective phosphorylation status of its substrates and transformed into a z-score. For kinase enrichment analysis, the threshold used for significantly enriched kinases was $p < 0.05$.

Missing value imputation

For the proteomic and phosphoproteomic data, FOTs multiplied by 1E5 were used for quantification, and missing values were imputed with 1E−5 and finally, log2-transformed, if necessary.

Differential protein analysis

Differential protein analysis Student t-test was used to examine whether proteins were differentially expressed between the tumors and NATs. Upregulated or downregulated proteins in tumors were defined as proteins differentially expressed in tumors compared with NATs (T/NAT > 2 or < 1/2, Wilcoxon rank-sum test, Benjamini-Hochberg adjusted $p < 0.05$). Kruskal-Wallis test was used to examine whether proteins were differentially expressed between patients with different histological subtypes (Benjamini-Hochberg adjusted $p < 0.05$). Kruskal-Wallis test was used to examine whether proteins were differentially expressed among three proteomic subtypes and immune subtypes (Benjamini-Hochberg adjusted $p < 0.05$).

Pathway enrichment analysis

Differentially expressed genes were subjected to gene ontology and KEGG pathway enrichment analysis in DAVID[85] with a p value/FDR < 0.1. We used gene sets of molecular pathways from the KEGG[86]/Hallmark[87]/Reactome[88]/GO[89] databases to compute pathways.

Pathway scores and correlation analysis

Single-sample gene set enrichment analysis (ssGSEA)[90] was utilized to obtain pathway scores for each sample based on RNA-seq, proteomic, and phosphoproteomic data using the R package GSVA[91]. Correlations between the pathway scores and other features were determined using Spearman's correlation. Inferred activity was performed using ssGSEA implemented in the R package GSVA with a minimum gene set size of 10. The transcriptional targets of transcription factors (TF) mentioned in this work were collected from the ENCODE Project Consortium[92] and used to infer TF activity via ssGSEA.

Cell cycle analysis

Multi-gene proliferation scores (MGPSs) were calculated as the mean expression level of all cell cycle-regulated genes in each sample as described previously[93,94]. Briefly, MGPS was calculated from the mean normalized proteomic data in each sample in our study.

Phosphopeptide analysis-kinase and substrate regulation

KSEA algorithm was used to estimate the kinase activities based on the abundance of phosphosites. Kinasesubstrate enrichment analysis (KSEA) estimates changes in a kinase's activity by measuring and averaging the amounts of its identified substrates instead of a single substrate, which enhances the signal-to-noise ratio from inherently noisy phosphoproteomic data[82,95]. If the same phosphorylation motif was shared by multiple kinases, it was used for estimating the activities of all known kinases. The use of all curated substrate sequences of a particular kinase minimizes the overlapping effects from other kinases and thus improves the precise measurement of kinase activities. The information of kinase-substrate relationships was obtained from publicly available databases including PhosphoSite[38], Phospho.ELM[36], and PhosphoPOINT[37]. The information of substrate motifs was obtained either from the studies[96] or from an analysis of KSEA dataset with Motif-X[82].

Protein–protein interaction network construction

Interaction network among the proteins and phosphorylated proteins was generated with STRING v 11.0 (https://string-db.org/) using medium confidence (0.4), and experiments and database as the active interaction sources. The network was visualized using Cytoscape version 3.8.0[97].

Consensus clustering analysis

The protein expression matrix of the 198 tumor samples was used to identify the proteomic clusters using the consensus cluster method. Consensus clustering was performed using the ConsensusClusterPlus (R package ConsensusClusterPlus v.1.48.0)[98,99], with the top 50% most varied protein. The following detail settings were used for clustering: number of repetitions = 10,000 bootstraps; pItem = 0.8 (resampling 80% of any sample); pFeature= 1 (resampling 100% of any protein); and clusterAlg = "pam"; and distance = "spearman. The number of clustering was determined by three factors, the average pairwise consensus matrix within consensus clusters, the delta plot of the relative change in the area under the cumulative distribution function (CDF) curve, and the average silhouette distance for consensus clusters. The consensus matrices for k = 2, 3, 4, and 5 clusters are shown in **Supplementary Fig. S7a**. A consensus matrix with k = 3 appeared to yield the clearest cut between clusters and showed a significant association with the patient survival.

Correlation between proteomic subtypes and clinical features

For the purpose of measuring correlations between proteomic subtypes and clinical features, Fisher's exact test was performed on histological subtypes, genomic alterations, gender, smoke status, alcohol habit, vessel invasion, fascicular invasion, metastasis, lymph node metastasis and TNM stage.

Survival analysis

Kaplan–Meier survival curves (log-rank test) were used to determine the overall survival (OS) and progression-free survival (PFS) of proteomic subtypes and patients. The coefficient value, which is equal to ln (HR), was calculated using Cox proportional hazards regression analysis. *p* values less than 0.05 were considered significantly different and selected for Cox regression multivariate

analysis. Prior to the log-rank test of a given protein, phosphoprotein, or phosphosite, survminer (version 0.2.4, R package) with maxstat (maximally selected rank statistics; http://r-addict.com/2016/11/21/Optimal-Cutpo int-maxstat.html) was used to determine the optimal cutoff point for the selected samples according to a previous study[100]. OS curves were then calculated (KaplanMeier analysis, log-rank test) based on the optimal cutoff point.

Effects of copy number alterations

SCNAs affecting mRNA and protein/phosphoprotein abundance in either "*cis*" (within the same aberrant locus) or "trans" (remote locus) mode were visualized by multiOmicsViz (R package)[101] (diagonal patterns in **Fig. 2A**). Spearman's correlation coefficients and associated multiple-test adjusted *p* values were calculated for all CNV–mRNA pairs, CNV-protein pairs and CNV-phosphoprotein pairs, respectively. Tthe usage of the *cis* effect in this work was followed by the definition provided in the previous published research[101], which defined the impact of copy number alteration (CNA) on the same loci protein or mRNA abundance as the cis effect, and the trans effect was defined as chromosomal loci whose alteration is significantly associated with abundance changes of many transcripts or proteins at other loci.

Defining cancer-associated genes

Cancer-associated genes (CAG) were compiled from genes defined by Bailey et al.[102] and cancer-associated genes listed in Mertins et al. [103] and adapted from Vogelstein et al.[104]. Gene Set Enrichment Analysis (GSEA) GSEA was performed by the GSEA software (http:// software.broadinstitute.org/gsea/index.jsp). Gene sets including KEGG, GO Biological Process (BP), Reactome, and HALLMARK downloaded from the Molecular Signatures Database (MSigDB v7.1, http://software.broad institute.org/gsea/msigdb/index.jsp) were used.

Immune subtype analysis

The immune score, stromal score and tumor purity were inferred using the R package ESTIMATE v1.0.11 using transcriptome data (**Supplementary Table S5c**)[105]. The abundances of 64 different cell types for AMPAC samples in protein level were computed via xCell (https:// xcell.ucsf.edu/) (**Supplementary Table S5a**). Based on these 64 signatures, consensus clustering was performed in

order to identify groups of samples with similar immune/stromal characteristics. Consensus clustering was performed using the R package ConsensusClusterPlus[98]. Consensus Cluster Plus parameters were reps = 1000, pItem = 0.8, pFeature = 1, clusterAlg = “pam,” distance = “spearman.” As summarized in **Fig.7,** the clustering analysis of the tumors (vertical column) by xCell score (horizontal rows) divided 198 samples into three immune clusters. A consensus matrix with k = 3 appeared to have the clearest cut between clusters and showed significant association with the patients’ survival.

Immunohistochemistry (IHC)

Formalin-fixed, paraffin-embedded tissue sections of 10 µM thickness were stained in batches for detecting HADH, MTAP, PCNA, ANO1, PRKCI, PDGFRB, CD34, ACLY, FASN, PRKDC, IL-6, STAT1, CD4 in a central laboratory at the Zhongshan Hospital according to standard automated protocols. Deparaffinization and rehydration were performed, followed by antigen retrieval and antibody staining. HADH, MTAP, PCNA, ANO1, PRKCI, PDGFRB, CD34, ACLY, FASN, PRKDC, IL-6, STAT1 and CD4 IHC were performed using the Leica BONDMAX auto staining system (Roche). HADH Polyclonal antibody (Proteintech, Cat No. 19828-1-AP), MTAP Polyclonal antibody (Proteintech, Cat No. 11475-1-AP), PCNA Polyclonal antibody (Proteintech, Cat No. 10205-2-AP), ANO1/TMEM16A Polyclonal antibody (Proteintech, Cat No. 12652-1-AP), PKC Iota Polyclonal antibody (Proteintech, Cat No. 13883-1-AP), PDGFR beta Polyclonal antibody (Proteintech, Cat No. 13449-1-AP), CD34 Polyclonal antibody (Proteintech, Cat No. 14486-1-AP), ACLY Monoclonal antibody (Proteintech, Cat No. 15421-1-AP), FASN Monoclonal antibody (Proteintech, Cat No. 66591-1-Ig), DNA-PKcs Polyclonal antibody (Proteintech, Cat No. 19983-1-AP), IL-6 Polyclonal antibody (Proteintech, Cat No. 21865-1-AP), STAT1 Monoclonal antibody (Proteintech, Cat No. 66545-1-Ig), and CD4 Monoclonal antibody (Proteintech, Cat No. 67786-1-Ig) were introduced, followed by detection with a Bond Polymer Refine Detection DS9800 (Bond). Slides were imaged using an OLYMPUS BX43 microscope (OLYMPUS) and processed using a ScanScope (Leica).

Cell lines

Human ampullary adenocarcinoma cell lines including SNU-869 and SNU-478 were obtained for Chinese Academy of Science (Shanghai, China). All cell lines were routinely tested for mycoplasma contamination and authenticated by Short Tandem repeat (STR) profiling. Cells were maintained in recommended medium, Roswell Park Memorial Institute-1640 (RPMI-1640, Corning) or Dulbecco's modified Eagle's medium (DMEM, ATCC) supplemented with 10% fetal bovine serum (FBS, Sigma - Aldrich) and 1% penicillin–streptomycin antibiotic (Sigma - Aldrich) and incubated at 37 °C and 5% CO_2 in a humidified atmosphere in an incubator.

Plasmids

The sequence of human PDGFRB, ANO1 and PRKCI open reading frame was obtained using Polymerase chain reaction (PCR) from CDNA. The PCR fragment was inserted into pCMV-N-Flag, pcDNA3.1 Myc HisA by the recombinant method and was confirmed by sequencing identification.

Cell transfections

Plasmid transfections were carried out by the polyethylenimine (PEI), Lipofectamine 3000 (Invitrogen), and Lipofectamine 2000 (Invitrogen) methods. In the PEI transfection method, 400 μL of DMEM (serum-free medium) and the plasmid were placed in an empty EP tube and PEI was added into the medium. The mixture was incubated for 15 min. Meanwhile, the cell culture medium was replaced with fresh 10% FBS medium. After 15 min, the mixture was added to the cells, and the fresh medium was replaced after 12–16 h. After 36–48 h, the transfection was completed. In the Lipofectamine 3000 transfection method, DMEM (250 μL) was added to two empty EP tubes and Lipofectamine 3000 was added to one of the tubes and mixed for 5 min. The plasmid and P3000 were added in the other tube and then added to the medium containing Lipofectamine 3000, mixed, and allowed to stand for 5 min. Meanwhile, the cell culture medium was replaced with fresh 10% FBS medium. After 5 min, the mixture was added to the cells, and the fresh medium was replaced after 12 h. After 36–48 h, the transfection was completed and the cells were treated. In the Lipofectamine 2000 transfection method, 125 μL of DMEM (serum-free medium) and the siRNA were placed in an empty EP tube.125 μL of DMEM (serumfree medium) and the Lipofectamine 2000 were placed in

another empty EP tube then added to the medium containing siRNA. The mixture was incubated for 5 min. Meanwhile, the cell culture medium was replaced with fresh 10% FBS medium. After 5 min, the mixture was added to the cells. After 36–48 h, the transfection was completed.

Gene silencing

To generate cells stably knockdown for HADH, MTAP were transfected into cells, using pCMV-VSVG and pCMV-Gag as packaging plasmids. Twenty-four hours after transfection, the virus supernatant was collected to infect target cells. Puromycin was used to select stable cells for ~ 7days.

HADH siRNA-homo-sense:5′-GGACTGGATACTACGAAGTTC-3′

HADH siRNA-homo-antisense:5′-GAACTTCGTAGTATCCAGTCC-3′

MTAP siRNA-homo-sense:5′-AAAAUUAAGGCAUCAGAUGGC-3′

MTAP siRNA-homo-antisense:5′-CAUCUGAUGCCUUAAUUUUGG-3′

Transwell migration assays

Cell migration assays were performed with 24-well transwells (8-μm pore size, Falcon). In total, 1.5×10^5 transfected cells were suspended in serum-free DMEM medium and added to the upper chamber, and 700μL DMEM with 10% FBS was placed in the lower chamber. After 16 h of incubation, cells on the lower surface of membrane were fixed in 4% paraformaldehyde and stained with crystal violet. Cells in six microscopic fields were counted and photographed.

AMPAC cells proteome

For the proteomic analysis of AMPAC cells, cells were lysed in lysis buffer (8 M Urea, 100 mM Tris Hydrochloride, pH 8.0) containing protease and phosphatase Inhibitors (Thermo Scientific) followed by 1 min of sonication (3 s on and 3 s off, amplitude 25%). The lysate was centrifuged at 14,000g for 10 min and the supernatant was collected as whole tissue extract. Protein concentration was determined by Bradford protein assay. Extracts from each sample (500 μg protein) was reduced with 10 mM dithiothreitol at 56 °C for 30 min and alkylated with 10 mM iodoacetamide at room temperature (RT) in the dark for additional 30 min. Samples were then digested using the filter-aided proteome preparation (FASP) method with trypsin.

Briefly, samples were transferred into a 30kD Microcon filter (Millipore) and centrifuged at 14,000g for 20 min. The precipitate in the filter was washed twice by adding 300μL washing buffer (8 M urea in 100 mM Tris, pH 8.0) into the filter and centrifuged at 14,000g for 20 min. The precipitate was resuspended in 200μL 100 mM NH4HCO3. Trypsin with a protein-to enzyme ratio of 50:1 (w/w) was added into the filter. Proteins were digested at 37 °C for 16 h. After tryptic digestion, peptides were collected by centrifugation at 14,000g for 20 min and dried in a vacuum concentrator (Thermo Scientific). Dried peptides were then used for proteomic analysis.

Immunoprecipitation

For immunoprecipitation, cells were lysed with 0.5% NP-40 buffer containing 50 mM Tris–HCl (pH 7.5), 150 mM NaCl, 0.3% NONIDET P-40, 1 μg mL − 1 aprotinin, 1 μg mL − 1 leupeptin, 1 μg mL− 1 pepstatin, and 1 mM PMSF. Cell lysates were incubated with Flag beads (Sigma) for 3 h at 4 °C. The binding complexes were washed with 0.5% NP-40 buffer and mixed with loading buffer for SDS-PAGE.

IP-MS for ANO1

The AMPAC SNU-478 cell line (SNU-478-*ANO1*-OE and SNU-478-vector) were lysed on ice in 0.5% NETN buffer (0.5% Nonidet P-40, 50 mM Tris-HCl (pH 7.4), 150 mM NaCl, 1 mM EDTA, and protease inhibitor mixture). After the removal of insoluble cell debris by highspeed centrifugation, protein concentration was then determined by Braford assay. Then 2 mg proteins were incubated with ANO1/TMEM16A Polyclonal antibody (1:100 dilution, Proteintech, Cat No. 12652-1-AP) and rotated overnight at 4 °C. Further, 20 μl Pre-wash magnetic beads (Protein A Magnetic Beads, #73778) were added for another 20 min incubation at room temperature. Pellet beads using magnetic separation rack. Wash pellets five times with 500 μl of 1X cell lysis buffer. Keep on ice between washes. Beads were further washed twice with ddH2O, and three times with 50 mM NH4HCO3. Then, "on-bead" tryptic digestion was performed at 37 °C overnight. The peptides in the supernatant were collected by centrifugation and dried in a speed vacuum (Eppendorf). Lastly, the samples were redissolved in loading buffer containing 0.1% formic acid before being subjected to MS.

Quantitative RT-PCR

The Superscript III RT kit (Invitrogen) was used with random 3 hexamer primers to produce cDNA from 4 µg total RNA. ACTIN was used as the endogenous control for samples. All primers for analysis were synthesized by TSINGKE Biological Technology (Shanghai). The analysis was performed by using an Applied Biosystems 7900HT Sequence Detection System, with SYBR green labeling.

QPCR-ANO1-F: 5′-CAAGT TTGGC TACAG CACGC-3′

QPCR-ANO1-R: 5′-AGACT AGGGA GCGAC GAAGT-3′

QPCR-PRKCI-F: 5′-GACGC AGGAG GTGTC TTGG-3′

QPCR-PRKCI-R: 5′-CTTGG CTTGG AAAGT GTGGC-3′

QPCR-PDGFRB-F: 5′-CCATC AGCAG CAAGG CGA-3′

QPCR-PDGFRB-R: 5′-CCAGA AAAGC CACGT TGGTG-3′

QPCR-HADH-F: 5′-AACTC GGGTT TGGGC TTTTC-3′

QPCR-HADH-R: 5′-TTTAA GGATG GGCTG GGCTG-3′

QPCR-MTAP-F: 5′-CGTGA AGGTG AGATG AGCCC-3′

QPCR-MTAP-R: 5′-TGTTC GCCTG GTAGT TGACC-3′

Cell proliferation assay

Different groups of cells (2000 cells/well) were seeded into 96-well plates. At the indicated detection times, CCK8 reagent was added into each well. The plates were incubated at 37 °C for 1 h, and then, absorbance of the 96-well plates was detected at a wavelength of 450 nm.

Xenograft tumorigenesis experiments

Different groups of ampullary adenocarcinoma cells (5 × 106) were re-suspended in PBS and injected subcutaneously (SC) into the right flank of 5-week-old BALB/c-nude mice. The weight and the tumor diameter of each mouse were measured every week. Tumor volume (mm3) was calculated as follows: (shortest diameter)2 × (longest diameter) × 0.5. Four weeks later all mice were killed.

Quantification and statistical analysis

Statistical details of experiments and analyses were noted in the figure legends and

supplementary tables. Standard statistical tests were used to analyze the association between clinical information and multi-omics data. Student t-test, Wilcoxon rank-sum test, one-way ANOVA, and Kruskal–Wallis test were used for continuous data; Fisher's exact test and Chi-Square test was used for categorical data. The Benjamini–Hochberg adjusted *p* values of differentially expressed RNA/proteins/phosphoproteins were calculated. Log-rank tests and Kaplan–Meier survival curves were used to compare the overall survival and progression free survival. All statistical tests were two-sided, and statistical significance was considered when $p < 0.05$. Variables associated with survival were identified using univariate Cox proportional hazards regression models. The correlation between two sets of data was calculated using Spearman's correlation. All the analyses of clinical data were performed in R (version 4.0.2) and GraphPad Prism 8. For functional experiments, each was repeated at least three times independently, and results were expressed as mean ± standard error of the mean (SEM). Statistical analysis was performed using GraphPad Prism 8.

Availability of data and materials

The proteomic data and phosphoproteomic data (Mass Spectrum raw data and the Masort output tables) generated in this research have been deposited in the ProteomeXchange Consortium via the iProX partner repository (http://www.iprox.cn/) under Project ID IPX0002195000. The raw WES data and Transcriptomic data have been deposited in the National Genomics Data Center (GSA) database under accession code HRA005749 and HRA005872, respectively. The raw sequencing data are available under controlled access due to data privacy laws related to patient consent for data sharing and the data should be used for research purposes only. Access can be obtained by approval via their respective DAC (Data Access Committees) in the GSA-human database. According to the guidelines of GSA-human, all non-profit researchers are allowed access to the data and the Principle Investigator of any research group is allowed to apply for Controlled access of the data. The user can register and login to the GSA database website (https://ngdc.cncb.ac.cn/gsa-human/) and follow the guidance of "Request Data" to request the data step by step (https:// ngdc.cncb.ac.cn/gsa-human/document/GSAHuman_Request_Guide_ for_Users_us.pdf). The approximate response time for accession requests is about 2 weeks. The access authority can be obtained for Research Use Only. The user can also contact the corresponding author directly. Once access has been

granted, the data will be available to download for 3 months. The remaining data are available within the Article, Supplementary Information, or Source Data file. Source data are provided with this paper. Human reference genome (hg19 assembly) was downloaded from NCBI (https://www.ncbi.nlm.nih.gov/). The information of kinase-substrate relationships were available in PhosphoSite [https:// www.phosphosite.org/homeAction.action]38, Phospho.ELM [http:// phospho.elm.eu.org/dataset.html]36, and PhosphoPOINT [http:// kinase.bioinformatics.tw/]37, Source data are provided with this paper.
